# CRISPR–Cas9-based functional interrogation of unconventional translatome reveals human cancer dependency on cryptic non-canonical open reading frames

**DOI:** 10.1038/s41594-023-01117-1

**Published:** 2023-11-06

**Authors:** Caishang Zheng, Yanjun Wei, Peng Zhang, Kangyu Lin, Dandan He, Hongqi Teng, Ganiraju Manyam, Zhao Zhang, Wen Liu, Hye Rin Lindsay Lee, Ximing Tang, Wei He, Nelufa Islam, Antrix Jain, Yulun Chiu, Shaolong Cao, Yarui Diao, Sherita Meyer-Gauen, Magnus Höök, Anna Malovannaya, Wenbo Li, Ming Hu, Wenyi Wang, Han Xu, Scott Kopetz, Yiwen Chen

**Affiliations:** 1 Department of Bioinformatics and Computational Biology, the University of Texas MD Anderson Cancer Center, Houston, TX USA; 2 Department of Experimental Radiation Oncology, the University of Texas MD Anderson Cancer Center, Houston, TX USA; 3 Department of Biochemistry and Molecular Biology, McGovern Medical School, the University of Texas Health Science Center at Houston, Houston, TX USA; 4 Center for Infectious and Inflammatory Diseases, Texas A&M Health Science Center, Institute of Biosciences of Technology, Houston, TX USA; 5 Department of Quantitative Health Sciences, Lerner Research Institute, Cleveland Clinic Foundation, Cleveland, OH USA; 6 Department of Translational Molecular Pathology, the University of Texas MD Anderson Cancer Center, Houston, TX USA; 7 Department of Epigenetics and Molecular Carcinogenesis, the University of Texas MD Anderson Cancer Center, Houston, TX USA; 8 Mass Spectrometry Proteomics Core, Baylor College of Medicine, Houston, TX USA; 9 Department of Melanoma Medical Oncology, the University of Texas MD Anderson Cancer Center, Houston, TX USA; 10 Department of Cell Biology, Duke University Medical Center, Durham, NC USA; 11 Duke Regeneration Center, Duke University Medical Center, Durham, NC USA; 12 Department of Orthopedic Surgery, Duke University Medical Center, Durham, NC USA; 13 Verna and Marrs McLean Department of Biochemistry and Molecular Biology, Baylor College of Medicine, Houston, TX USA; 14 Department of Molecular and Cellular Biology, Baylor College of Medicine, Houston, TX USA; 15 Dan L. Duncan Cancer Center, Baylor College of Medicine, Houston, TX USA; 16 Graduate School of Biomedical Sciences, University of Texas MD Anderson Cancer Center and UTHealth, Houston, TX USA; 17 Quantitative Sciences Program, MD Anderson Cancer Center UTHealth Graduate School of Biomedical Sciences, Houston, TX USA; 18 Genetics and Epigenetics Program, MD Anderson Cancer Center UTHealth Graduate School of Biomedical Sciences, Houston, TX USA; 19 Department of Gastrointestinal Medical Oncology, University of Texas MD Anderson Cancer Center, Houston, TX USA; 20 Present Address: Key Laboratory of RNA Biology, Center for Big Data Research in Health, Institute of Biophysics, Chinese Academy of Sciences, Beijing, China; 21 Present Address: Department of Gastrointestinal Medical Oncology, University of Texas MD Anderson Cancer Center, Houston, TX USA; 22 Present Address: Sema4, Inc., Stamford, CT USA; 23 Present Address: MOE Key Laboratory of Metabolism and Molecular Medicine, Department of Biochemistry and Molecular Biology, School of Basic Medical Sciences, Fudan University, Shanghai, China

**Keywords:** Translation, Epigenetics, Cancer, Chromatin, High-throughput screening

## Abstract

Emerging evidence suggests that cryptic translation beyond the annotated translatome produces proteins with developmental or physiological functions. However, functions of cryptic non-canonical open reading frames (ORFs) in cancer remain largely unknown. To fill this gap and systematically identify colorectal cancer (CRC) dependency on non-canonical ORFs, we apply an integrative multiomic strategy, combining ribosome profiling and a CRISPR–Cas9 knockout screen with large-scale analysis of molecular and clinical data. Many such ORFs are upregulated in CRC compared to normal tissues and are associated with clinically relevant molecular subtypes. We confirm the in vivo tumor-promoting function of the microprotein SMIMP, encoded by a primate-specific, long noncoding RNA, the expression of which is associated with poor prognosis in CRC, is low in normal tissues and is specifically elevated in CRC and several other cancer types. Mechanistically, SMIMP interacts with the ATPase-forming domains of SMC1A, the core subunit of the cohesin complex, and facilitates SMC1A binding to *cis*-regulatory elements to promote epigenetic repression of the tumor-suppressive cell cycle regulators encoded by *CDKN1A* and *CDKN2B*. Thus, our study reveals a cryptic microprotein as an important component of cohesin-mediated gene regulation and suggests that the ‘dark’ proteome, encoded by cryptic non-canonical ORFs, may contain potential therapeutic or diagnostic targets.

## Abstract (web summary)

The authors show that the lncRNA-derived microprotein SMIMP, which is shown to promote tumor formation, regulates cohesin core subunit binding to *cis*-regulatory elements and alters the expression of tumor-suppressive cell cycle regulators.

## Main

Systematic transcriptome profiling in human cells^1,2^ has uncovered prevalent transcription of over 70% of the human genome. Many transcripts in the human transcriptome lack ORFs that are recognizable by traditional sequence-based bioinformatic methods and are thus called noncoding RNA (ncRNA). Growing evidence supporting active translation of cryptic non-canonical ORFs within ncRNA has blurred the boundary between ncRNA and protein-coding genes. High-resolution genome-wide measurements of translation (that is, the translatome) by the ribosome profiling (ribo-seq) technique^3,4^ has revealed pervasive cryptic translation beyond the conventional annotated translatome^4–6^. These cryptic translations include ones starting at alternative translation-initiation sites within annotated protein-coding genes as well as those occurring completely in traditionally noncoding regions, such as 5′ untranslated regions (UTRs) (upstream ORFs; uORFs), 3′ UTRs (downstream ORFs), long (>200 bp) ncRNA (lncRNA) and pseudogene RNA^5,7,8^. Microproteins (also termed micropeptides, <100 amino acids) encoded by lncRNA have been shown to play important developmental or physiological roles in evolutionarily distant species^9–13^. Moreover, different from the traditional view of uORFs as *cis*-acting translational control elements, it has been recently discovered that the microproteins encoded by uORFs can form stable complexes with the main protein encoded by the same mRNA to perform important functions^14^.

Despite increasing appreciation of the functional importance of cryptic non-canonical ORFs in development, physiology and disease, the role and functional mechanism of human cryptic non-canonical ORFs in complex diseases such as cancer remain largely unknown. To fill this gap, we applied an integrative multiomic strategy^15^ combining ribo-seq, a CRISPR–Cas9 pooled screen^16^ with computational analysis of the Cancer Genome Atlas (TCGA)^17^ and/or Genotype–Tissue Expression (GTEx)^18^ data to identify cryptic ORFs that may be functionally important and potentially clinically relevant in CRC, the third most commonly diagnosed cancer in men, the second most common cancer in women and the second mostly deadly cancer worldwide. We further characterized the function and mechanism of a cryptic *ELFN1*-*AS1*-encoded microprotein that was identified from our screen. Because of its interaction with structural maintenance of chromosomes protein (SMC)1A, it was named SMC1A-interacting microprotein (SMIMP).

## Results

### Identifying CRC dependency on cryptic non-canonical ORFs

**Fig. 1:**
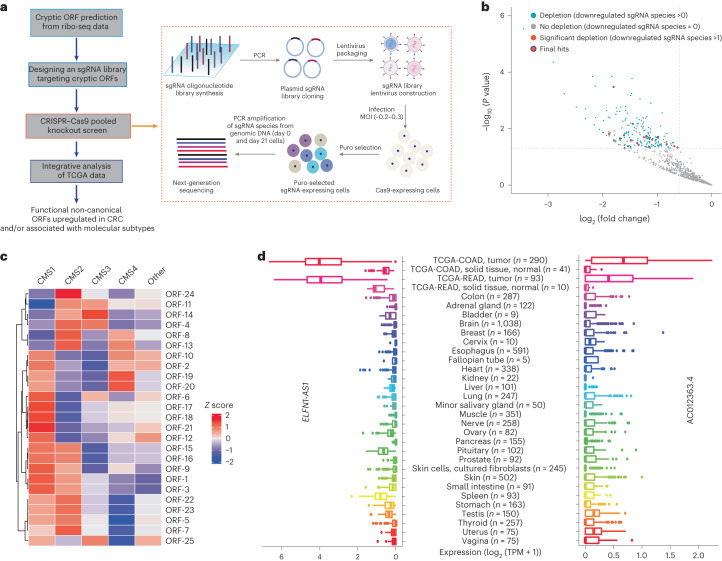
Identification of CRC dependency on cryptic ORFs. **a**, Schema depicting the integrative strategy for identifying CRC dependency on cryptic ORFs. MOI, multiplicity of infection; puro, puromycin. **b**, Scatterplot showing the statistical significance (−log_10_ (*P* value)) and the magnitude of change (log_2_ (fold change)) between day 21 and day 0 for the representative negatively selected sgRNA of the corresponding ORFs. *P* values were determined by the Wald test implemented in DESeq2 (Methods). ORFs with zero, at least one and at least two significantly depleted sgRNA species are colored in gray (no depletion), blue (depletion) and red (significant depletion), respectively. After controlling for the potential effect on neighboring genes (Methods), ORFs that had at least two significantly depleted sgRNA species and that were upregulated in COAD compared with normal colon tissue were selected as the final hits. **c**, Heatmap showing row-wise *Z*-score-normalized average expression of the identified cryptic non-canonical ORFs in four major CRC molecular subtypes (CMS1–CMS4) and the CRC tumors that do not fall into CMS1–CMS4 (other), based on TCGA data. **d**, The expression of *ELFN1*-*AS1* and AC012363.4 in CRC adenocarcinoma (TCGA-COAD), READ (TCGA-READ), the corresponding normal tissues of COAD and READ in TCGA and different types of normal tissues in GTEx. The first and third quartiles are depicted by the bottom and top edges of the box, respectively. The median is indicated by the line that divides the box into two sections. Extending from the box, the whiskers illustrate the range between the bottom 5% and 25%, as well as the top 25% and 5%. Any outliers are displayed as individual points. The sample size is indicated after each tumor or normal tissue type. TPM, transcripts per million.

**Extended Data Fig. 1:**
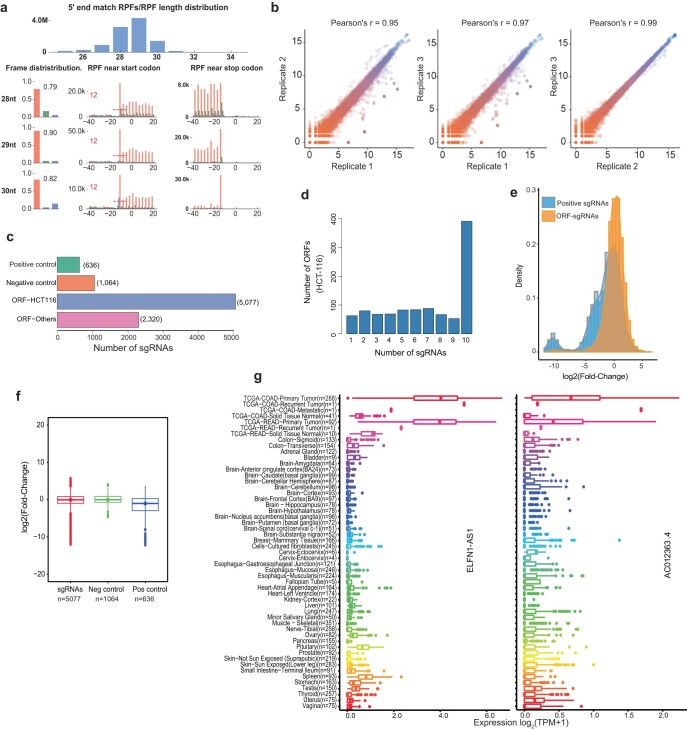
CRISPR/Cas9 screens for identifying CRC dependency on cryptic ORFs. **a**, Ribo-seq data quality control. Upper panel: length distribution of the RPFs uniquely mapped to the annotated protein-coding regions. Lower panel: different quality profiles/metrics for RPFs uniquely mapped to the annotated protein-coding regions. Each row shows the RPFs with indicated length. Column 1: RPF count distribution across 3 reading frames across the annotated codons; Column 2: RPF count distribution near the annotated TISs; Column 3: RPF count distribution near the annotated stop codons. **b**, Scatter plot showing the correlation between ribo-seq replicates. **c**, Bar graph showing the number of sgRNAs targeting the cryptic ORFs identified from ribo-seq data, and the positive/negative control sgRNAs. **d**, Bar graph showing the distribution of the number of ORFs in HCT-116 cells over the number of targeting sgRNAs. **e**, The histograms showing the distribution of log_2_(Fold-Change) between day 21 and day 0 for sgRNAs targeting the cryptic ORFs (orange) and the positive control genes (blue) in the CRISPR/Cas9 screen. **f**, The box plot showing the log_2_(Fold-Change) between day 21 and day 0 for sgRNAs targeting the cryptic ORFs (red) (n = 5,077), the negative controls (green) (n = 1,064) and the positive control genes (blue)(n = 636). The bottom and top edges of the box represent the first and third quartiles. The median is indicated by the line dividing the box into 2 parts. The whiskers illustrate the values between the bottom 5% and 25% or between the top 25% and 5%. Any outliers are displayed as individual points. **g**, The expression of ELFN1-AS1 and AC012363.4 in the CRC adenocarcinoma (TCGA-COAD), the rectum adenocarcinoma (TCGA-READ), the corresponding normal tissues of COAD and READ in TCGA, and normal tissues with refined tissue types in GTEx. The sample size was indicated after each tumor/normal tissue type.

First, ribo-seq was performed to map the translatome in the CRC cell line HCT-116 as described previously^7,15^ (Fig. 1a and the Methods). Quality-control analysis of ribo-seq data (Methods) showed characteristics of data with good quality, including a peaked length of around 30 nucleotides in ribosome-protected fragment (RPF) length distribution (Fig. 1a), noticeable subcodon phasing or three-nucleotide periodicity of the RPF count across three reading frames and a clear increase or reduction in RPF count near annotated translation-initiation sites or translation-termination sites, respectively (Extended Data Fig. 1a). The gene-level RPF count also showed a significant correlation (Pearson’s *r* > 0.95, *P* < 2.2 × 10^−16^) between replicates (Extended Data Fig. 1b). Ribo-seq data-driven translation-initiation site hunter (Ribo-TISH)^7^ was then used to predict actively translating cryptic ORFs from ribo-seq data (Methods). To avoid the confounding effects of *cis*-acting regulation and parent genes of pseudogenes, we excluded ORFs that are part of the annotated protein-coding genes or pseudogenes from our study. We conducted a pooled CRISPR–Cas9 knockout screen on a total of 1,046 non-canonical lncRNA-encoded ORFs with the ATG start codon identified by Ribo-TISH^7^ in HCT-116 cells (Fig. 1a and the Methods) to systematically identify those that may critically contribute to cell growth and/or survival (fitness). We first used the sequence scan for CRISPR (SSC) method^19^ to design single-guide RNA (sgRNA) and generated a pooled sgRNA library (Fig. 1a and the Methods) that contained 7,397 sgRNA species targeting the non-canonical lncRNA-encoded ORFs identified from ribo-seq data (4,987 for the ORFs in HCT-116 cells and 2,410 sgRNA species for the ORFs uniquely identified in two other breast cancer cell lines) as well as 636 positive and 1,064 negative controls (Extended Data Fig. 1c,d and Supplementary Table 1). The screen was then performed in HCT-116 cells stably expressing wild-type *Streptococcus pyogenes* Cas9 (SpCas9), following a protocol akin to those in our previous studies^15,20–22^. In brief, cells were transduced with lentiviral vectors containing the sgRNA library and subsequently subjected to puromycin selection. Puromycin-selected cells were then passaged for 21 d, and changes in abundance of individual sgRNA species between day 0 and day 21 were measured using next-generation sequencing to identify critical ORFs for cell fitness (Methods). As anticipated, sgRNA species targeting positive-control core essential genes showed a significant decrease in abundance in the final (day 21) cell populations compared to the initial (day 0) ones, confirming the functionality of the positive controls (Extended Data Fig. 1e,f).

There were 56 ORFs with at least two significantly depleted sgRNA species (sgRNA level, log_2_ (fold change) − log_2_ (1.5) and *P* < 0.05) that were considered as the candidate hits (Fig. 1b). Because of the short length of the non-canonical ORFs, the sequence space for sgRNA design was more limited for them compared with that for the annotated protein-coding genes. As a result, some of the designed sgRNA species may impact the UTRs or coding sequences (CDS) of the neighboring protein-coding genes. To control for the potential effect on neighboring coding genes, we first identified those sgRNA species that may impact the UTR or CDS of the annotated coding genes. As the majority of Cas9-mediated changes are relatively small in size (<15 bp)^23^, we considered an sgRNA to have a potential effect on the UTR or CDS of a coding gene if its putative cutting site is within 15 bp of the UTR or CDS of the coding gene. For a candidate non-canonical ORF hit, we considered it a valid one if there were at least two significantly depleted sgRNA species after removing (1) the sgRNA species that potentially affect the CDS of the annotated coding genes (regardless of whether the gene is essential or not) and (2) the sgRNA species that may affect the UTRs of the annotated coding genes that are essential genes in HCT-116 cells, based on the Project Score database^24^. After applying this filter, there were 49 ORFs that remained as valid hits. We further selected the hits showing significantly elevated expression in colon adenocarcinoma (COAD) compared with normal colon tissues (log_2_ (fold change) ≥ log_2_ (1.2), false discovery rate (FDR) < 0.01; Fig. 1b), based on TCGA RNA-seq data, and removed redundant ORFs encoded by the same gene (Methods), resulting in a total of 25 non-canonical ORFs that represent the candidates for CRC dependency (Supplementary Table 1). CRC has a marked intertumor heterogeneity that contributes to heterogeneous drug responses and clinical outcomes. CRC can be classified into four major consensus molecular subtypes (CMSs, CMS1–CMS4) and a small set of unclassified tumors^25^. CMS1 is enriched for microsatellite instable tumors and is an immunogenic subtype; CMS2 has epithelial features with marked activation of WNT and MYC signaling; CMS3 has epithelial characteristics with evident metabolic dysregulation; and CMS4 is a mesenchymal subtype with pronounced transforming growth factor β activation, stromal invasion and angiogenesis. Over 60% of the 25 ORFs showed significantly higher expression in specific CMSs than in the rest of CRC tumors (log_2_ (fold change) ≥ 0.4, FDR < 0.05; Fig. 1c and Supplementary Table 1), including six in CMS1, seven in CMS2, two in CMS3 and two in CMS4, suggesting a diverse functional role of these ORFs in different subtypes.

**Fig. 2:**
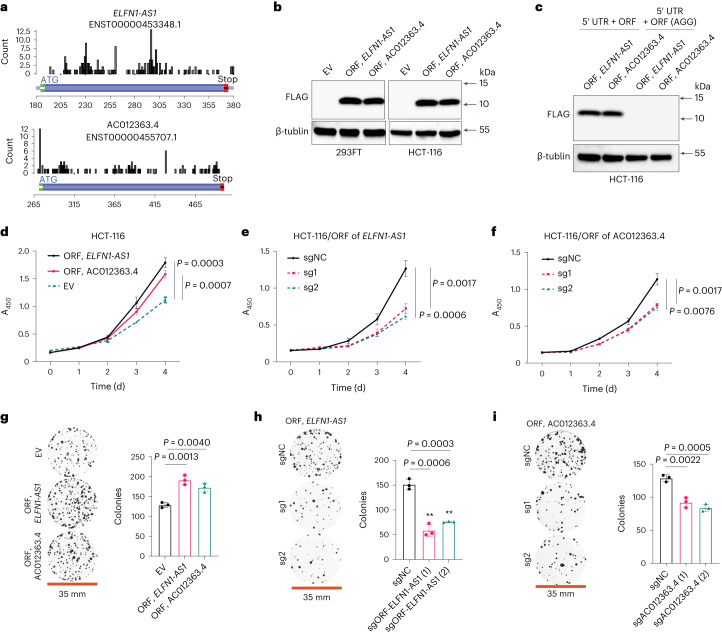
Validation of two ORF hits encoded by *ELFN1*-*AS1* and AC012363.4. **a**, Ribo-seq count profiles across the transcripts encoding ORFs of *ELFN1*-*AS1* and AC012363.4. **b**, ORFs of *ELFN1*-*AS1* and AC012363.4 were ectopically expressed with FLAG tag in 293FT and HCT-116 cells, and their expression was detected by western blot with an anti-FLAG antibody. EV, empty vector. **c**, Comparison between expression of the ORFs of *ELFN1*-*AS1* and AC012363.4 expressed with FLAG tag in the presence of the native 5′ UTR, with the wild-type (ATG) start codon versus the mutant one (AGG) in HCT-116 cells, by western blot. **d**–**f**, Growth of HCT-116 cells transduced with the negative-control EV, the complementary DNA (cDNA) overexpression vectors of the ORFs of *ELFN1*-*AS1* and AC012363.4 (**d**) or the negative-control sgRNA (sgNC) or gene-specific sgRNA species (sg1 and sg2) targeting the ORFs of *ELFN1*-*AS1* (**e**) and AC012363.4 (**f**) was monitored with the Cell Counting Kit-8 (CCK-8) assay. The absorbance at 450 nm (A_450_) of WST-8 formazan was measured each day for 4 d. **g**–**i**, Representative pictures of clonogenic growth and bar graphs quantifying the colonies formed by HCT-116 cells that were transduced with the EV control, the cDNA overexpression vector of individual ORFs (**g**) or the sgNC or sgRNA species targeting individual ORFs (**h**,**i**). Western blot data and pictures of clonogenic growth are representative of at least three independent experiments. Data in **d**–**i** are shown as mean ± s.d. (*n* = 3). *P* values were determined by an unpaired two-tailed Student’s *t*-test. Source data

**Extended Data Fig. 2:**
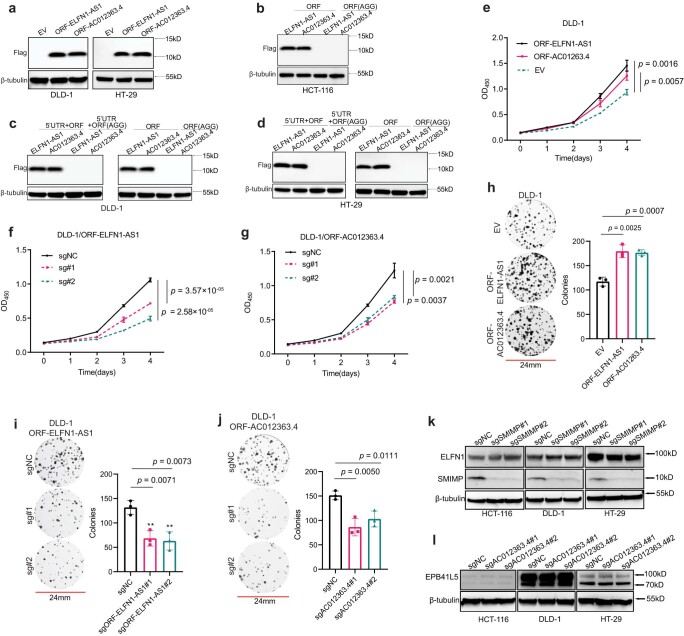
Characterization of ELFN1-AS1/AC012363.4 encoded ORFs. **a**, The FLAG-tagged ORF-ELFN1-AS1/-AC012363 was ectopically expressed in DLD-1 and HT-29 cells and their expressions were detected by western blot with an anti-FLAG antibody. **b**-**d**, The comparison of the FLAG-tagged ORF-ELFN1-AS1/-AC012363 expression in the presence/absence of the native 5’UTR, between wild-type and the mutant (AGG start codon) by western blot in (**b**) HCT-116, (**c**) DLD-1 and (**d**) HT-29 cells. **e-g**, The growth of the DLD-1 cells transduced with (**e**) the negative control empty vector (EV), the cDNA overexpression vector of ORF-ELFN1-AS1/ORF-AC012363.4, the negative control sgRNA (sgNC), or sgRNAs targeting (**f**) ORF-ELFN1-AS1 and (**g**) ORF-AC012363.4, was monitored with CCK-8 assay. The OD450 absorbance for WST-8 formazan was measured each day for 4 days. **h-j**, The representative pictures of clonogenic growth and the bar graph quantifying the colonies formed by the DLD- 1 cells that were transduced with (**h**) the EV control, the cDNA overexpression vector of individual ORFs, or (**i-j**) the sgNC/sgRNAs targeting the individual ORFs, after the cells were cultured for two weeks. **k**, **l**, The expression of (**k**) ELFN1 and (**l**) EPB41L5 was detected by western blot in the indicated CRC cancer cell lines that were transduced with the sgNC or sgRNAs targeting SMIMP, where β-tubulin was used as a loading control. Western blot data and the pictures of clonogenic growth are representative of at least three independent experiments. When applicable, data are shown as mean + /−standard deviation (SD), n = 3. *P*-values were determined by an unpaired two-tailed Student’s *t*-test. Source data

*ELFN1*-*AS1* and AC012363.4 were among the identified functional ORF-encoding lncRNA genes with the most cancer-specific expression in COAD and/or rectum adenocarcinoma (READ), that is, they not only showed much higher expression in COAD and/or READ than in the corresponding normal tissues, but they also showed a relatively low expression across different normal tissues (Fig. 1d and Extended Data Fig. 1g). They encode actively translated ORFs of 62 and 75 codons (Supplementary Table 1) and showed significantly higher expression in CMS2 (log_2_ (fold change) = 0.66, *P* < 5.2 × 10^−4^) and CMS1 (log_2_ (fold change) = 0.52, *P* < 0.013), respectively (Fig. 1c and Supplementary Table 1). With the ribo-seq data supporting their translation (Fig. 2a), we next tested whether these two lncRNA-encoded ORFs were able to produce a stable polypeptide via RNA translation by first ectopically expressing their CDS in the absence or presence of the native 5′ UTRs, with a 3′ end addition of FLAG epitope tags before the stop codon, and then detecting the translated polypeptide with an anti-FLAG antibody by western blot^7^. We consistently detected polypeptides produced by these two lncRNA-encoded ORFs in the CRC cell lines HCT-116, DLD-1 and HT-29 by western blot (Fig. 2b,c and Extended Data Fig. 2a–d). Importantly, when the ATG start codon of the ORFs was mutated to AGG (a non-ATG start codon with very poor translation-initiation efficiency^7,26^), production of the polypeptide was abolished (Fig. 2c and Extended Data Fig. 2b–d), indicating that these microproteins are indeed produced by translation of the corresponding ORFs.

To validate their functional importance in promoting CRC cell growth in vitro, we examined the gain-of-function phenotype by overexpressing individual ORFs in the CRC cell lines HCT-116 and DLD-1. Overexpression of either ORF led to enhanced growth of HCT-116 and DLD-1 cells in vitro (Fig. 2d and Extended Data Fig. 2e). Additionally, for each ORF, we selected the top two sgRNA species that exhibited the most potent growth-inhibitory effect in the CRISPR screen and found that transducing HCT-116 cells with either of the gene-specific sgRNA species inhibited the growth of these cells (Fig. 2e,f and Extended Data Fig. 2f,g). Overexpression or sgRNA-mediated knockout of either ORF promoted (Fig. 2g and Extended Data Fig. 2h) or impaired (Fig. 2h,i and Extended Data Fig. 2i,j) the clonogenic capacity of CRC cells. To rule out the possibility that the observed sgRNA-mediated growth or clonogenicity inhibition was caused by an sgRNA-induced effect on the neighboring protein-coding genes, we tested whether transducing HCT-116, DLD-1 and HT-29 cells with individual sgRNA species targeting *ELFN1*-*AS1* or AC012363.4 altered expression of their neighboring genes *ELFN1* or *EPB41L5*, respectively. We found that transducing these sgRNA species had little effect on the protein level of extracellular leucine-rich repeat and fibronectin type III domain-containing 1 (ELFN1) or erythrocyte membrane protein band 4.1-like 5 (EPB41L5) (Extended Data Fig. 2k,l), excluding the potential sgRNA-induced off-target effect on the neighboring protein-coding genes. In sum, these data indicate a tumor-promoting function of *ELFN1*-*AS1*- and AC012363.4-encoded microproteins in vitro. Importantly, the lncRNA gene AC012363.4 has not been reported to play a functional role in human cancer.

### The primate-specific lncRNA ELFN1-AS1 encodes a microprotein

**Extended Data Fig. 3:**
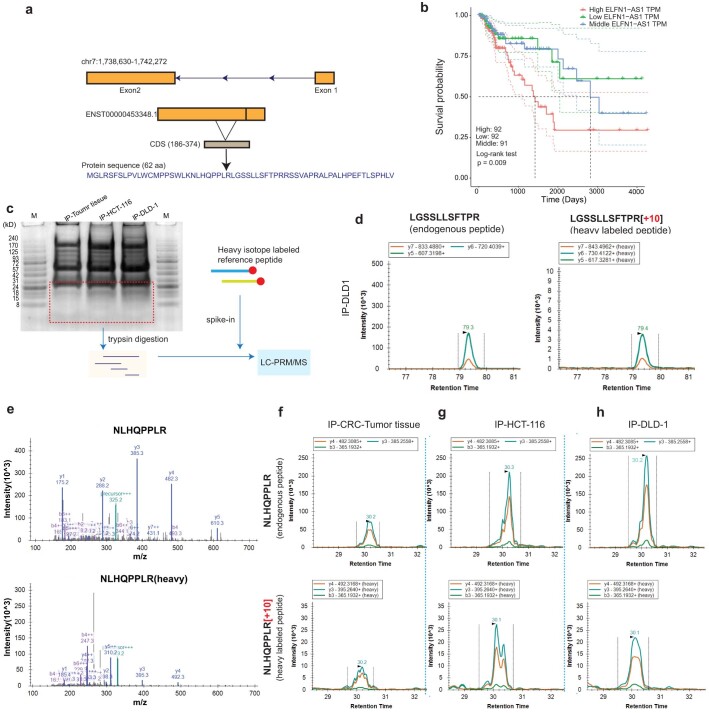
ELFN1-AS1 encodes a microprotein. **a**, The gene structure, genomic location of ELFN1-AS1 and the length and sequence of its encoded microprotein. The transcript of ELFN1-AS1 (ENST00000453348.1) encoding SMIMP has two exons and the CDS is in the exon 2. **b**, Higher ELFN1-AS1 expression was associated with worse COAD patient overall survival based on TCGA data. The Kaplan-Meier survival curves are plotted for three patient groups with high (top 1/3, n = 92), medium (middle 1/3, n = 91), and low (bottom 1/3, n = 92) ELFN1-AS1 expression in COAD tumors. The *P*-value was calculated based on log-rank test. **c**, A schematic of PRM-MS validation of the unique peptides derived from SMIMP with the spike-in heavy isotope labeled synthetic peptides. **d**, The top three ranked PRM-MS transition ions spectra of the SMIMP-derived tryptic peptide LGSSLLSFTPR (left) and the corresponding spike-in heavy isotope-labeled peptide (right) detected in the mixture of spike-in heavy isotope-labeled peptide and immunoprecipitated endogenously expressed SMIMP from DLD-1 cell lysate. **e**, The MS2 spectra of the SMIMP-derived tryptic peptide NLHQPPLR (top) and the corresponding heavy isotope-labeled peptide (bottom) detected by PRM-MS in the mixture of heavy isotope-labeled peptide and immunoprecipitated endogenously expressed SMIMP from HCT-116 cell lysate. **f-h**, The top three ranked PRM-MS transition ions spectra of the SMIMP-derived tryptic peptide NLHQPPLR (top) and the corresponding heavy isotope-labeled peptide (bottom) detected in the mixture of spike-in heavy isotope-labeled peptide and immunoprecipitated endogenously expressed SMIMP from the lysate of (**f**) CRC tumor tissues (Supplementary Table 3), (**g**) HCT-116 and (**h**) DLD-1 cells. [R], heavy isotope-labeled Arginine. Source data

We selected the 62-amino-acid-long *ELFN*-*AS1*-encoded microprotein SMIMP (Extended Data Fig. 3a) for further investigation because it exhibited a stronger tumor-promoting effect on CRC cells and higher RNA expression in COAD and READ tumors than the AC012363.4-encoded protein (Fig. 1d and Extended Data Fig. 1g). *ELFN1*-*AS1* is an evolutionarily new gene that originated de novo in the primate lineage^27^. By searching for homologous sequences of the human CDS corresponding to SMIMP in the genomes of non-human primate species (Methods), we found that the DNA sequence corresponding to human SMIMP is also highly similar to sequences in the chimpanzee, gorilla, orangutan, gibbon, bonobo, marmoset, squirrel monkey, baboon, proboscis monkey, rhesus macaque, green monkey, golden snub-nosed monkey and crab-eating macaque (Supplementary Fig. 1a). However, DNA sequences in non-human primate species either have a near-cognate start codon (ATC or ATT) or a truncation of the 5′ region, suggesting that not all homologous sequences in non-human species can produce a protein. A previous study showed that ELFN1-AS1 is among the lncRNA genes with the most significant upregulation in early-stage COAD^28^, suggesting that it might play an important role in early-stage COAD. We found that higher expression of *ELFN1*-*AS1* was significantly associated with shorter overall survival of patients with COAD in TCGA data (log-rank test, *P* = 0.009; Extended Data Fig. 3b).

**Fig. 3:**
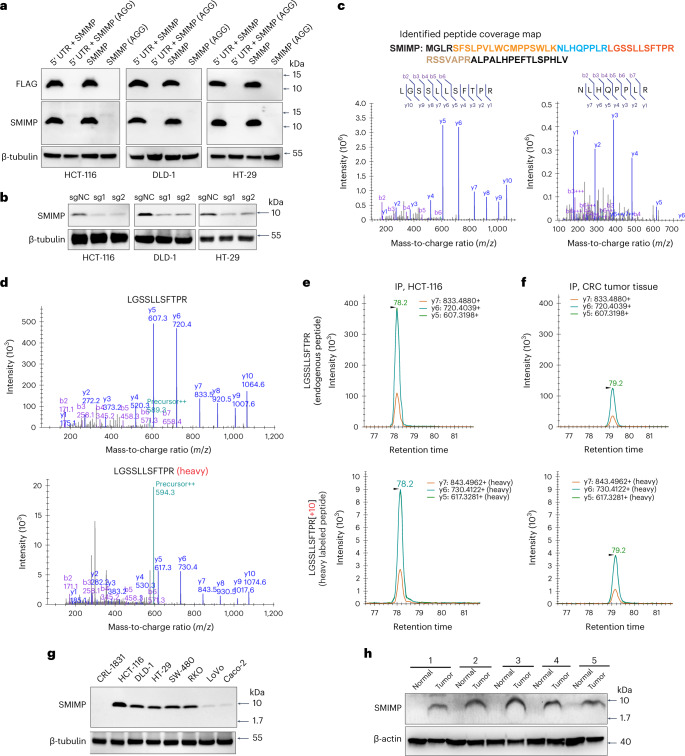
*ELFN1*-*AS1* encodes a microprotein upregulated in CRC tumors. **a**, In the presence or absence of the native 5′ UTR, FLAG-tagged SMIMP or the mutant one (AGG mutation in the start codon) was stably expressed in HCT-116, DLD-1 and HT-29 cells, and protein expression was determined by western blot with anti-FLAG and anti-SMIMP antibodies. **b**, Endogenous SMIMP expression was determined by western blot in the indicated CRC cancer cell lines transduced with negative-control sgRNA or sgRNA species targeting SMIMP; β-tubulin was used as a loading control. **c**, All constituent peptides of SMIMP that were identified by MS from IP of FLAG-tagged proteins in HCT-116 cells stably expressing FLAG-tagged SMIMP in the presence of the native 5′ UTR and MS^2^ spectral evidence for two of these peptides, LGSSLLSFTPR and NLHQPPLR. **d**, MS^2^ spectra of the SMIMP-derived tryptic peptide LGSSLLSFTPR (top) and the corresponding heavy isotope-labeled peptide (bottom) detected by PRM-MS in a mixture of heavy isotope-labeled synthetic peptide and immunoprecipitated endogenously expressed SMIMP from the HCT-116 cell lysate. **e**,**f**, The top three ranked PRM-MS transition ion spectra of the SMIMP-derived tryptic peptide LGSSLLSFTPR (top) and the corresponding spike-in heavy isotope-labeled peptide (bottom) detected in a mixture of spike-in heavy isotope-labeled peptide and immunoprecipitated endogenously expressed SMIMP from HCT-116 cell (**e**) and CRC tumor tissue (**f**) (Supplementary Table 3) lysate. [R], heavy isotope-labeled arginine. **g**, Western blot showing endogenous SMIMP expression in CRL-1831 and seven different CRC cell lines with an anti-SMIMP antibody; β-tubulin was used as a loading control. **h**, Western blot showing SMIMP expression in CRC tumor tissues and matched normal tissues (*n* = 5; Supplementary Table 3) with an anti-SMIMP antibody; β-actin was used as a loading control. Western blot data are representative of at least three independent experiments. Source data

A polyclonal antibody was generated to detect ectopically expressed FLAG-tagged SMIMP in the presence of its native 5′ UTR by western blot (Fig. 3a), whereas an ATG-to-AGG start codon mutation abolished the signal in the western blot, suggesting high specificity of this antibody. It was also able to detect the endogenous microprotein in CRC cells, with a reduced western blot signal when the microprotein was depleted by sgRNA (Fig. 3b).

The microprotein produced by the ORF ectopically expressed with FLAG tag in the presence of its native 5′ UTR was also confirmed using mass spectrometry (MS) (Fig. 3c, Supplementary Table 2 and the Methods). Using the spike-in heavy isotope-labeled synthetic peptides corresponding to the two peptides showing strong signal detected by MS for the ectopically expressed proteins (Methods), parallel reaction monitoring (PRM)-MS^29^ was performed on immunoprecipitation (IP) samples generated from CRC cell lines or patient tumor tissue (Extended Data Fig. 3c). PRM-MS data provided direct evidence that SMIMP is endogenously expressed (Fig. 3d–f, Extended Data Fig. 3d–h and Supplementary Table 2). To determine SMIMP subcellular localization, we performed subcellular fractionation followed by western blotting in CRC cell lines and patient tumor tissues and immunofluorescence staining of FLAG-tagged SMIMP with anti-FLAG antibody in cell lines. We found that this microprotein was localized in both the nucleus and the cytoplasm (Supplementary Fig. 1b–d).

**Extended Data Fig. 4:**
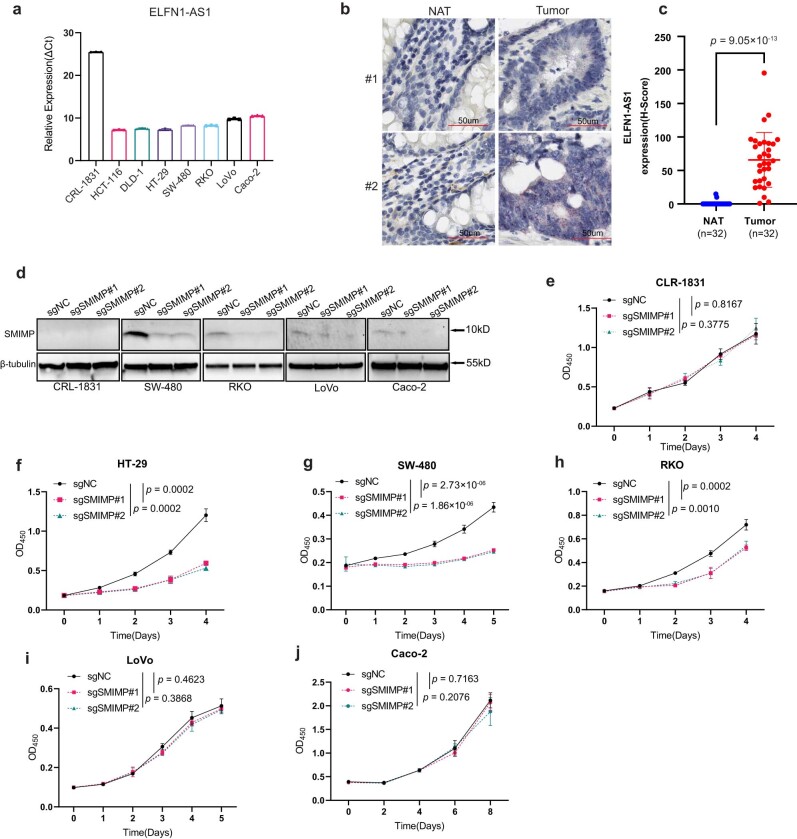
Characterizing SMIMP expression in cell lines and tumors, and its function in different cell lines. **a**, qRT-PCR analysis of the endogenous RNA expression of ELFN1-AS1 in immortalized colon epithelial cell line, CRL-1831, and 7 different CRC cancer cell lines, where GAPDH was used as an internal control. **b**, **c**, (**b**) The representative images of hematoxylin-stained tumor tissues and normal adjacent tissue (NAT) with the RNAscope in situ hybridization-based staining of ELFN1-AS1 (scale bar 50 µm), and (**c**) the quantification of the RNAscope-based ELFN1-AS1 expression in tumors and NATs (paired samples n = 32) with H-Score. Data are shown as mean + /−standard deviation (SD). *P*-value was determined by a paired two-tailed Student’s *t*-test. **d**, The endogenous expression of SMIMP was determined in the indicated CRC cancer cell lines that were transduced with the negative control sgRNA (sgNC) or sgRNAs targeting SMIMP (sgSMIMP), where β-tubulin was used as a loading control. Western blot data are representative of at least three independent experiments. **e-j**, The growth of the (**e**) CRL-1831, (**f**) HT-29, (**g**) SW-480, (**h**) RKO, (**i**) LoVo or (**j**). Caco-2 cells transduced with sgNC or sgSMIMP was monitored with CCK-8 assay. The OD450 absorbance for WST-8 formazan was measured each day for the indicated days. Data in **a** (n = 3), **e**(n = 5), **f**(n = 3), **g**(n = 4), **h**(n = 4), **i**(n = 4) and **j**(n = 4) are shown as mean + /−standard deviation (SD). *P*-values were determined by an unpaired two-tailed Student’s *t*-test. Source data

We further performed quantitative PCR with reverse transcription (RT–qPCR) and western blotting in CRC and immortalized colon cell lines to determine RNA and protein expression of SMIMP in these cell lines. In line with our finding that *ELFN1*-*AS1* expression is much higher in CRC tumor tissues than in normal colon tissues, SMIMP showed increased RNA (Extended Data Fig. 4a) and protein (Fig. 3g) expression in CRC cell lines compared to the immortalized colon cell line. RNAscope in situ hybridization analysis revealed that ELFN1-AS1 was upregulated in CRC tumor tissues compared with the normal tissues adjacent to the tumor (Extended Data Fig. 4b,c and the Methods). Western blot analysis of freshly frozen CRC tumors and matched normal tissues confirmed that SMIMP was upregulated at the protein level in CRC tumors (Fig. 3h and Supplementary Table 3). Aside from the HCT-116 and DLD-1 lines, we found that sgRNA-mediated depletion of SMIMP (Extended Data Fig. 4d) inhibited the growth of cancer cell lines with higher SMIMP protein expression, including the HT-29, SW-480 and RKO lines (Fig. 3g and Extended Data Fig. 4e–j). By contrast, SMIMP depletion did not affect the growth of cell lines with lower or undetectable SMIMP protein expression, including the LoVo, Caco-2 and CRL-1831 lines (Fig. 3g and Extended Data Fig. 4e–j). These results indicate that the differential functional dependencies of CRC cells on SMIMP are associated with its expression level.

### SMIMP exerts a tumor-promoting function in vitro and in vivo

**Fig. 4:**
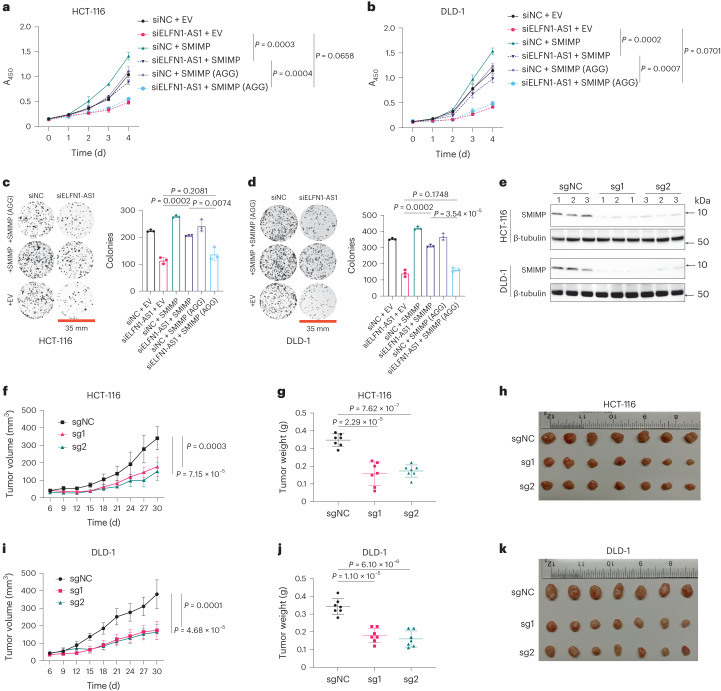
SMIMP exerts a tumor-promoting function. **a**,**b**, Rescue experiments for the cell growth defect caused by siRNA-mediated ELFN1-AS1 depletion in HCT-116 (**a**) and DLD-1 (**b**) cells. HCT-116/DLD-1 cells stably transduced to express SMIMP with a wild-type (ATG) or mutant (AGG) start codon or transduced with the empty vector (EV) control were transfected with negative-control siRNA (siNC) or siRNA species targeting ELFN1-AS1 (siELFN1-AS1) outside the CDS region and were cultured for 4 d. Cell growth was monitored each day with the CCK-8 assay. **c**,**d**, Rescue experiments for the clonogenic growth defect caused by siRNA-mediated ELFN1-AS1 depletion in HCT-116 (**c**) and DLD-1 (**d**) cells. Representative pictures of clonogenic growth and bar graphs quantifying the colonies formed by HCT-116 and DLD-1 cells transduced to express SMIMP with a wild-type or mutant (AGG) start codon or transduced with the EV control that were transfected with siNC or siELFN1-AS1. **e**, Endogenous expression of SMIMP in xenograft tumors derived from HCT-116 and DLD-1 cells transduced with negative-control sgRNA (sgNC) or SMIMP-targeting sgRNA species (sg1 and sg2) was determined by western blot. **f**, Volumes of the xenograft tumors derived from HCT-116 cells stably expressing the indicated sgRNA species (*n* = 7 for each group) were monitored every 3 d for a total of 30 d. Tumor volumes were calculated as indicated in the Methods. **g**,**h**, On day 30, the tumors were removed. Tumor weights (**g**) were measured, and images (**h**) were obtained. **i**–**k**, Volumes (**i**), weights (**j**) and images (**k**) were similarly obtained or measured for the xenograft tumors derived from DLD-1 cells stably expressing the indicated sgRNA species (*n* = 7 for each group). Except for the xenograft experiments (*n* = 7), when applicable, data are shown as mean ± s.d. (*n* = 3). *P* values were determined by an unpaired two-tailed Student’s *t*-test. Source data

**Extended Data Fig. 5:**
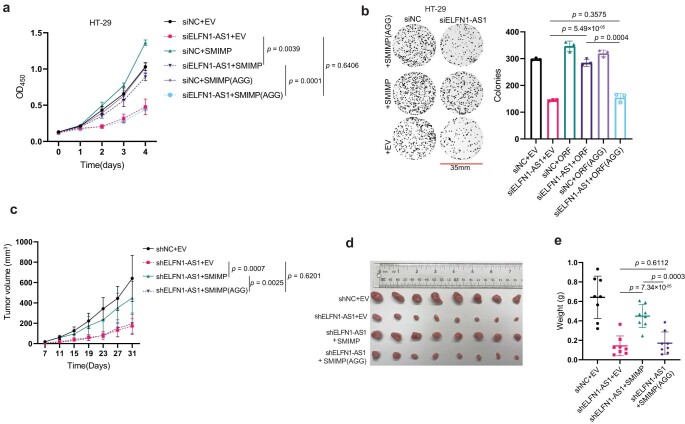
A tumor-promoting role of SMIMP in vitro and in vivo. **a**, The rescue experiments for the cell growth defect caused by siRNA-mediated ELFN1-AS1 depletion in HT-29 cells. The HT-29 cells stably expressing SMIMP that has a wild-type (ATG)/mutant (AGG) start codon or the empty vector control (EV), were transfected with the negative control siRNA (siNC) or siRNAs targeting ELFN1-AS1 (siELFN1-AS1) and were cultured for 4 days. The cell growth was monitored each day with CCK-8 assay. **b**, The rescue experiments for the clonogenic growth defect caused by siRNA-mediated ELFN1-AS1 depletion in HT-29 cells. The representative pictures of clonogenic growth and the bar graph quantifying the colonies formed by the HT-29 cells expressing SMIMP that has a wild- type/mutant (AGG) start codon or EV, were transfected with siNC or siELFN1-AS1. Pictures of clonogenic growth are representative of at least three independent experiments. Data in a and **b** are shown as mean + /−standard deviation (SD), n = 3. *P*-values were determined by an unpaired two-tailed Student’s *t*-test. **c**, The volumes of the xenograft tumors derived from HCT-116 cells stably transduced with the indicated shRNAs and expression vectors (n = 8 for each group), were monitored every 4 days for a total of 31 days. The tumor volumes were calculated as indicated in the Methods. **d**, **e**, On day 31, the tumors were removed. Their images (**d**) were collected and their weights (**e**) were measured. Data in **c** and **e** are shown as mean + /−standard deviation (SD), n = 8. *P*-values were determined by an unpaired two-tailed Student’s *t*-test. Source data

To validate the tumor-promoting function of SMIMP with a complementary approach to the CRISPR–Cas9 method, we performed small interfering RNA (siRNA)-mediated knockdown of ELFN1-AS1 by designing two siRNA species targeting the regions outside its CDS. Effective siRNA-mediated depletion of ELFN1-AS1 (Supplementary Fig. 2a–c) inhibited CRC cell growth (Supplementary Fig. 2d–f) and impaired CRC cell clonogenic capacity (Supplementary Fig. 2g–i). Overexpression of wild-type SMIMP rescued the loss-of-function phenotype caused by siRNA-mediated ELFN1-AS1 knockdown. By contrast, overexpression of mutant SMIMP with an ATG-to-AGG start codon mutation failed to do so (Fig. 4a–d and Extended Data Fig. 5a,b). These results further supported the growth-promoting function of SMIMP in vitro. To validate the observed loss-of-function phenotype in vivo, we subcutaneously injected HCT-116 and DLD-1 cells stably expressing either the sgRNA targeting SMIMP or a negative-control non-targeting sgRNA into the flank of nude mice (Methods). Effective sgRNA-mediated knockout of SMIMP (Fig. 4e) led to a significant decrease in xenograft tumor volume and weight in comparison with the negative control (Fig. 4f–k). Furthermore, overexpressing the wild-type SMIMP, but not the mutant with an ATG-to-AGG start codon mutation, largely rescued the loss-of-function phenotype caused by short hairpin RNA (shRNA)-mediated ELFN1-AS1 depletion in vivo (Extended Data Fig. 5c–e). Collectively, these findings indicate that SMIMP exerts a tumor-promoting function in vitro and in vivo.

### SMIMP interacts with SMC1A

**Fig. 5:**
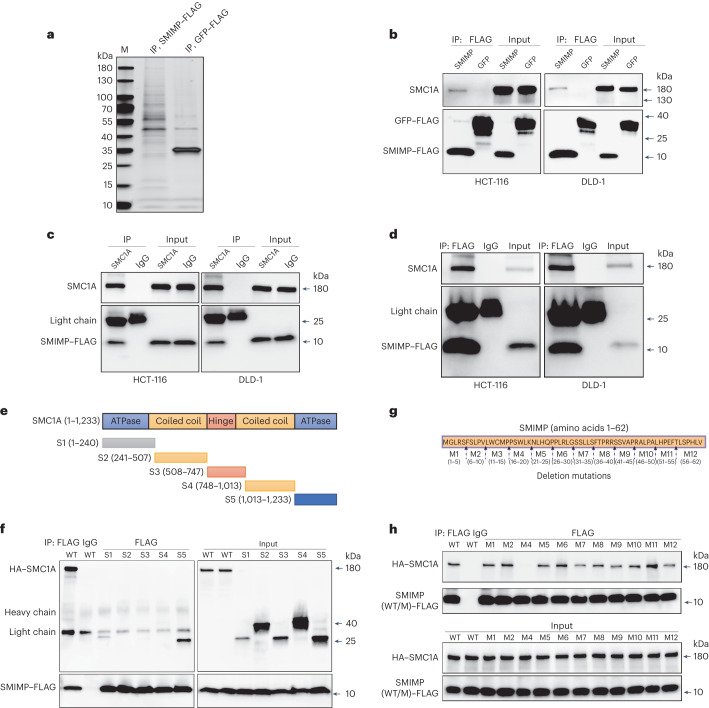
SMIMP interacts with SMC1A. **a**, The proteins interacting with SMIMP were identified using AP–MS. Silver staining showing proteins enriched by co-IP of FLAG-tagged SMIMP (SMIMP–FLAG) compared with the negative control of FLAG-tagged GFP (GFP–FLAG) in HCT-116 cells stably expressing SMIMP–FLAG or GFP–FLAG, respectively. Lane M represents molecular weight marker. **b**, Whole-cell lysates of HCT-116 and DLD-1 cells stably expressing SMIMP–FLAG or the negative control GFP–FLAG were immunoprecipitated with an anti-FLAG antibody. Co-immunoprecipitated SMC1A was then detected with an anti-SMC1A antibody. **c**, Whole-cell lysates of HCT-116 and DLD-1 cells stably expressing SMIMP–FLAG were immunoprecipitated with an anti-SMC1A antibody; mouse immunoglobulin G (IgG) was used as a negative control. Co-immunoprecipitated SMIMP–FLAG was then detected with an anti-FLAG antibody. **d**, Chromatin-bound protein extracts of HCT-116 and DLD-1 cells stably expressing SMIMP–FLAG were immunoprecipitated with an anti-FLAG antibody. Co-immunoprecipitated SMC1A was then detected with an anti-SMC1A antibody. **e**, Diagram illustrating different domains of full-length SMC1A and a series of truncation mutants generated based on this diagram (S1–S5). **f**, DNA for hemagglutinin (HA)-tagged wild-type (WT) SMC1A or individual truncation mutants was cotransfected with that for FLAG-tagged SMIMP into HEK293FT cells. Cell lysates were immunoprecipitated with an anti-FLAG antibody and then subjected to immunoblotting analysis. **g**, Diagram illustrating full-length SMIMP and a series of deletion mutants generated based on this diagram (M1–M12). **h**, DNA for FLAG-tagged wild-type SMIMP or individual deletion mutants was cotransfected with that for HA-tagged SMC1A into HEK293FT cells. Cell lysates were immunoprecipitated with an anti-FLAG antibody and then subjected to immunoblotting analysis. Western blot data are representative of at least three independent experiments. Source data

**Extended Data Fig. 6:**
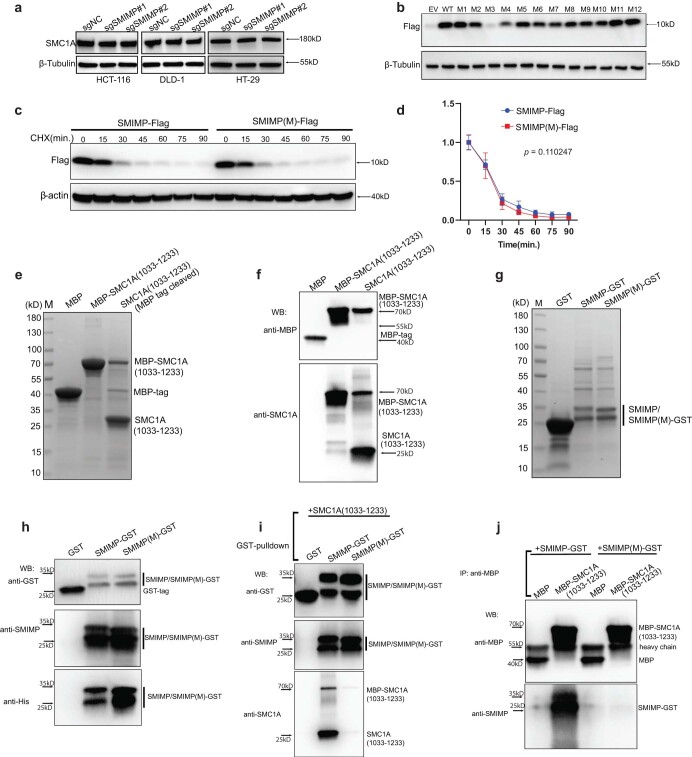
Characterization of SMIMP-SMC1A interaction. **a**, SMC1A expression was detected by western blot in the indicated CRC cancer cell lines that were transduced with the negative control sgRNA (sgNC) or SMIMP-targeting sgRNAs (sgSMIMP). **b**, The ectopically expressed FLAG-tagged wild-type SMIMP or individual deletion mutants (M1-M12) in HEK293FT cells was detected by western blot. **c**, The HCT-116 cells were treated with cycloheximide (CHX) at a final concentration of 50 ug/mL for the indicated time intervals, followed by detecting the expression of FLAG-tagged wild-type SMIMP/mutant SMIMP (M) (Del-M4) by western blot. **d**, The western blotting band intensity of FLAG-tagged SMIMP/SMIMP(M) protein was quantified by densitometry and normalized to β-actin control. The ratios between the normalized intensities at different time points with respect to the time zero were plotted. Data are shown as mean + /−standard deviation (SD), n = 3. *P*-values were determined by an unpaired two-tailed Student’s *t*-test. **e**, **f**, The His6-MBP-TEVsite-SMC1A1013-1233-StrepII and His6-SMIMP/SMIMP(M)-GST were expressed and purified in E. coli (Methods). The purified His6-MBP-TEVsite-SMC1A1013-1233-StrepII (MBP-SMC1A (1033-1233)) and the SMC1A1013-1233-StrepII (SMC1A (1033-1233)) was detected by (**e**) coomassie staining and by (**f**) western blot with an anti-MBP or anti-SMC1A antibody, where the purified MBP tag was used as a control. **g**, **h**, The purified His6-SMIMP/SMIMP(M)-GST (SMIMP-GST/SMIMP(M)-GST) was detected by (**g**) coomassie staining and by (**h**) western blot with an anti-GST, anti-SMIMP or anti-His antibody, where the purified GST tag was used as a control. **i**, In vitro GST pull-down experiments (Methods) for detecting the interaction between SMIMP-GST/SMIMP(M)-GST and SMC1A (1033-1233), where the purified recombinant GST served as a negative control. **j**, In vitro co-IP experiments with anti-MBP antibody (Methods) for detecting the interaction between MBP-SMC1A (1033-1233) and the SMIMP-GST/SMIMP(M)-GST, where the purified MBP served as a negative ontrol. Western blot data are representative of at least three independent experiments. Source data

To determine the functional mechanism for SMIMP, we performed affinity purification using an anti-FLAG antibody followed by MS (AP–MS) in HCT-116 cells stably expressing FLAG-tagged SMIMP, the ORF of AC012363.4 expressed with FLAG tag or FLAG-tagged green fluorescent protein (GFP) (Fig. 5a). We identified 22 proteins not in samples from the ORF of AC012363.4 expressed with FLAG tag or the GFP negative control (that is, with zero unique peptides; Methods) and showing significantly increased expression in COAD compared to normal colon tissue (log_2_ (fold change) ≥ 0.4, FDR < 0.01). These are potential candidates that may function together with SMIMP. Most of the proteins with strong supporting evidence based on MS data (≥4 detected unique peptides) are nuclear proteins (Supplementary Table 4). Among these nuclear proteins, we found an interesting candidate, SMC1A, that has a well-established function and mechanism and has a known tumor-promoting role in COAD^30^. SMC1A is the core subunit of the mitotic cohesion complex, a complex that is highly conserved in eukaryotes^31^. The mitotic cohesin complex or SMC1A plays diverse functions in regulating chromosome dynamics during the cell cycle and gene expression^32–35^. Reciprocal co-IP of SMC1A and FLAG-tagged SMIMP in CRC cells confirmed their interaction (Fig. 5b,c). Co-IP on the chromatin fractions also confirmed the SMIMP–SMCA1 interaction on chromatin (Fig. 5d). Importantly, sgRNA-mediated depletion of SMIMP did not affect the protein level of SMC1A (Extended Data Fig. 6a).

The SMC1A protein folds around a central globular hinge domain, with an N-terminal ATP-binding domain and a C-terminal ATP-hydrolysis domain that are connected by anti-parallel coiled coils^36^. The N- and C-terminal domains form an ATPase that is important for modulating binding of cohesin to DNA and cohesin-mediated DNA tethering^37,38^. To determine the regions in SMC1A important for mediating binding to SMIMP, we generated a series of SMC1A-truncation mutants (S1, amino acids 1–240; S2, amino acids 241–507; S3, amino acids 508–747; S4, amino acids 748–1,013; and S5, amino acids 1,013–1,233) based on its domain structure (Fig. 5e). We found that the ATPase-forming N-terminal (S1, amino acids 1–240) and C-terminal (S5, amino acids 1,013–1,233) domains of SMC1A are the two domains that form an interaction with SMIMP (Fig. 5f).

We next constructed a series of deletion mutants with the removal of every five- or seven-amino-acid fragment (M1–M12) along the full-length SMIMP in each mutant (Fig. 5g). Among the FLAG-tagged deletion mutants of SMIMP that were well expressed (except the M3 fragment deletion; Extended Data Fig. 6b), deletion of the M4 fragment (amino acids 16–20) abolished the binding of SMIMP to SMC1A (Fig. 5h), indicating that this region is essential to the SMIMP–SMC1A interaction. We performed the cycloheximide chase assay to compare the difference in stability between FLAG-tagged wild type and the M4 deletion mutant SMIMP. No significant difference in protein stability between FLAG-tagged wild type and the deletion mutant SMIMP was observed (Extended Data Fig. 6c,d), ruling out the potential confounding effect of deletion mutation on SMIMP stability that may result in a seemingly loss of interaction with SMC1A. To determine whether the interaction between SMIMP and SMC1A is direct, we expressed and purified the N- and C-terminal domains of SMC1A as well as wild-type and mutant SMIMP from *Escherichia coli* (Methods). Although we failed to obtain the soluble N-terminal domain, we managed to purify the soluble C-terminal domain of SMC1A along with wild-type and mutant SMIMP (Extended Data Fig. 6e–h). Through in vitro glutathione *S*-transferase (GST) pulldown and co-IP experiments (Methods), we found that the wild type but not the deletion mutant SMIMP showed an interaction with the recombinant C-terminal domain of SMC1A in vitro (Extended Data Fig. 6i,j), supporting the idea that the interaction between SMIMP and the SMC1A C-terminal domain is direct and that the M4 deletion mutation results in the loss of this direct interaction.

### SMC1A is important for mediating SMIMP function

**Fig. 6:**
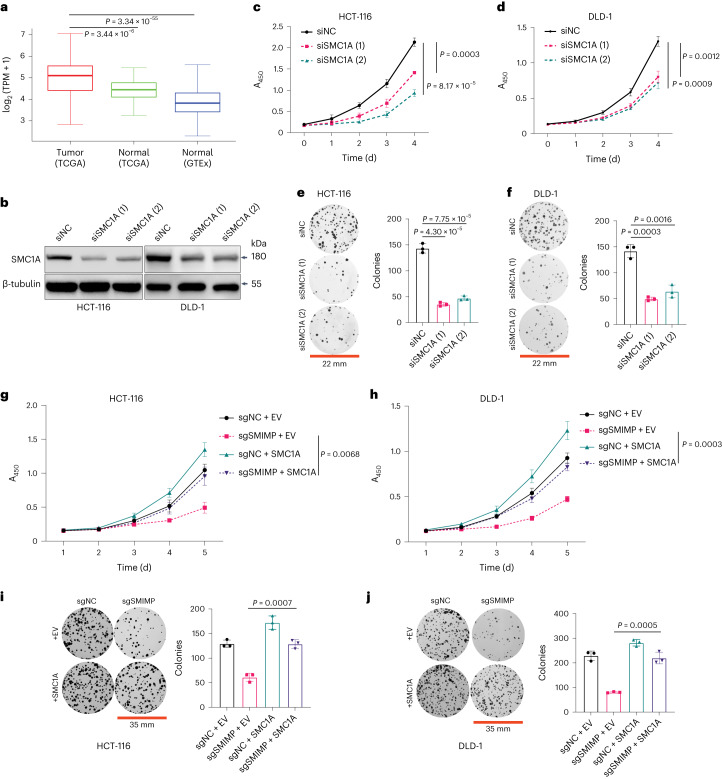
SMC1A is important for mediating SMIMP function. **a**, Box plot showing significantly elevated expression of SMC1A in CRC adenocarcinoma compared with the corresponding normal tissues based on TCGA-COAD (tumors, *n* = 290; normal tissues, *n* = 41) and GTEx (*n* = 287) RNA-seq data. The bottom and top edges of the box represent the first and third quartiles. The median is indicated by the line dividing the box into two parts. The whiskers illustrate the range between the bottom 5% and 25% as well as the top 25% and 5%. Outliers are shown as points. *P* values were determined by an unpaired two-sample Wilcoxon test. **b**, SMC1A protein levels in HCT-116 and DLD-1 cells transfected with negative-control siRNA (siNC) or SMC1A-targeting siRNA species (siSMC1A) were determined by western blot. **c**,**d**, Growth of HCT-116 (**c**) and DLD-1 (**d**) cells transfected with siNC or siSMC1A was monitored with the CCK-8 assay. **e**,**f**, Representative pictures of clonogenic growth and bar graphs quantifying the colonies formed by HCT-116 (**e**) and DLD-1 (**f**) cells transfected with the siNC or siSMC1A. **g**–**j**, The rescue effect of ectopic expression of SMC1A or the empty vector (EV) control on the growth defect (**g**,**h**) or the colony-formation defect (**i**,**j**) caused by sgRNA-mediated SMIMP depletion in HCT-116 and DLD-1 cells. HCT-116 and DLD-1 cells with the EV control or stably expressing SMC1A were transduced with the negative-control sgRNA (sgNC) or individual SMIMP-targeting sgRNA (sgSMIMP). Cell growth was monitored with the CCK-8 assay. Representative pictures of clonogenic growth and bar graphs quantifying the colonies formed by these cells are shown. Western blot data and pictures of clonogenic growth are representative of at least three independent experiments. When applicable, data are shown as mean ± s.d. (*n* = 3). *P* values were determined by an unpaired two-tailed Student’s *t*-test. Source data

Consistent with the reported tumor-promoting role of SMC1A^30^ and elevated expression of *SMC1A* in COAD tumor tissues compared with normal colon tissues (Fig. 6a), we confirmed that siRNA-mediated silencing of SMC1A (Fig. 6b) suppressed CRC cell growth and colony formation (Fig. 6c–f). We note that *SMC1A* is a common essential gene across many cancer cell lines based on CRISPR–Cas9 screens^24^.

**Extended Data Fig. 7:**
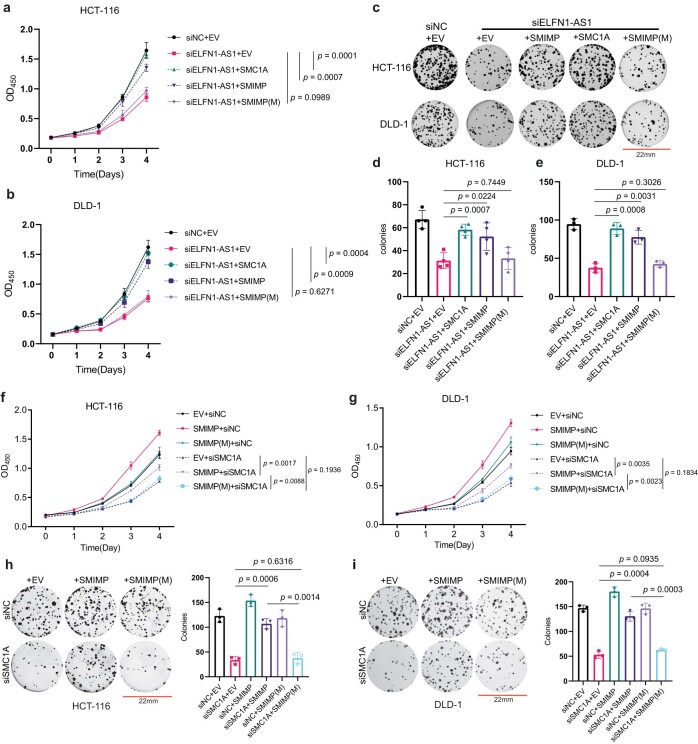
SMIMP-SMC1A interaction mediates SMIMP function. **a**, **b**, The (**a**) HCT-116 or (**b**) DLD-1 cells stably transduced with SMIMP/SMIMP(M), *SMC1A* or the empty vector control (EV), were transfected with siNC or siRNAs targeting ELFN1-AS1 outside the SMIMP-encoding CDS region (siELFN1-AS1) and were cultured for 4 days. The cell growth was monitored each day with CCK-8 assay. **c-e**, The (**c**) representative pictures of clonogenic growth and the (**d, e**) bar graph quantifying the colonies formed by the HCT-116/DLD-1 cells stably transduced with SMIMP/SMIMP(M), *SMC1A* or the EV, were transfected with siNC or siELFN1-AS1, after cells were cultured for two weeks. **f-i**, The effect of overexpressing the wild-type SMIMP or the M4 deletion mutant SMIMP (M) (Del-M4) that loses interaction with SMC1A on the (**f**, **g**) growth or (**h**, **i**) colony formation of HCT-116 and DLD-1 cells in the presence or absence of siRNA-mediated *SMC1A* knockdown. The HCT-116/DLD-1 cells stably expressing EV or the indicated ORFs were transfected with the negative control siRNA (siNC) or individual siRNA targeting *SMC1A* (siSMC1A). The cell growth was monitored with CCK-8 assay. The representative pictures of clonogenic growth and the bar graph quantifying the colonies formed by these cells are shown. Pictures of clonogenic growth are representative of at least three independent experiments. Data in **a-b** (n-3), **e-i** (n = 3) and **d** (n = 4) are shown as mean + /−standard deviation (SD). *P*-values were determined by an unpaired two-tailed Student’s *t*-test. Source data

To determine the role of SMC1A in mediating SMIMP function, we investigated whether the inhibition of CRC cell growth and colony formation caused by sgRNA-mediated SMIMP depletion could be rescued by overexpressing *SMC1A*. We found that overexpressing *SMC1A* rescued the sgRNA-mediated loss-of-function phenotype of SMIMP as well as reversed the effect of siRNA-mediated ELFN1-AS1 depletion on cell growth and colony formation (Fig. 6g–j and Extended Data Fig. 7a–e), indicating that SMC1A is important for mediating the growth-promoting function of SMIMP. Importantly, overexpressing wild-type SMIMP, but not the mutant SMIMP (deletion M4) showing defective SMIMP–SMC1A interaction, rescued the phenotype caused by siRNA-mediated ELFN1-AS1 depletion on cell growth and colony formation (Extended Data Fig. 7a–e), indicating a critical role of SMIMP–SMC1A interaction in mediating SMIMP function. Additionally, overexpressing the wild type, but not the M4 deletion mutant SMIMP, mitigated the loss-of-function phenotype of SMC1A on cell growth and colony formation (Extended Data Fig. 7f–i), suggesting a role of SMIMP–SMC1A interaction in modulating the tumor-promoting properties of SMC1A. Collectively, our data indicate that SMC1A is important for mediating the growth-promoting function of SMIMP through SMIMP–SMC1A interaction.

### SMIMP and SMC1A repress expression of tumor-suppressive genes

**Fig. 7:**
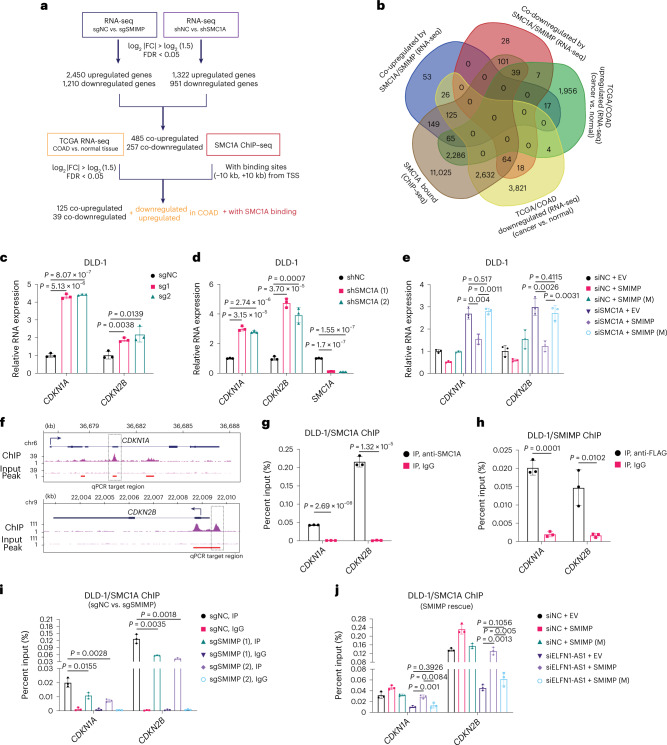
SMIMP and SMC1A repress tumor-suppressive gene expression. **a**, Workflow for identifying the protein-coding genes co-regulated by SMIMP and SMC1A that were potentially important for mediating the function of the SMIMP–SMC1A axis in CRC. FC, fold change. **b**, Venn diagrams showing overlaps between the protein-coding genes co-regulated by SMIMP and SMC1A based on RNA-seq data, the genes with at least one SMC1A ChIP–seq peak near their TSS (±10 kb) and the genes differentially expressed between cancer and normal tissues based on TCGA-COAD RNA-seq data. **c**,**d**, RT–qPCR analysis of *CDKN1A* and *CDKN2B* expression in DLD-1 cells following sgRNA-mediated SMIMP knockout (**c**) or shRNA-mediated SMC1A depletion (**d**). **e**, Rescue of *CDKN1A* and *CDKN2B* expression following SMC1A knockdown by ectopically expressing wild-type SMIMP or deletion mutant SMIMP (M) (deletion M4) with respect to the empty vector (EV) control. **f**, Visualization of the SMC1A ChIP–seq signal and peaks includes the signal track of SMC1A ChIP–seq (ChIP), the corresponding input (input) and significant peaks (peak). chr, chromosome. **g**, ChIP–qPCR analysis was performed with anti-SMC1A or anti-IgG antibodies in DLD-1 cells to validate the binding of SMC1A to the ChIP–seq peaks. **h**, ChIP–qPCR analysis was performed with anti-FLAG or anti-IgG antibodies in DLD-1 cells stably expressing FLAG-tagged SMIMP to examine the binding of SMIMP to the SMC1A ChIP–seq peaks. **i**, The occupancy difference of SMC1A on its ChIP–seq peaks was assessed by ChIP–qPCR analysis between DLD-1 cells transduced with SMIMP-targeting sgRNA species (sgSMIMP) and DLD-1 cells transduced with the negative-control sgRNA (sgNC). **j**, Upon ELFN1-AS1 knockdown, ChIP–qPCR analysis was performed to assess the rescue effect of ectopic expression of wild-type SMIMP or mutant SMIMP (deletion M4), with respect to the EV control, on SMC1A binding to *cis*-regulatory elements. When applicable, data are shown as mean ± s.d. (*n* = 3). *P* values were determined by an unpaired two-tailed Student’s *t*-test. Source data

**Extended Data Fig. 8:**
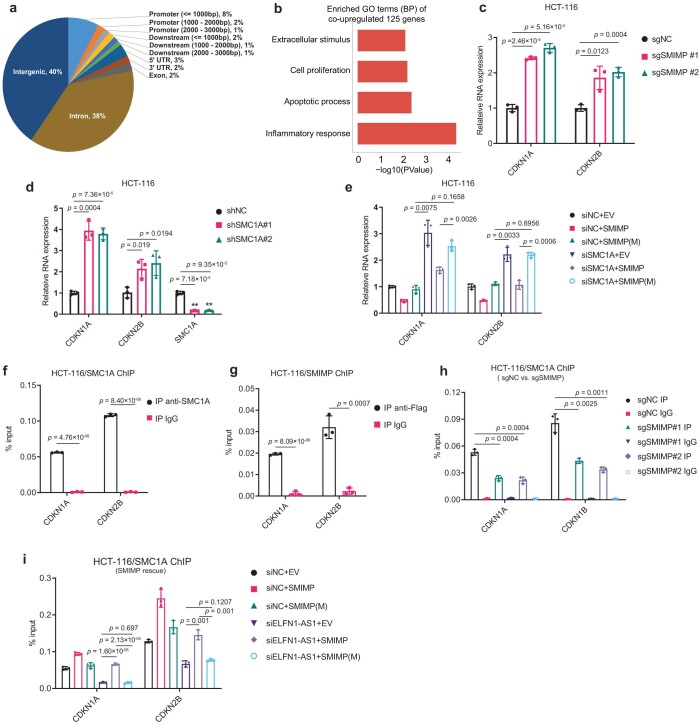
SMIMP/SMC1A inhibits the expression of CDKN1A and CDKN2B. **a**, The genome-wide distribution of SMC1A ChIP-seq peaks over different types of genomic regions. **b**, The top enriched GO biological processes of the 125 protein-coding genes with at least one SMC1A binding site within 10 kb from their transcription start sites, co-repressed by SMIMP/SMC1A and significantly down-regulated in COAD compared with normal colon tissues. **c**, qRT-qPCR analysis of *CDKN1A*/ *CDKN2B* expression in HCT-116 cells that were transduced with sgRNAs targeting SMIMP/the negative control (sgNC). **d**, qRT-qPCR analysis *CDKN1A*/*CDKN2B*/*SMC1A* expression in HCT-116 cells that were transduced with *SMC1A*-targeting shRNAs or the negative control shNC. **e**, In the presence of *SMC1A* knockdown, the rescue effect of ectopic expression of wild-type SMIMP or deletion mutant SMIMP (M) with respect to the empty vector control (EV), on *CDKN1A*/*CDKN2B* expression, was assessed by qRT-PCR analysis in HCT-116 cells. **f**, ChIP-qPCR analysis was performed with anti-SMC1A/anti-IgG in HCT-116 cells to validate the binding of SMC1A to the ChIP-seq peaks. **g**, ChIP-qPCR analysis was performed with anti-FLAG/anti-IgG in HCT-116 cells stably expressing the FLAG-tagged SMIMP to examine the binding of SMIMP to the SMC1A ChIP-seq peaks. **h**, The SMC1A occupancy difference on its ChIP-seq peaks associated with *CDKN1A* and *CDKN2B* was assessed by ChIP-qPCR analysis, between the HCT-116 cells transduced with sgRNAs targeting SMIMP and the ones transduced with the negative control sgNC. **i**, In the presence of ELFN1-AS1 knockdown, ChIP-PCR analysis was performed to assess the rescue effect of ectopic expression of wild-type SMIMP/mutant SMIMP (M) (Del-M4) with respect to the EV control, on the SMC1A binding to the cis-regulatory elements. When applicable, data are shown as mean + /−standard deviation (SD), n = 3. *P*-values were determined by an unpaired two-tailed Student’s *t*-test. Source data

In addition to its well-established function in chromosome segregation during the cell cycle, accumulating evidence indicates that the mitotic cohesin complex plays an important role in the control of gene expression^35^. Using the observation that sgRNA-mediated depletion of SMIMP did not affect the protein level of SMC1A (Extended Data Fig. 6a), we hypothesized that SMIMP and SMC1A may co-regulate expression of functionally important downstream targets. To test this hypothesis, we first performed RNA-seq to determine changes in gene expression upon sgRNA-mediated knockout of SMIMP or siRNA-mediated silencing of SMC1A. We identified 2,450 upregulated and 1,210 downregulated protein-coding genes (log_2_ |fold change| ≥ log_2_ (1.5) and FDR < 0.05) upon SMIMP knockout (Supplementary Table 5). SMC1A depletion resulted in 1,322 upregulated and 951 downregulated protein-coding genes (Supplementary Table 6). Consistent with our hypothesis that SMIMP and SMC1A may co-regulate target gene expression, a statistically significant (Fisher’s exact test, *P* < 2.2 × 10^−16^) number of upregulated (485) and downregulated (257) protein-coding genes were shared in response to SMIMP knockout and SMC1A knockdown (Fig. 7a). To identify the common targets of SMIMP and SMC1A that are important for mediating their tumor-promoting function, we generated SMC1A chromatin IP (ChIP) followed by sequencing (ChIP–seq) data (Supplementary Table 7) and performed an integrated analysis of RNA-seq, SMC1A ChIP–seq and TCGA data (Fig. 7a). We found that SMC1A dominantly bound to intergenic and intronic regions in the genome (Extended Data Fig. 8a). There were 435 common upregulated and 257 common downregulated protein-coding genes that harbored at least one SMC1A-binding site within 10 kb of their transcription start sites (TSSs) (Fig. 7a,b). Among the shared upregulated genes after SMIMP knockout and SMC1A knockdown, 125 were significantly downregulated in COAD compared with normal colon tissues. By contrast, among the shared downregulated genes, only 39 were significantly upregulated in COAD compared with normal colon tissues (Fig. 7b). Therefore, we focused on the 125 downstream targets (Supplementary Table 8), the expression of which was co-repressed by SMIMP and SMC1A and may play a tumor-suppressive role.

Gene ontology analysis of these 125 common downstream targets (Methods) revealed that the top enriched pathways or biological processes were the inflammatory response, regulation of cell proliferation, apoptotic process and extracellular stimuli (Extended Data Fig. 8b), which have an established role in tumor initiation and/or development. To understand the tumor cell-autonomous mechanism underlying the tumor-promoting function of SMIMP and SMC1A, we further investigated the two targets cyclin-dependent kinase inhibitor (CDKN)1A and CDKN2B that have an established role in cell proliferation. CDKN1A and CDKN2B exert an important tumor-suppressive function by regulating cell cycle progression^39,40^. Real-time RT–qPCR analysis confirmed that expression of *CDKN1A* and *CDKN2B* was upregulated upon SMIMP knockout or SMC1A knockdown in HCT-116 and DLD-1 cells (Fig. 7c,d and Extended Data Fig. 8c,d). Overexpression of wild-type SMIMP, but not the M4 deletion mutant SMIMP, partially reversed the upregulation of these genes caused by *SMC1A* knockdown in HCT-116 and DLD-1 cells (Fig. 7e and Extended Data Fig. 8e), indicating that the SMIMP–SMC1A interaction is important for mediating their co-repression of target expression.

### SMIMP facilitates SMC1A binding to *cis*-regulatory elements

**Extended Data Fig. 9:**
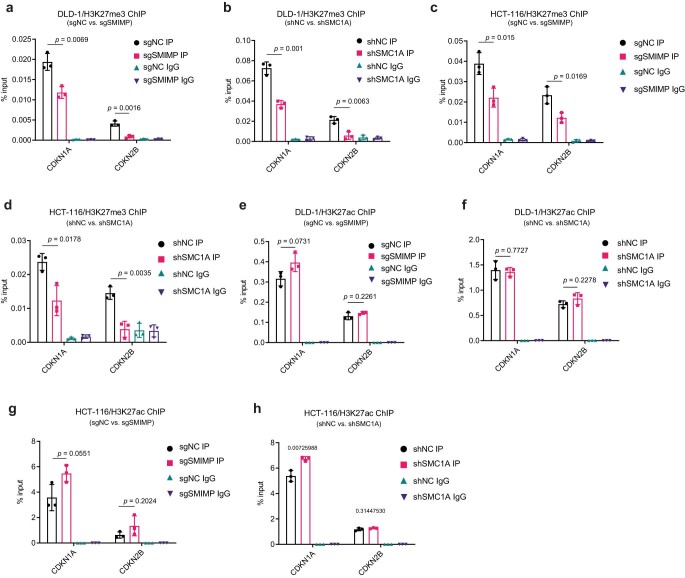
SMIMP/SMC1A promotes epigenetic repression. **a**, **b**, ChIP-qPCR analysis was performed in DLD-1 cells with an anti-H3K27me3/anti-IgG antibody to assess the effect of (**a**) sgRNA-mediated SMIMP knockout or (**b**) shRNA-mediated *SMC1A* knockdown on the H3K27me3 signal within the SMC1A binding sites associated with *CDKN1A* and *CDKN2B*. **c**, **d**, ChIP-qPCR analysis was performed in HCT-116 cells with an anti-H3K27me3/anti-IgG antibody to assess the effect of (**c**) sgRNA-mediated SMIMP knockout or (**d**) shRNA-mediated *SMC1A* knockdown on the H3K27me3 signal within the SMC1A binding sites around *CDKN1A* and *CDKN2B*. **e**, **f**, ChIP-qPCR analysis was performed in DLD-1 cells with an anti-H3K27ac/anti-IgG antibody to assess the effect of (**e**) sgRNA-mediated SMIMP knockout or (**f**) shRNA-mediated *SMC1A* knockdown on the H3K27ac signal within the SMC1A binding sites associated with *CDKN1A* and *CDKN2B*. **g**, **h**, ChIP-qPCR analysis was performed in HCT-116 cells with an anti-H3K27ac/anti-IgG antibody to assess the effect of (**g**) sgRNA-mediated SMIMP knockout or (**h**) shRNA-mediated *SMC1A* knockdown on the H3K27ac signal within the SMC1A binding sites associated with *CDKN1A* and *CDKN2B*. When applicable, data are shown as mean + /−standard deviation (SD), n = 3. *P*-values were determined by an unpaired two-tailed Student’s *t*-test. Source data

As SMIMP interacted with SMC1A on chromatin and did not regulate the protein level of SMC1A, we hypothesized that SMIMP may regulate the expression of SMIMP–SMC1A common targets by facilitating SMC1A binding to the *cis*-regulatory elements of these targets. To test this hypothesis, we evaluated the effect of SMIMP knockout on SMC1A binding to the *cis*-regulatory elements of *CDKN1A* and *CDKN2B* by ChIP followed by quantitative PCR (ChIP–qPCR). We found that SMIMP bound to the SMC1A-binding sites associated with *CDKN1A* and *CDKN2B* (Fig. 7f–h and Extended Data Fig. 8f,g), and SMC1A occupancy on these binding sites was significantly reduced upon SMIMP knockout (Fig. 7i and Extended Data Fig. 8h). Overexpression of wild-type SMIMP, but not the mutant (deletion M4), largely reversed the reduction of SMC1A binding to the *cis*-regulatory elements of *CDKN1A* and *CDKN2B* caused by siRNA-mediated ELFN1-AS1 depletion (Fig. 7j and Extended Data Fig. 8i). These results indicated that SMIMP–SMC1A interaction is critical for facilitating SMC1A binding to the *cis*-regulatory elements of common targets. Interestingly, SMIMP knockout or SMC1A knockdown significantly reduced the histone 3 lysine 27 trimethylation (H3K27me3) signal, a histone modification marking transcription repression at SMC1A-binding sites associated with *CDKN1A* and *CDKN2B* (Extended Data Fig. 9a–d) but not the histone 3 lysine 27 acetylation (H3K27ac) signal (Extended Data Fig. 9e–h), a histone modification marking active transcription. These findings indicated that SMIMP and SMC1A repressed common target expression by promoting epigenetic repression.

### A tumor-promoting role of SMIMP in non-CRC cancers

**Fig. 8:**
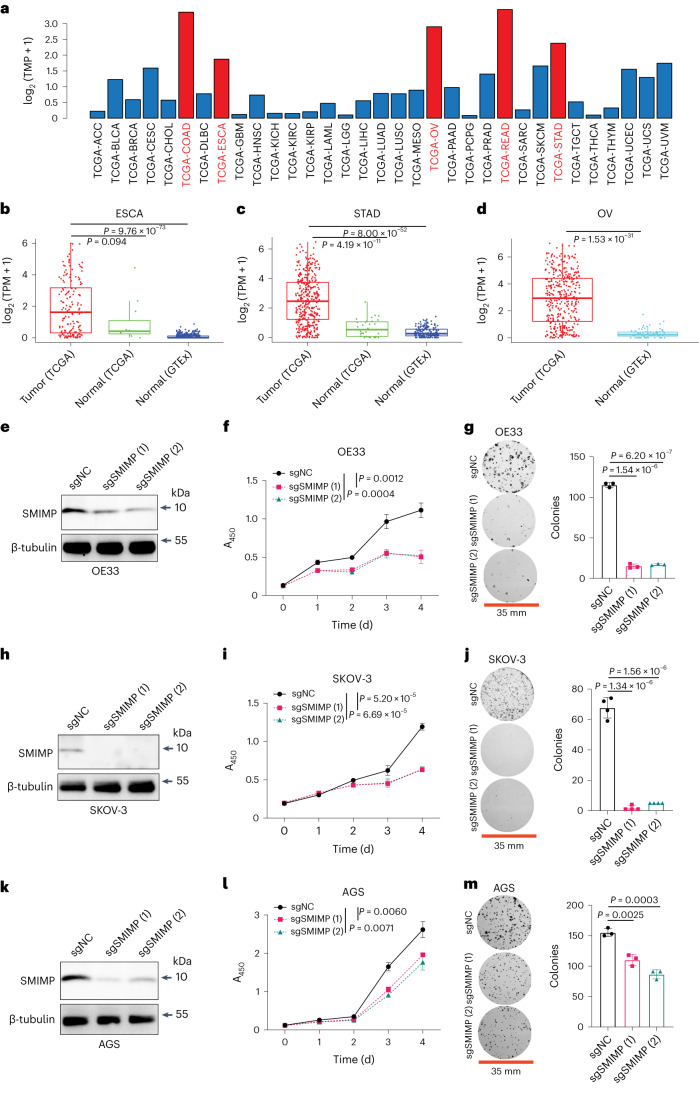
A tumor-promoting role of SMIMP in esophageal, gastric and ovarian cancer. **a**, Bar graph showing expression of *ELFN1*-*AS1* across 33 cancer types in TCGA. Adrenocortical carcinoma (ACC), bladder urothelial carcinoma (BLCA), breast invasive carcinoma (BRCA), cervical squamous cell carcinoma and endocervical adenocarcinoma (CESC), cholangiocarcinoma (CHOL), lymphoid neoplasm diffuse large B-cell lymphoma (DLBC), esophageal carcinoma (ESCA), glioblastoma multiforme (GBM), head and neck squamous cell carcinoma (HNSC), kidney chromophobe (KICH), kidney renal clear cell carcinoma (KIRC), kidney renal papillary cell carcinoma (KIRP), acute myeloid leukemia (LAML), brain lower grade glioma (LGG), liver hepatocellular carcinoma (LIHC), lung adenocarcinoma (LUAD), lung squamous cell carcinoma (LUSC), mesothelioma (MESO), ovarian serous cystadenocarcinoma (OV), pancreatic adenocarcinoma (PAAD), pheochromocytoma and paraganglioma (PCPG), prostate adenocarcinoma (PRAD), rectum adenocarcinoma (READ), sarcoma (SARC), skin cutaneous melanoma (SKCM), stomach adenocarcinoma (STAD), testicular germ cell tumors (TGCT), thymoma (THYM), thyroid carcinoma (THCA), uterine carcinosarcoma (UCS), uterine corpus endometrial carcinoma (UCEC), uveal melanoma (UVM). **b**–**d**, Box plots showing expression of *ELFN1*-*AS1* in ESCA (**b**) (tumors, *n* = 159; normal tissues, *n* = 11; GTEx samples, *n* = 591), STAD (**c**) (tumors, *n* = 373; normal tissues, *n* = 31; GTEx samples, *n* = 163) and OV (**d**) (tumors, *n* = 378; GTEx samples, *n* = 82) based on TCGA and GTEx RNA-seq data. The first and third quartiles are depicted by the bottom and top edges of the box, respectively. The median is indicated by the line that divides the box into two sections. Extending from the box, the whiskers illustrate the range between the bottom 5% and 25% as well as the top 25% and 5%. Any outliers are displayed as individual points. *P* values were determined by an unpaired two-sample Wilcoxon test. **e**–**m**, The effects of sgRNA-mediated knockout of SMIMP on SMIMP protein expression, the growth phenotype and the colony-forming capability were assessed for esophageal cancer OE33 cells (**e**–**g**), ovarian cancer SKOV-3 cells (**h**–**j**) and stomach adenocarcinoma AGS cells (**k**–**m**) that were transduced with individual sgRNA species targeting SMIMP or negative-control sgRNA. Western blot data and pictures of clonogenic growth are representative of at least three independent experiments. When applicable, data are shown as mean ± s.d. (*n* = 3). *P* values were determined by an unpaired two-tailed Student’s *t*-test. Source data

**Extended Data Fig. 10:**
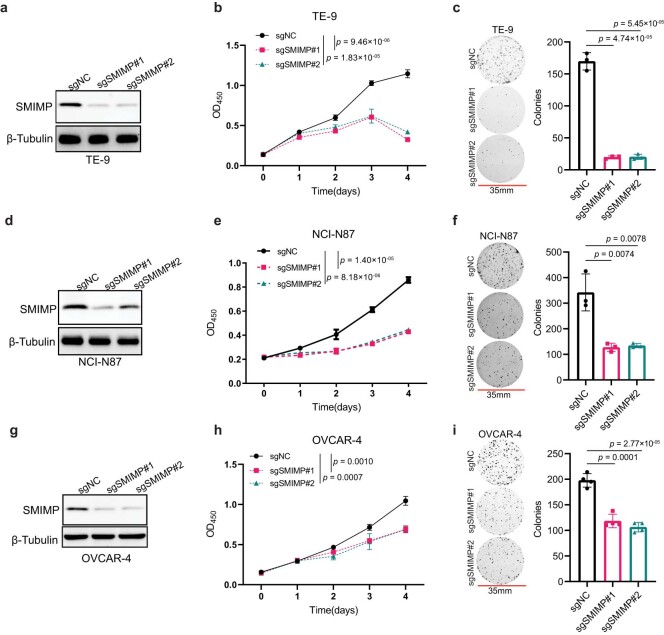
SMIMP exerts a growth-promoting function in esophageal, gastric, and ovarian cancer cells. The effects of sgRNA-mediated knockout of SMIMP on total SMIMP protein expression as well as on cell growth and colony-forming capability, were assessed in the ESCA TE9 cells (**a-c**), STAD NCI-N87 cells (**d-f**), and OV OVCAR-4 cells (**g-i**) that were transduced with individual sgRNAs targeting SMIMP (sgSMIMP) or the negative control sgRNA (sgNC). Western blot data and the pictures of clonogenic growth are representative of at least three independent experiments. Data in b, c, e, f, h (n = 3) and i (n = 4) are shown as mean + /−standard deviation (SD). *P*-values were determined by an unpaired two-tailed Student’s *t*-test. Source data

Through integrative analyses of TCGA data across 33 different tumor types, we found that *ELFN1*-*AS1*, the host lncRNA gene of SMIMP, exhibited a cancer-type specific expression pattern: *ELFN1*-*AS1* had much higher expression in CRC, READ, ovarian cancer, stomach adenocarcinoma and esophageal cancer but very low expression in adrenocortical carcinoma, glioblastoma, renal cancer, low-grade glioma, pheochromocytoma and paraganglioma, sarcoma and thyroid cancer (Fig. 8a). In addition, it showed significantly elevated expression in ovarian cancer, stomach adenocarcinoma and esophageal cancer compared with the corresponding normal tissues from GTEx or TCGA (except for esophageal cancer due to a small number of TCGA normal tissues) (Fig. 8b–d). To determine the role of SMIMP in these cancer types, we examined the CRISPR–Cas9-based loss-of-function phenotype of SMIMP on cell growth and colony formation in the corresponding cancer cell line models. Consistent with its higher expression in ovarian cancer, stomach adenocarcinoma and esophageal cancer, sgRNA-mediated knockout of SMIMP inhibited the growth of the cancer cell line models with good SMIMP expression corresponding to these cancer types and impaired their clonogenic capacity (Fig. 8e–m and Extended Data Fig. 10a–i).

## Discussion

Ribo-seq-based translatome profiling has revealed extensive translation of cryptic non-canonical ORFs within the regions of RNA species that are traditionally considered to be noncoding. A recent study by Chen et al.^14^ suggests that a substantial fraction of human non-canonical ORFs identified in normal human cells, including pluripotent stem cells (iPSCs), iPSC-derived cardiomyocytes and human foreskin fibroblasts, may encode functional proteins. Unlike the traditional view of uORFs as *cis*-acting translational control elements, they found that multiple uORF-encoded microproteins form stable complexes with the main protein encoded on the same mRNA, suggesting a *trans*-acting function of human uORFs. Previous studies have uncovered the tumor-suppressive function of microproteins in CRC^41,42^. By contrast, the current study aims for an unbiased discovery of cryptic ORF-encoded proteins (both microprotein and non-microprotein) that may exert a tumor-promoting function and might serve as potential therapeutic targets in CRC. Consistent with recent studies^15,43^ revealing functional non-canonical ORF-encoded proteins in cancer, we identified 25 non-canonical ORFs with higher RNA expression in COAD than in normal colon tissues. More than 60% of them showed higher expression in specific CRC molecular subtypes, suggesting their broad functional impact in different subtypes. The hits enriched in the CMS3 and CMS4 subtypes were underrepresented compared with the CMS1 and CMS2 subtypes, suggesting a potential discovery bias when a single cell line was used for the CRISPR–Cas9 screen. Extending the CRISPR–Cas9 screen of non-canonical ORFs to more cell lines representing diverse CRC subtypes is likely to enable more comprehensive discovery of functional ORFs enriched in different CRC subtypes. The SSC method^19^ used for sgRNA design optimizes on-target sgRNA activity but does not explicitly account for the sgRNA off-target effect. Therefore, it is important to combine RNA interference (RNAi)-mediated knockdown with ORF overexpression as an orthogonal approach to independently validate the function of the ORF hits identified from the CRISPR–Cas9 screen. Using an improved sgRNA design algorithm^44^ that accounts for minimizing sgRNA off-target effects may aid in reducing the false positives caused by potential off-target effects in CRISPR–Cas9 screens of non-canonical ORFs.

Microproteins encoded by non-canonical ORFs have been shown to play diverse functions in the cytoplasm, mitochondria and sarcoplasmic reticulum membrane; whereas our understanding of the chromatin function of human microproteins is rather limited. Emerging evidence suggests an important microprotein function in regulating transcription^15,45^ or the DNA damage pathway^46^. Our findings demonstrate a cryptic microprotein as an important component of cohesin-mediated gene regulation, underscoring the underappreciated role of microprotein in chromatin regulation. Different from the disruptive small proteins in plants that exert their function via dominant-negative suppression of transcription factor complex formation^47^, SMIMP exerts its function by enhancing the chromatin function of its interaction partner.

The mitotic cohesin complex is evolutionarily conserved and is composed of four subunits, including the SMC proteins SMC1A and SMC3, the subunit RAD21 and stromal antigen (SA; also known as STAG). Aside from the established function of the mitotic cohesin complex in mediating chromosome segregation during the cell cycle, increasing evidence indicates that it plays an important role in DNA replication and transcriptional regulation of gene expression. An emerging theme suggests that the common principle underlying the diverse function of the cohesin complex is its capability of binding to and tethering different genomic regions, requiring its constituent ATPases. Our finding that SMIMP interacted with the ATPase-forming N- and C-terminal domains of SMC1A suggests that SMIMP might regulate SMC1A chromatin binding by modulating its ATPase activity. Understanding the detailed mechanisms by which SMIMP–SMC1A interaction regulates SMC1A chromatin function will be an important next step. Given the key role of ATPase domains of cohesin in forming higher-order chromatin structure, our study also suggests that SMIMP might be involved in SMC1A-mediated high-order three-dimensional chromatin organization.

The SMIMP-encoding lncRNA ELFN1-AS1 is encoded by a primate-specific lncRNA gene that originated de novo throughout evolution, indicating that cancer cells not only upregulate and hijack evolutionarily conserved but also lineage-specific proteins that are expressed at low levels in normal tissues to promote their fitness. Past efforts of cancer therapeutic target and diagnostic biomarker discovery have been dominantly focused on proteins evolutionarily conserved between humans and mice. Our findings suggest that the proteins encoded by the cryptic non-canonical ORFs that are created through lineage-specific evolutionary changes may represent a new and untapped target-discovery space for developing cancer therapeutics and diagnostics.

Given the potential context-specific function of human non-canonical ORFs, we anticipate that our study only unraveled a small fraction of functional ORFs. Future large-scale studies like the current one promise to open new avenues for revealing the function of human non-canonical ORFs in cancer and other complex diseases.

## Methods

Animal studies were approved by the Institutional Animal Care and Use Committee of the UT MD Anderson Cancer Center (MDACC; IACUC study 00001077-RN02). No human research participants were involved in the current study. All freshly frozen tissue samples were purchased as de-identified tissues from US Biolab. The human paraffin-embedded tissue array was purchased as a de-identified tissue array from US Biomax.

### Cell culture, plasmids and antibodies

Human CRC cell lines HCT-116, DLD-1, HT-29, SW-480, RKO, LoVo and Caco-2 and the immortalized colon epithelial cell line CRL-1831 were obtained from the American Type Culture Collection and cultured according to instructions from the American Type Culture Collection. Human embryonic kidney cell lines HEK293T and HEK293FT were obtained from the Characterized Cell Line Core Facility at the MDACC and cultured in DMEM medium (Hyclone, SH30022.01). HCT-116 cells were cultured in McCoy’s 5a medium (Corning, 10-050-CV). DLD-1 and HT-29 cells were cultured in RPMI-1640 medium (Hyclone, SH30027.1). All culture media were supplemented with 10% FBS (Gibco, 10437-028) and 1% penicillin–streptomycin (Corning, 30-002-CI). All cell lines were cultured in an incubator (Thermo, Heracell VIOS 160i) with 5% CO_2_ at 37 °C. SMC1A (32363) and pLenti-CMV-Blast DEST (w118-1) (17452) expression plasmids were obtained from Addgene. The 5′ UTR of SMIMP (DNA), SMIMP (DNA), the 5′ UTR of the ORF of AC012363.4 and the ORF of AC012363.4 were amplified from cDNA extracted from HCT-116 cells and cloned into the pLenti-CMV-Blast DEST vector. The wild-type and mutants of SMIMP (DNA) were cloned into pLVX-puro with DNA for the FLAG tag. DNA for the wild-type and mutants of SMC1A was cloned into pcDNA3.1 with DNA for the HA tag. All plasmid sequences were confirmed by Sanger sequencing. The primary antibodies used in this study include monoclonal anti-FLAG M2 antibody (Sigma-Aldrich, F1804) (western blot, 1:5,000; IP, 5 µg; chromatin immunoprecipitation, 3 µg; immunofluorescence, 1:500), rabbit anti-SMC1 antibody (Bethyl, A300-055A) (western blot, 1:2,000; IP, 2–5 µg; ChIP, 5 µg) rabbit anti-β-tubulin (9F3) antibody (CST, 2128) (western blot, 1:1,000), rabbit anti-HA-tag antibody (C29F4) (CST, 3724) (western blot, 1:1,000; IP, 1:50), rabbit anti-EPB41L5 antibody (Invitrogen, PA5-5800) (western blot, 0.04–4 µg ml^−1^), rabbit anti-ELFN1 antibody (US Biological, 035032) (western blot, 1:1,000), rabbit anti-SMIMP polyclonal antibody (ABclonal) (western blot, 1:500), β-tubulin polyclonal antibody (Proteintech, 10068-1-AP) (1:2,000), mouse monoclonal anti-MBP-tag antibody (Proteintech, 66003-1-Ig) (western blot, 1:1,000–1:8,000; IP, 4 µg), rabbit polyclonal anti-GST-tag antibody (Proteintech, 10000-0-AP) (western blot, 1:1,000–1:4,000), mouse monoclonal anti-His-tag antibody (Proteintech, 66005-1-Ig) (western blot, 1:5,000). The secondary antibodies used in the study include goat anti-rabbit IgG, HRP-linked antibody (CST, 7074) (western blot, 1:3,000), horse anti-mouse IgG, HRP-linked antibody (CST, 7076) (western blot, 1:3,000) and goat anti-mouse IgG (H + L) highly cross-adsorbed secondary antibody, Alexa Fluor Plus 488 (Invitrogen, A32723) (immunofluorescence, 1:1,000).

### Ribosome profiling and library preparation

Sample preparation for ribosome profiling was similarly conducted as described previously^7,15^. In brief, HCT-116 cells were treated with cycloheximide (Sigma-Aldrich, final concentration of 0.1 mg ml^−1^) for 1 min, and the cells were lysed by using Mammalian Lysis Buffer (including cycloheximide at a concentration of 0.1 mg ml^−1^). Next, 600 µl of lysates were taken, 15 µl of RNase I (100 U µl^−1^, Thermo Fisher Scientific) was added, and the mixtures were incubated for 45 min at room temperature, followed by adding 15 µl SUPERase•In RNase inhibitor (Thermo Fisher Scientific) to stop the reaction. Ribosome recovery was carried out with illustra MicroSpin S-400 HR Columns (GE Healthcare), and RPFs were purified with an RNA Clean & Concentrator kit (Zymo Research). Ribosomal RNA was removed using the Ribo-Zero Magnetic Gold Kit (Human/Mouse/Rat, Illumina). RPFs without ribosomal RNA were run on a 15% urea denaturing PAGE gel, and gel slices corresponding to ~28–30 nucleotides were excised. RPF RNA was eluted and precipitated followed by library construction according to the manufacturer’s protocol.

### Ribo-seq data analysis and non-canonical ORF prediction

Cryptic non-canonical lncRNA-encoded ORFs with an ATG start codon were predicted based on the Ribo-TISH pipeline as described previously^7,15^ using in-house ribo-seq data from HCT-116 cells (GSE184322) and published ribo-seq data from HCT-116 (GSE58207)^48^, MCF-7 (GSE69923) and MDA-MB-231 cells (GSE77401). In brief, RPF reads were trimmed, and low-quality reads were filtered using Sickle (http://github.com/ucdavis-bioinformatics/sickle). After filtering, RPF reads were mapped to human rRNA sequences using Bowtie and allowing for two mismatches. The reads that were not mapped to human rRNA sequences were then mapped to the human genome (GRCh38) with transcriptome annotations from GENCODE version 22, NCBI RefSeq and MiTranscriptome^49^ and lincRNA transcript annotations generated by J. Rinn’s group^50^ using STAR version 2.6.1b^51^ with parameters ‘– outFilterMismatchNmax 2 –outFilterIntronMotifs RemoveNoncanonicalUnannotated –alignIntronMax 20000 –outMultimapperOrder Random –outSAMmultNmax 1 –alignEndsType EndToEnd’. Quality control was performed using the Ribo-TISH quality module with all uniquely mapped RPF reads in the annotated ORFs. RPFs were grouped by their lengths, and each aligned RPF read was represented by its 5′ end before estimation of the P-site offset. The metagene RPF count profile near the start and stop codons was constructed by summing the RPF count between −40 and +20 bp of the first base of the start and stop codons across all annotated protein-coding genes. The P-site offset was estimated based on the distribution of the 5′ end of the metagene RPF counts near the annotated start codons. The RPF count between 15 bp upstream of the first base of the start codon and 12 bp upstream of the first base of the stop codon were used to calculate RPF count distributions across three reading frames. The fraction of RPF counts in the dominant frame (*f*_d_) was calculated as the ratio between the maximum RPF count among all three reading frames and the sum of RPF counts from all reading frames. The cryptic non-canonical lncRNA-encoded ORFs with an ATG start codon were then identified using the Ribo-TISH predict module with regular ribo-seq data in the longest mode (*P* < 0.05). The same ORFs in different ribo-seq libraries and different transcript isoforms were merged.

### CRISPR–Cas9 sgRNA library design

sgRNA species targeting cryptic non-canonical ORFs were designed similarly as described previously^15,21,22^ using the SSC method^19^. The command line version of SSC (https://sourceforge.net/projects/spacerscoringcrispr/) was used for sgRNA design with default parameters optimized for CRISPR–Cas9 knockout in the human genome. SSC scans genomic sequences for CRISPR–Cas9 targets and prioritizes candidate sgRNA species based on their predicted efficiency. It implements an elastic net regression framework to build a predictive model of sgRNA efficiency from sgRNA sequences. SSC has been widely validated in different datasets and outperformed the other methods available at the time of its publication. Among the SSC-designed sgRNA species, we further filtered out sgRNA species that meet one of the following criteria: (1) mapping to multiple genomic regions, (2) with any Ns or more than three consecutive Ts, (3) with a high level of GC content (>60%) or (4) with guide efficiency score < 0.2. The 636 sgRNA species targeting 106 core essential genes were included as positive controls, and the 1,064 sgRNA species that target AAVS1 sites in the human genome or that do not target the human genome were included as negative controls.

### Single-guide RNA library construction

The sgRNA library was constructed as described previously^15,21,22^. sgRNA species flanked by linker sequences (sequences are in Supplementary Table 9) were synthesized as a pooled library using CustomArray 12K chips (CustomArray). The array-synthesized sgRNA library was amplified for eight cycles (primer sequences are in Supplementary Table 9) with Q5 High-Fidelity DNA Polymerase (New England Biolabs, M0491S). The PCR product was purified and assembled into a BsmBI (Thermo Fisher, ER0452)-digested lentiGuide-Puro vector (Addgene, 52963) by Gibson assembly (Gibson Assembly Master Mix, New England Biolabs, E2611L).

A total of 2 µl of 10–50 ng µl^−1^ ligation products was transfected into 25 µl electrocompetent cells (Lucigen) by using the MicroPulser Electroporator (Bio-Rad) with the one-shot EC1 program (approximately three to four reactions for one library). The transformed electrocompetent cells were plated on premade 24.5-cm^2^ bioassay plates (ampicillin) using a spreader after recovering in recovery medium for 1 h with rotation at 37 °C. All plates were grown inverted for 14 h at 32 °C. Finally, the colonies were scraped off, and the plasmids were extracted with the NucleoBond Xtra Midi EF kit (Takara, 740422.50) for downstream virus production.

### CRISPR–Cas9 screen and data analysis

The CRISPR–Cas9 screen was performed similarly as previously described^15,21,22^. To produce lentiviruses, HEK293FT cells were cotransfected with pCMV-VSV-G, psPAX2 and lentiCas9-GFP or the sgRNA library-expressing lentiGuide-Puro plasmid using jetPRIME (Polyplus-transfection, 114-15). Lentiviruses were collected 48 h after transfection and were then used to infect cell lines. HCT-116 cells transduced with lentiCas9-EGFP (Addgene, 63592) were sorted on a FACSAria cell sorter (BD Biosciences), and cells with high EGFP expression were collected. These HCT-116 cells with high SpCas9 expression were plated into ten 10-cm dishes and infected with lentiviruses containing the sgRNA library at an MOI of ~0.2–0.3. Following puromycin (2 µg ml^−1^) selection for 4 d, cells were collected as the starting pool for the screen. A total of 8 × 10^6^ cells per replicate (three replicates) from the starting pool were collected to extract genomic DNA for day 0 samples using the QIAamp DNA Mini Kit (QIAGEN). The rest of the cells were split into replicates (~500× coverage for each sgRNA per replicate) and were passed every 3 d and cultured for 21 d. On day 21, 8 × 10^6^ cells per replicate (six replicates) were collected to extract genomic DNA for the day 21 samples. Next-generation sequencing-ready sgRNA libraries were prepared with two rounds of PCR using the KAPA HiFi HotStart ReadyMix (Roche, KK2602). For each replicate at day 0 or day 21, 40 µg of input genomic DNA was extracted and used as the template in eight reactions (5 µg per reaction) to conduct the first-round PCR for 16 cycles. PCR products of different reactions were then pooled, and 20 µl of the mixed product was used as a template in one of two reactions for the second-round PCR. The second-round PCR was conducted for 12 cycles to incorporate Illumina barcode sequences (forward, AATGATACGGCGACCACCGAGATCTACAC<Illumina index eight-nucleotide barcode>ACACTCTTTCCCTACACGACGCTCTTCCGATCTTCTTGTGGAAAGGACGAAACACCG; reverse, CAAGCAGAAGACGGCATACGAGAT<Illumina index eight-nucleotide barcode>GTGACTGGAGTTCAGACGTGTGCTCTTCCGATCTCTACTATTCTTTCCCCTGCACTGTACC). The final PCR product was purified from a 2% agarose gel with the QIAquick Gel Extraction Kit. The concentration of different libraries was measured using the Qubit dsDNA HS (High Sensitivity) Assay Kit (Thermo) on a Qubit fluorometer (Thermo Fisher). The libraries were pooled at equal proportions and sequenced on an Illumina NextSeq 500 instrument for 76 single-read cycles at the Advanced Technology Genomics Core of the MDACC. As described previously^15,21,22^, MAGeCK (version 0.5.9.4)^52^ was used to calculate the read count of individual sgRNA species in different samples with the following parameters: ‘mageck count -l hct116.sgrna.library –control-sgrna hct116.sgrna.library.negctrl –norm-method control -n hct116.sgrna.count –sample-label D0,D21 –fastq files.fq’. DESeq2 (1.22.2)^53^ was used to calculate the statistical significance of differential expression for each sgRNA between day 0 and day 21. The read counts of individual sgRNA species were normalized to those of the mapped negative-control sgRNA species using ratio median normalization, and normalization factors were applied to all sgRNA species. Because the non-canonical ORFs are much shorter than the annotated ORFs, the sequence space for sgRNA design is more limited. Consequently, it is more difficult to design sgRNA species with good efficiency for the non-canonical ORFs than for the annotated ORFs. Therefore, instead of using the ORF- or gene-level summary statistics implemented in MAGeCK that require each ORF or gene to have a good number of effective sgRNA species for identifying candidate hits, we used a filter based on sgRNA-level results: the cryptic ORFs have at least two significantly depleted sgRNA species (sgRNA level, log_2_ (fold change) − log_2_ (1.5) and *P* < 0.05). We also required the lncRNA genes encoding these ORFs to be upregulated in COAD versus normal colon tissues (log_2_ (fold change) ≥ log_2_ (1.2), FDR < 0.01) for consideration as candidates of CRC dependency. To control for the potential sgRNA-mediated effect on the UTRs or CDS of the neighboring protein-coding genes of the cryptic ORFs, we considered a candidate cryptic ORF hit to be valid if there were at least two significantly depleted sgRNA species after removing (1) the sgRNA species that potentially affect the CDS of the annotated coding genes (regardless of whether the gene was essential or not) and (2) the sgRNA species that may affect the UTRs of the annotated coding genes that are essential genes in HCT-116 cells based on the Project Score database^24^. Given that the majority of the Cas9-mediated changes are <15 bp in size^23^, we considered an sgRNA to have a potential effect on the UTR or CDS of the neighboring coding gene if its putative cutting site was within 15 bp of the UTR or CDS of that gene. To remove redundancy in the ORFs that are encoded by different isoforms of the same gene, we selected the ORFs with the most significant Ribo-TISH-predicted *P* values for a given gene with ≤85% sequence identity.

### Real-time quantitative PCR with reverse transcription

RT–qPCR was performed similarly to what was described previously^15,21,22^. Briefly, total RNA was extracted using the RNeasy Mini kit (QIAGEN, 74104) according to the manufacturer’s instructions. cDNA synthesis was then performed with 1 µg of total RNA using the iScript cDNA Synthesis Kit (Bio-Rad, 1708890). RT–qPCR was performed using 2× Universal SYBR Green Fast qPCR Mix (ABclonal, RK21203) in the CFX96 Touch Real-Time PCR Detection System (Bio-Rad) according to the manufacturer’s instructions. All primers were synthesized by Sigma, and the sequences of the primers are listed in Supplementary Table 9. The gene encoding glyceraldehyde 3-phosphate dehydrogenase (GAPDH) was used as an internal control, and the fold change of gene expression was calculated using the 2^−ΔΔCt^ method.

### RNA sequencing

RNA-seq was performed as described previously^15,21,22^. Briefly, total RNA was isolated from cells using the RNeasy Mini kit (QIAGEN, 74104) and was treated with DNase I (QIAGEN, 79254). RNA-seq libraries were prepared from 2 µg of total RNA using the TruSeq Stranded mRNA Library Prep kit (Illumina, 20020594) according to the manufacturer’s instructions. The libraries were sequenced on an Illumina NextSeq 500 instrument (single end, 76 bp) at the Advanced Technology Genomics Core of the MDACC.

### ChIP sample preparation and ChIP–seq

As described in our previous study^15^, ChIP was performed following D. Odom’s group’s protocol with some adaptations^54^. In brief, at about 80–90% confluence, approximately 2 × 10^7^ cells were first cross-linked with 1% formaldehyde (methanol free, 16%, Thermo Scientific, 28908) at room temperature for 10 min and then quenched with 0.125 M glycine (final concentration) for 5 min. After washing with cold PBS three times, the cells were collected using a silicon scraper. Cell pellets were resuspended in 5 ml lysis buffer 1 (50 mM HEPES-KOH, pH 7.5, 140 mM NaCl, 1 mM EDTA, 10% glycerol, 0.5% NP-40, 0.25% Triton X-100) and rocked at 4 °C for 5 min, followed by centrifugation at 2,000*g* for 4 min at 4 °C. The cell pellets were then incubated with 5 ml LB2 buffer (10 mM Tris-HCl, pH 8.0, 200 mM NaCl, 1 mM EDTA, 0.5 mM EGTA) at 4 °C for 5 min with gentle rocking. Nuclei were pelleted by centrifuging the cells at 2,000*g* for 5 min and were resuspended in 1 ml LB3 buffer (10 mM Tris-HCl, pH 8.0, 100 mM NaCl, 1 mM EDTA, 0.5 mM EGTA, 0.1% sodium deoxycholate, 0.5% *N*-lauroylsarcosine). All lysis buffer contained protease inhibitors (Roche, 04693112001). Chromatin was sonicated to DNA fragments of around 200 bp using a Diagenode Bioruptor (three rounds of five cycles, 30 s on and 30 s off). Lysates were cleared by the addition of Triton X-100 to a final concentration of 1% and centrifugation at 2,000*g* for 10 min at 4 °C. A total of 50 µl of lysates was saved from each sample for input and stored at –80 °C until use. To prepare antibody-bound beads, 30 µl of magnetic beads (Invitrogen, Dynabeads) were washed three times with blocking buffer (1× PBS, 0.5% BSA) and incubated overnight with 5 µg of anti-SMC1A antibodies at 4 °C. For each ChIP, 900 µl of sonicated lysate from 2 × 10^7^ cells was incubated with antibody-bound beads overnight at 4 °C. Beads were washed six times with RIPA wash buffer (50 mM HEPES-KOH, pH 7.6, 500 mM LiCl, 1 mM EDTA, 1% NP-40, 0.7% sodium deoxycholate) and one time with TBS (20 mM Tris-HCl, pH 7.6, 150 mM NaCl) for 5 min each time at room temperature with gentle rocking. All washing buffers contain protease inhibitors. The beads were eluted twice with 50 µl elution buffer (50 mM Tris-HCl, pH 8, 10 mM EDTA, 1% SDS) for 10 min at 65 °C with rocking. Cross-linking was reversed by adding 6 µl of 5 M NaCl to the eluates, and samples were incubated at 65 °C overnight. RNA was degraded by incubation with 1 µl of 10 mg ml^−1^ RNase at 37 °C for 30 min, and proteins were digested by incubation with 2 µl of 20 mg ml^−1^ proteinase K (Thermo Fisher) at 56 °C for 2 h. DNA was then purified using the QIAquick PCR purification kit (QIAGEN, 28106). The samples were either analyzed by qPCR or processed for sequencing. ChIP–seq libraries were prepared from 10 ng of ChIP DNA using the TruSeq ChIP Library Preparation Kit according to the manufacturer’s instructions. The libraries were sequenced on an Illumina HiSeq 2500 system (single end, 50 bp) at the Avera Institute for Human Genetics.

### Immunoprecipitation and subcellular fractionation

IP, subcellular fractionation and western blotting were performed similarly as described previously^15,21^. For IP assays, cells were lysed in Pierce IP lysis buffer (Thermo Fisher, 87787) with protease inhibitor and 10 mM PMSF (Thermo Fisher, 36978). For IP of exogenous FLAG-tagged proteins, anti-FLAG M2 agarose beads (Sigma-Aldrich, A2220) were incubated with whole-cell lysates overnight with gentle rotation at 4 °C. For IP of endogenous proteins, the specific antibodies were first coupled to protein G magnetic beads (Invitrogen, 10004D) and then incubated with the cell lysates. After incubation, the beads were washed five times with washing buffer (10 mM Tris, pH 7.4, 1 mM EDTA, 1 mM EGTA, pH 8.0, 150 mM NaCl, 1% Triton X-100) and resuspended in SDS–PAGE sample buffer (Bio-Rad, 1610747). Eluted proteins and 5% of the whole-cell extracts were analyzed by immunoblot. IP from chromatin extracts was performed in the same way. To prepare chromatin extracts, cells were incubated with hypotonic lysis buffer for 15 min and centrifuged, and then the nuclear pellets were fixed with 1% paraformaldehyde for 10 min, followed by quenching with 0.125 M glycine for 5 min. The nuclear pellets were resuspended in 0.5% NP-40 for 15 min and sonicated for 21 cycles and centrifuged to collect the pellet (chromatin fraction).

To segregate and enrich nuclear and cytoplasmic proteins, subcellular protein fractionation kits for cultured cells (Thermo Scientific, 78840) and tissues (Thermo Scientific, 87790) were used for CRC cell lines and tumor tissues, respectively, according to the manufacturer’s instructions.

### Western blot

As described previously^15,21,22^, whole-cell lysates were generated using RIPA Lysis and Extraction Buffer (Thermo Fisher, 89900) supplemented with Protease Inhibitor Cocktail (Sigma, 11697498001) according to the manufacturer’s instructions. Protein concentration was measured by using the Bradford assay (Bio-Rad, 5000006). Proteins were separated with 4–15% or 4–20% Mini-PROTEAN TGX precast polyacrylamide gels (Bio-Rad) and then transferred to PVDF membranes (Millipore, GVWP04700) in transfer buffer (Invitrogen, LC3675) at 4 °C. Membranes were first blocked and incubated with specific antibodies overnight at 4 °C and then incubated with Immobilon Western Chemiluminescent HRP Substrate (Millipore, WBKLS0500) followed by analysis using the ChemiDoc Touch Imaging System (Bio-Rad).

### Immunofluorescence staining

Immunofluorescence staining was performed similarly as described previously^15^. HCT-116 and DLD-1 cells stably transduced to express FLAG-tagged SMIMP were seeded into four-well culture or chamber slides (Lab-Tek, 154917) with 30–50% confluency. Cells were washed with cold PBS and fixed using 4% paraformaldehyde for 15 min followed by permeabilization in 0.25% Triton X-100 solution for 10 min at room temperature.

The fixed cells were blocked with 10% normal goat serum (Life Technologies, PCN5000) in PBS for 30 min at room temperature and then incubated with anti-FLAG antibody (Sigma, F1804) at 1:500 in PBS overnight at 4 °C. After washing, the cells were incubated with fluorochrome-conjugated secondary antibody (Invitrogen, A32723) at 1:1,000 in PBS for 1 h at room temperature in the dark. The slips were mounted onto microscope slides with VECTASHIELD Mounting Medium containing DAPI (Vector Laboratories, H-1500). Images were captured by ZEISS LSM 880 confocal microscopy.

### LC–MS/MS analysis of exogenous or endogenous SMIMP

LC–MS/MS analysis for detecting microprotein-derived peptides was similarly performed as described previously^15^. Immunoprecipitated ectopically expressed FLAG-tagged or endogenously expressed SMIMP with an anti-FLAG (Sigma, F1804) or anti-SMIMP (ABclonal) antibody at a dilution of 1:100 were resolved on a NuPAGE 10% Bis-Tris Gel (Life Technologies), and the region corresponding to a molecular weight of 8–18 kDa was excised and processed for in-gel digestion using the trypsin enzyme. Tryptic peptides were analyzed on the nano-LC 1200 system (Thermo Fisher Scientific) coupled to an Orbitrap Fusion Lumos ETD (Thermo Fisher Scientific) mass spectrometer. Samples with or without 20 pg of spike-in heavy isotope-labeled arginine-LGSSLLSFTPR and NLHQPPLR peptide each were loaded on a two-column setup using a pre-column trap 2 cm × 100 µm in size (ReproSil-Pur Basic-C18, 1.9 µm, Dr. Maisch) and a 20-cm × 75-µm analytical column (ReproSil-Pur Basic-C18, 1.9 µm, Dr. Maisch) with a 110-min gradient of 6–30% acetonitrile and 0.1% formic acid at a flow rate of 200 nl min^−1^. The eluted peptides were directly electrosprayed into the mass spectrometer operated in data-dependent acquisition mode or PRM mode. For data-dependent acquisition mode, the full MS scan was acquired in the Orbitrap in the range of 300–1,400 *m*/*z* at a resolution of 120,000 followed by MS^2^ in the ion trap (HCD, 32% collision energy) with a dynamic exclusion time of 10 s. For PRM mode, the target precursor ions corresponding to the new ORF peptide sequences were isolated in quadrupole with an isolation width of 1.6 *m*/*z* for the whole duration. MS^2^ was carried out in the ion trap (rapid scan; scan range, 150–1,800 *m*/*z*; AGC, 2 × 10^4^; maximum injection time, 100 ms) using HCD fragmentation (HCD, 32% collision energy). The RAW file from MS was processed with Proteome Discoverer 1.4 (Thermo Scientific) using Mascot 2.4 (Matrix Science) with percolator against the new protein sequence and the human protein NCBI RefSeq database (updated 24 March 2020). The precursor ion tolerance and the product ion tolerance were set to 20 ppm and 0.5 Da, respectively. Dynamic modification of oxidation on methionine, protein N-terminal acetylation, deamidation on N or Q and carbamidomethyl on cysteine were allowed. The peptides identified from the Mascot result file were validated with an FDR of 5% and manually checked for correct assignment. The identification results and raw files were imported into Skyline software (version 21.2, MacCoss laboratory, University of Washington) for PRM analysis. MS^2^ chromatograms were evaluated by selecting PRM in the acquisition method and using the ion trap as a product mass analyzer with a resolution of 0.5 *m*/*z*.

### AP–MS-based mapping of protein–protein interactions

AP–MS was similarly performed as described previously^15,21,22^. HCT-116 cells stably expressing FLAG-tagged SMIMP, the ORF of AC012363.4 expressed with FLAG tag or FLAG-tagged GFP were lysed in Pierce IP lysis buffer (Thermo Fisher, 87787) with protease inhibitor and 10 mM PMSF (Thermo Fisher, 36978).

Whole-cell lysates were incubated with anti-FLAG M2 agarose beads (Sigma-Aldrich, A2220) overnight with gentle rotation at 4 °C. After incubation, the beads were washed five times with washing buffer (10 mM Tris, pH 7.4, 1 mM EDTA, 1 mM EGTA, pH 8.0, 150 mM NaCl, 1% Triton X-100) and resuspended in SDS–PAGE sample buffer (Bio-Rad, 1610747). The precipitated proteins on the beads were eluted by competition with 3× FLAG peptides (Sigma-Aldrich, F4799). The eluted proteins were resolved by SDS–PAGE and were sent to the Taplin MS Facility for LC–MS/MS analysis as described previously^55^. To identify the proteins that specifically interact with SMIMP, the following filters were applied: the number of identified unique peptides is ≥3 in the AP–MS of FLAG-tagged SMIMP and zero in that of FLAG-tagged GFP or the ORF of AC012363.4 expressed with FLAG tag. An additional filter of differential expression between COAD and normal colon tissue (log_2_ (fold change) ≥ 0.4, FDR < 0.01) was applied to identify candidate proteins (Supplementary Table 4) that may exert a tumor-promoting function in CRC.

### Cloning, expression and protein purification

The CDS of the C-terminal domain (1,033–1,233) of SMC1A that was fused with DNA for a StrepII tag at the C terminus was codon optimized and synthesized by Integrated DNA Technologies as a gene fragment. The synthesized gene fragment encoding SMC1A_1013–1233_–StrepII was then cloned into a derivative of the pET28b vector (Agilent Technologies) encoding a dual N-terminal His_6_–maltose-binding protein (MBP) tag with a tobacco etch virus (TEV) protease cleavage site by using the In-Fusion HD Cloning system (Takara Bio), thus producing a construct that can express His_6_–MBP–TEV_site_–SMC1A_1013–1233_–StrepII under the control of the T7 *lac* promoter. The MBP tag was introduced to increase both yield and solubility during expression. The construct that can express wild-type or mutant His_6_–SMIMP–GST under the control of the T7 *lac* promoter was similarly produced. The integrity of the resulting plasmids was confirmed by DNA sequencing as well as by restriction enzyme digestion.

The *E. coli* strain Rosetta 2 (DE3) (Agilent Technologies) was transformed with the pET28b His_6_–MBP–TEV_site_–SMC1A_1013–1233_–StrepII or pET28b wild-type or mutant His_6_–SMIMP–GST expression plasmid and grown overnight in terrific broth medium containing chloramphenicol (34 µg ml^−1^) and kanamycin (50 µg ml^−1^) at 37 °C. The overnight culture was used to inoculate terrific broth containing kanamycin (50 µg ml^−1^), and the culture was incubated with shaking at 37 °C until the optical density at 610 nm reached 0.8. After the culture was chilled to 4 °C, 0.5 mM isopropyl 1-thio-β-d-galactopyranoside was added, and the culture was shaken for 24 h at 20 °C. Cells were collected by centrifugation at 5,000*g* for 15 min.

To purify recombinant His_6_–MBP–TEV_site_–SMC1A_1013–1233_–StrepII, cells were resuspended in 50 mM Tris-HCl (pH 8.0) buffer containing 500 mM NaCl, 20 mM imidazole and 10% glycerol. Cells expressing His_6_–SMIMP–GST were resuspended in 50 mM Tris-HCl (pH 8.0) buffer containing 500 mM NaCl, 5 mM β-mercaptoethanol, 20 mM imidazole and 10% glycerol. The homogeneous suspension was lysed with two passes through an M-110P Microfluidizer (Microfluidics) at 20,000 psi and then centrifuged for 25 min at 100,000*g* and 4 °C. The supernatant containing recombinant His_6_–MBP–TEV_site_–SMC1A_1013–1233_–StrepII or His_6_–SMIMP–GST was then applied to a HisTrap HP 5-ml column (GE Healthcare) for nickel-affinity chromatography. For His_6_–MBP–TEV_site_–SMC1A_1013–1233_–StrepII, a thorough wash of the HisTrap column with PBS buffer containing 30 mM imidazole was followed by elution with PBS buffer containing 500 mM imidazole. For His_6_–SMIMP–GST, a thorough wash of the HisTrap column with PBS buffer containing 30 mM imidazole and 5 mM β-mercaptoethanol was followed by elution with PBS buffer containing 5 mM β-mercaptoethanol and 500 mM imidazole. The eluted sample containing His_6_–MBP–TEV_site_–SMC1A_1013–1233_–StrepII or His_6_–SMIMP–GST was buffer exchanged with PBS or PBS buffer containing 1 mM dithiothreitol, respectively, to remove imidazole or β-mercaptoethanol by using a HiPrep 26/10 desalting column (GE Healthcare). The buffer-exchanged sample containing His_6_–MBP–TEV_site_–SMC1A_1013–1233_–StrepII was loaded onto a StrepTrap HP 5-ml column (GE Healthcare) for Strep-Tactin affinity chromatography, followed by a thorough wash with PBS buffer, and then eluted with PBS buffer containing 2.5 mM desthiobiotin. The buffer-exchanged sample containing His_6_–SMIMP–GST was loaded onto a GSTrap HP 5-ml column (GE Healthcare) for GST-affinity chromatography, followed by a thorough wash with PBS buffer containing 1 mM dithiothreitol and then eluted with PBS buffer containing 15 mM reduced glutathione.

Finally, the purified proteins or microproteins were polished with size exclusion chromatography using a 120-ml HiLoad 16/600 Superdex 200 column (GE Healthcare), with PBS as the elution buffer. Fractions containing purified His_6_–MBP–TEV_site_–SMC1A_1013–1233_–StrepII or wild-type or mutant His_6_–SMIMP–GST were pooled and concentrated by using Amicon Ultra-15 Centrifugal Filters with an NMWL of 10 kDa (Merck Millipore). The whole process was conducted on the ÄKTA Pure System (GE Healthcare). To remove the His_6_–MBP–TEV_site_ tag from purified His_6_–MBP–TEV_site_–SMC1A_1013–1233_–StrepII, His_6_-tagged TEV protease was added with a mass ratio of 1:100 and incubated overnight at 4 °C. The reaction mixture was then applied to a HisTrap HP 5-ml column, and the flow-through fraction (that is, SMC1A_1013–1233_–StrepII) was collected and concentrated.

### GST pulldown and in vitro co-immunoprecipitation

GST pulldown was performed with the MagneGST Pull-Down System (Promega, V8870) according to the manufacturer’s instructions. The MagneGST Pull-Down System provides glutathione (GSH)-linked magnetic particles that allow simple immobilization of GST-fusion bait proteins and can be easily captured by the magnet. In brief, purified recombinant C-terminus GST-fused wild-type SMIMP or the M4 deletion mutant SMIMP (M) tagged with 6× His at the N terminus (His_6_–SMIMP or SMIMP (M)–GST) or GST tag was immobilized onto MagneGST particles, and the purified GST tag was used as a negative control. After being washed with PBS three times, the MagneGST particles carrying GST or His_6_–SMIMP or SMIMP (M)–GST proteins were resuspended in MagneGST Binding/Wash Buffer containing 1% BSA. The purified recombinant SMC1A C-terminal domain (1,033–1,233) (SMC1A_1013–1233_–StrepII) was suspended in the same MagneGST Binding/Wash Buffer and was incubated with MagneGST particles carrying GST or His_6_–SMIMP or SMIMP (M)–GST proteins at room temperature for 30 min. After incubation, the MagneGST particles were washed six times with PBS containing 0.02% Triton X-100. After the washing step, the proteins bound to the MagneGST particles were eluted with SDS loading buffer for western blot analysis. For in vitro co-IP, 4 µg of anti-MBP antibody (Proteintech, 66003-1-Ig) was first coupled to 50 µl of protein G magnetic beads (Invitrogen, 10004D) and then incubated with the purified MBP tag or His_6_–MBP–TEV_site_–SMC1A_1013–1233_–StrepII protein for 30 min at room temperature, and MBP served as a negative control. After incubation, the beads were washed three times with PBS containing proteinase inhibitors and then incubated with the purified GST and His_6_–SMIMP or SMIMP (M)–GST protein for 30 min at room temperature. The beads were washed five times with washing buffer (10 mM Tris, pH 7.4, 1 mM EDTA, 1 mM EGTA, pH 8.0, 150 mM NaCl, 1% Triton X-100) and were then eluted with SDS–PAGE sample buffer (Bio-Rad, 1610747). The eluted proteins were analyzed by western blot.

### RNAi, CRISPR–Cas9 knockout and ORF overexpression

RNAi-mediated knockdown, CRISPR–Cas9-mediated knockout and ORF overexpression for individual genes or ORFs were similarly performed as described previously^15,22^. For loss-of-function experiments using CRISPR–Cas9-mediated gene knockout in pooled cell populations, the negative-control sgRNA or gene-specific sgRNA was cloned into the lentiCRISPR v2 (Addgene, 52961) vector. To produce lentiviruses, HEK293T cells were cotransfected with pCMV-VSV-G, psPAX2 and the sgRNA-expressing lentiCRISPR v2 plasmid using jetPRIME (Polyplus-transfection, 114-15). Lentiviruses were collected 48 h after transfection and were then used to infect cell lines in the presence of polybrene (Sigma, TR-1003) before puromycin selection for 4 d. The knockout efficiency of individual sgRNA species was determined by western blot after 10 d of puromycin selection, and then the cells were collected for functional assays. For siRNA-mediated knockdown experiments, one negative-control siRNA and two pre-designed on-target siRNA species (Sigma-Aldrich) were used. A total of 1 × 10^5^ cells were plated in each well of 12-well plates. In each well, 40 pmol siRNA species were transfected into cells using Lipofectamine RNAiMAX Transfection Reagent (Thermo Fisher, 13778150), and total RNA was extracted 48 h after transfection for RT–qPCR analysis of knockdown efficiency. For shRNA-mediated knockdown, the shRNA sequences were cloned into the PLKO.1 TRC vector. To produce lentiviruses, HEK293T cells were cotransfected with pCMV-VSV-G, psPAX2 and the shRNA-expressing PLKO.1 TRC plasmid using jetPRIME. Lentiviruses were collected 48 h after transfection and then were used for infecting HCT-116, DLD-1 or HT-29 cell lines in the presence of polybrene before puromycin selection for 2 d. Total RNA and protein were collected 4 d after puromycin selection. RT–qPCR and western blot were used to determine shRNA-mediated knockdown efficiency at RNA and protein levels, respectively. The 5′ UTR of SMIMP (DNA), SMIMP (DNA), the 5′ UTR of the ORF of AC012363.4 and the ORF of AC012363.4 were amplified from cDNA extracted from HCT-116 cells and cloned into the pLenti-CMV-Blast DEST vector. DNA encoding the wild-type and mutant SMIMP was synthesized (Twist Bioscience) and cloned into the pLVX-puro or pLenti-CMV-Blast DEST vector with sequence for FLAG tag. DNA encoding wild-type and mutant SMC1A was cloned into pcDNA3.1 with sequence for HA tag or the pLenti-CMV-Blast DEST vector. For ORF overexpression in 293FT cells, the plasmids were transfected with jetPRIME transfection regents. For ORF overexpression in HCT-116, DLD-1 and HT-29 cells, lentivirus particles were produced in 293FT cells and then collected for transducing the cell lines. Expression was determined by western blot assays and then the related cells were collected for functional assays. All sgRNA, siRNA and shRNA sequences are listed in Supplementary Table 9.

### Cell growth and clonogenic assays

Cell growth and clonogenic assays were performed as described previously^15,21,22^. Cell growth was assessed using the CCK-8 (Dojindo Molecular Technologies, CK04-13) as described previously^21^ and by the manufacturer. Briefly, cells were trypsinized, resuspended and seeded at 1,000 cells per well in 96-well plates, and each treatment condition and time point was in triplicate. The cells were then incubated with 10 µl CCK-8 solution for 2 h at 37 °C with 5% CO_2_. Absorbance was measured at 450 nm using a microplate reader (BioTek Synergy H1). In siRNA-mediated gene-silencing experiments, cells were seeded 48 h after siRNA transfection. For stable knockdown or knockout experiments based on shRNA or sgRNA, shRNA- or sgRNA-transduced cells were seeded 4 d (shRNA) or 10 d (sgRNA) after puromycin selection, respectively. For the colony-formation assay, shRNA- or sgRNA-transduced cells were seeded at 1,000 cells per well in six-well plates or 400 cells per well in 12-well plates with each treatment condition in triplicate. The medium was changed every day. After 2 weeks, cells were fixed with 100% methanol for 30 min and stained with 0.5% crystal violet in PBS for 2 h. Plates were then washed with distilled water and photographed with the ChemiDoc Touch Imaging System (Bio-Rad). The ColonyArea ImageJ (version 1.53) plugin was used to calculate colony area percentages.

### Xenograft experiments

For CRISPR–Cas9-based loss-of-function experiments, a total of 2 × 10^6^ HCT-116 or DLD-1 cells stably transduced with the lentiCRISPR v2 vector (Addgene, 52961) containing a non-targeting sgRNA (negative control) or individual sgRNA species targeting SMIMP along with Cas9 were injected subcutaneously into the left flank region of *Foxn1*^nu/nu^ athymic nude mice (5 weeks old, female) that were purchased from the MDACC ERO Breeding Core to establish CRC xenograft tumors (*n* = 7 for each group).

Mice were housed under pathogen-free conditions with a 12-h dark–light cycle at 25 °C (ambient temperature) with 48–60% humidity. Tumor volume was measured every 3 d for 30 d using the formula (tumor volume = (*L* × *W*^2^) ÷ 2), where *L* represents the largest tumor diameter and *W* represents the perpendicular tumor diameter. The tumors were removed on day 30 for subsequent analysis. For the rescue experiments, a total of 2 × 10^6^ HCT-116 cells stably expressing SMIMP with a wild-type (ATG) or mutant (AGG) start codon or the empty vector control (EV), were stably transduced with a negative-control shRNA (shNC) or individual ELFN1-AS1-targeting shRNA species and were injected subcutaneously into the left flank region of *Foxn1*^nu/nu^ athymic nude mice (5 weeks old, female), to establish CRC xenograft tumors (*n* = 8 for each group). Tumor volume was measured every 4 d for 31 d. The tumors were removed on day 31 for subsequent analysis. All mouse experiments were performed according to the NIH Guide for the Care and Use of Laboratory Animals (National Academies, 2011) and were approved by the Institutional Animal Care and Use Committee of the UT MDACC (IACUC study 00001077-RN02).

### Human tissue sample analysis

All freshly frozen tissue samples (Supplementary Table 3) used for western blotting were purchased from US Biolab. The human paraffin-embedded tissue array (US Biomax, CO1506) was used for RNAscope in situ hybridization assays. Briefly, the TMA slides were hydrated with deionized water, followed by antigen-retrieval treatment at 95 °C, hybridized with RNAscope Probe—Hs-ELFN1-AS1-C1 (*Homo sapiens* ELFN1 antisense RNA 1 (ELFN1-AS1) transcript variant 3 lncRNA, ACD, 1082631-C1), counterstained with hematoxylin and coverslipped. All staining was performed with the Leica BOND RX automated stainer. The stained slides were scanned, and signal copy numbers were analyzed using Halo version 3.3.2541.345 (Algorithm Indica Labs-ISH version 4.1.3) with a minimum of 300 cells in each core. RNA expression was quantified using the *H*-score, which was calculated based on RNA expression categorized into five grades: 0, copy number ≤1 per cell; 1+, copy number 2–10 per cell; 2+, copy number 11–20 per cell; 3+, copy number 21–30 per cell; 4+, copy number copy ≥31 per cell or with clustered signals.

### RNA-seq and ChIP–seq data analysis

RNA-seq and ChIP–seq data analysis and integrative analyses of TCGA and GTEx data were similarly performed as described previously^15,22^. RNA-seq reads were first trimmed to remove adaptor sequences and masked for low-complexity and low-quality sequences and were then mapped to the human genome (GRCh38) and the GENCODE version 22 transcriptome, using STAR version 2.6.1b (ref. ^51^) with the parameters: ‘–outSAMunmapped Within –outFilterType BySJout –twopassMode Basic –outSAMtype BAM SortedByCoordinate’. Gene-level raw read counts were calculated using the htseq-count function of HTSeq (0.11.0)^56^ based on the aligned and sorted BAM files. Normalization of read counts and differential gene expression analysis were performed using DESeq2 (1.22.2)^53^. The filters of basemean ≥ 1, |log_2_ (fold change)| ≥ log_2_ (1.5) and FDR ≤ 0.05 were used to define differentially expressed genes for downstream analysis. SMC1A ChIP–seq reads were first trimmed using Trim Galore (version 0.6.5) (https://www.bioinformatics.babraham.ac.uk/projects/trim_galore/), a wrapper around two tools: cutadapt version 2.8 (https://github.com/marcelm/cutadapt/) and FastQC version 0.11.5 (https://github.com/chgibb/FastQC0.11.5/, https://www.bioinformatics.babraham.ac.uk/projects/fastqc/) and were then mapped to the human genome (GRCh38) using Bowtie 2 (version 2.4.1)^57^. The resulting sorted BAM files were converted into BedGraph and bigWig formats using BEDTools (version 2.24.0)^58^ and UCSC bedGraphToBigWig (version 4)^59^. ChIP–seq peaks were identified using MACS2 (version 2.1.2)^60^ with the parameters ‘macs2 callpeak -t ChIP.bam -c INPUT.bam -g hs –outdir output -n NAME 2 > NAME.callpeak.log’. BETA (version 1.0.7)^61^ was used to annotate peaks that were associated with genes of interest (FDR ≤ 0.05). Gene ontology enrichment analysis was performed with DAVID^62^. TCGA and GTEx RNA-seq data joint analysis was performed based on the combined cohort TCGA TARGET GTEx from UCSC Toil RNA-seq Recompute^63^. Normalized gene expression in TPM and clinical information were extracted with a customized script for differential gene expression analysis between tumor and normal tissues and among different cancer types. The Wilcoxon rank-sum test was used to identify genes with deregulated expression between tumors and the corresponding normal tissues. The consensus molecular subtypes of CRC were defined and assigned to individual TCGA CRC tumors as described previously^25^. Differentially expressed genes between individual molecular subtypes and the rest of the tumors were identified using the Wilcoxon rank-sum test based on normalized gene expression data. Multiple-testing corrections were performed using the Benjamini–Hochberg procedure^64^. Kaplan–Meier survival curves were used to show survival distributions, and the log-rank test was used to assess the corresponding statistical significance. Survival analysis was performed using the ‘survival’ and ‘survminer’ package in R (version 4.2.1).

### Statistical analysis

When applicable, experimental data are presented as mean ± s.d. Unless stated otherwise, *P* values were calculated using two-tailed Student’s *t*-test in GraphPad Prism 9.0 or Excel. Statistical tests used in different experiments are indicated in the figure legends.

### Reporting summary

Further information on research design is available in the Nature Portfolio Reporting Summary linked to this article.

## Online content

Any methods, additional references, Nature Portfolio reporting summaries, source data, extended data, supplementary information, acknowledgements, peer review information; details of author contributions and competing interests; and statements of data and code availability are available at 10.1038/s41594-023-01117-1.

### Supplementary information

Supplementary Information Supplementary Figs. 1 and 2 and the supporting data for these figures.

Reporting Summary

Supplementary Tables 1–9 Supplementary Table 1. Information about each sgRNA in the library, cryptic non-canonical ORFs in HCT-116 cells, mapping between ORFs and their corresponding sgRNA species and the final list of ORFs identified as CRC dependent. *P* values of non-canonical ORFs predicted from ribo-seq data were determined by an unpaired Wilcoxon test implemented in Ribo-TISH. *P* values from the CRISPR screen for sgRNA-level differential analyses were determined by the Wald test implemented in DESeq2. *P* values from the differential gene expression analysis related to TCGA data were determined by an unpaired Wilcoxon test. Supplementary Table 2. Constituent peptides of SMIMP identified by non-targeted or targeted LC–MS/MS analysis. Supplementary Table 3. Information about frozen CRC tumors and matched normal colon tissues. Supplementary Table 4. List of proteins identified by AP–MS. *P* values from the differential gene expression analysis with TCGA data were determined by an unpaired Wilcoxon test. Supplementary Table 5. List of annotated differentially expressed protein-coding genes identified by RNA-seq in CRC cells upon sgRNA-mediated knockout of SMIMP. There were three replicates for each condition. *P* values were determined by the Wald test implemented in DESeq2. Supplementary Table 6. List of annotated differentially expressed protein-coding genes identified by RNA-seq in CRC cells upon shRNA-mediated knockdown of SMC1A. There were three replicates for each condition. *P* values were determined by the Wald test implemented in DESeq2. Supplementary Table 7. List of SMC1A ChIP–seq peaks in CRC cells. There were two replicates for ChIP–seq experiments. *P* values were determined based on a null model of Poisson distribution with MACS2. Supplementary Table 8. List of annotated protein-coding genes that are co-repressed by SMIMP and SMC1A, have at least one SMC1A-binding site within ±10 kb of their TSSs and are significantly downregulated in COAD compared with normal colon tissues. Supplementary Table 9. Sequences of sgRNA species, siRNA species, shRNA species, sgRNA library linker sequences and primers.

### Source data

Source Data Fig. 2 Statistical source data.

Source Data Fig. 2 Unprocessed western blots and/or gels.

Source Data Fig. 3 Unprocessed western blots and/or gels.

Source Data Fig. 4 Statistical source data.

Source Data Fig. 4 Unprocessed western blots and/or gels.

Source Data Fig. 5 Unprocessed western blots and/or gels.

Source Data Fig. 6 Statistical source data.

Source Data Fig. 6 Unprocessed western blots and/or gels.

Source Data Fig. 7 Statistical source data.

Source Data Fig. 8 Statistical source data.

Source Data Fig. 8 Unprocessed western blots and/or gels.

Source Data Extended Data Fig. 2 Statistical source data.

Source Data Extended Data Fig. 2 Unprocessed western blots and/or gels.

Source Data Extended Data Fig. 3 Unprocessed western blots and/or gels.

Source Data Extended Data Fig. 4 Statistical source data.

Source Data Extended Data Fig. 4 Unprocessed western blots and/or gels.

Source Data Extended Data Fig. 5 Statistical source data.

Source Data Extended Data Fig. 6 Statistical source data.

Source Data Extended Data Fig. 6 Unprocessed western blots and/or gels.

Source Data Extended Data Fig. 7 Statistical source data.

Source Data Extended Data Fig. 8 Statistical source data.

Source Data Extended Data Fig. 9 Statistical source data.

Source Data Extended Data Fig. 10 Statistical source data.

Source Data Extended Data Fig. 10 Unprocessed western blots and/or gels.

## Data Availability

The raw sequencing data generated and/or analyzed during the current study were deposited at GEO (GSE184322). The human genome (GRCh38) was used to map the raw sequencing reads. All data that support the findings of this study are available in the paper, in the Supplementary Information and/or at GEO. Source data are provided with this paper.

## Code availability

The codes and scripts used in this study are available at both GitHub (https://github.com/Proteome-EPI-Genome/CRC.ORF.project) and Zenodo (10.5281/zenodo.8219149).
