# Supplementary material for: CRISPR–Cas9-based functional interrogation of unconventional translatome reveals human cancer dependency on cryptic non-canonical open reading frames
Source: Nat Struct Mol Biol. 2023 Nov 6;30(12):1878–92. doi: 10.1038/s41594-023-01117-1 (PMC10716047; doi:10.1038/s41594-023-01117-1)
Supplement: Supplementary file 1 — Supplementary Figs. 1 and 2 and the supporting data for these figures. [file 41594_2023_1117_MOESM1_ESM.pdf]

nature structural & molecular biology

Article https://doi.org/10.1038/s41594-023-01117-1

CRISPR–Cas9-based functional interrogation of unconventional translatome reveals human cancer dependency on cryptic non-canonical open reading frames

In the format provided by the authors and unedited

Figure S1

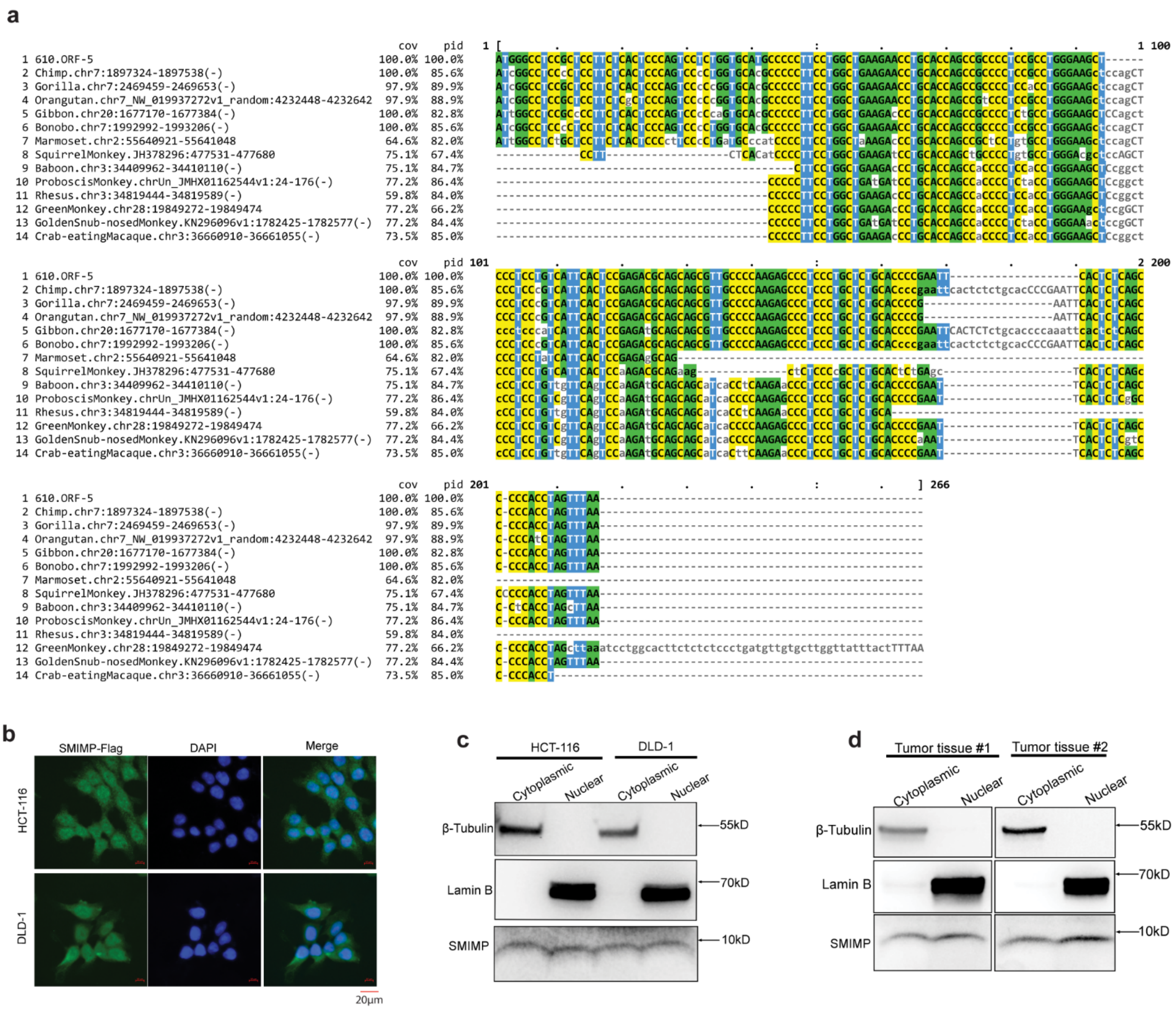
a
cov pid
1 610.ORF-5 100.0% 100.0%
2 Chimp.chr7:1897324-1897538(-) 100.0% 85.6%
3 Gorilla.chr7:2469459-2469653(-) 97.9% 89.9%
4 Orangutan.chr7_NW_019937272v1_random:4232448-4232642 97.9% 88.9%
5 Gibbon.chr20:1677170-1677384(-) 100.0% 82.8%
6 Bonobo.chr7:1992992-1993206(-) 100.0% 85.6%
7 Marmoset.chr2:55640921-55641048 64.6% 82.0%
8 SquirrelMonkey.JH378296:477531-477680 75.1% 67.4%
9 Baboon.chr3:34409962-34410110(-) 75.1% 84.7%
10 ProboscisMonkey.chrUn_JMHX01162544v1:24-176(-) 77.2% 86.4%
11 Rhesus.chr3:34819444-34819589(-) 59.8% 84.0%
12 GreenMonkey.chr28:19849272-19849474 77.2% 66.2%
13 GoldenSnub-nosedMonkey.KN296096v1:1782425-1782577(-) 77.2% 84.4%
14 Crab-eatingMacaque.chr3:36660910-36661055(-) 73.5% 85.0%
b
SMIMP-Flag
DAPI
Merge
HCT-116
DLD-1
20µm
c
HCT-116
DLD-1
Cytoplasmic
Nuclear
β-Tubulin
Lamin B
SMIMP
55kD
70kD
10kD
d
Tumor tissue #1
Tumor tissue #2
Cytoplasmic
Nuclear
β-Tubulin
Lamin B
SMIMP
55kD
70kD
10kD

Supplementary Figure 1. a, Multiple sequence alignment of the SMIMP (610-ORF-5) DNA sequence and its homologous sequences from the non-human primate species. The homologous sequences in non-human species were identified by BLAST. The multiple sequence alignment was performed by Clustal Omega (https://www.ebi.ac.uk/Tools/msa/clustalo/) and Mview (https://www.ebi.ac.uk/Tools/msa/mview/) was employed for visualization. **b**, The sub-cellular localization of the FLAG-tagged SMIMP was determined by the immunofluorescence staining with an anti-FLAG antibody in the HCT-116/DLD-1 cells stably expressing FLAG-tagged SMIMP, where cell nuclei were stained with DAPI. **c**, **d**, The endogenous expression of SMIMP in cytoplasmic and nuclear fraction of (**c**) HCT-116/DLD-1 cells or (**d**) CRC tumor tissues (Table S3) was detected by western blot, where β-tubulin and lamin B served as cytoplasmic and nuclear marker, respectively. Western blot data are representative of at least three independent experiments.

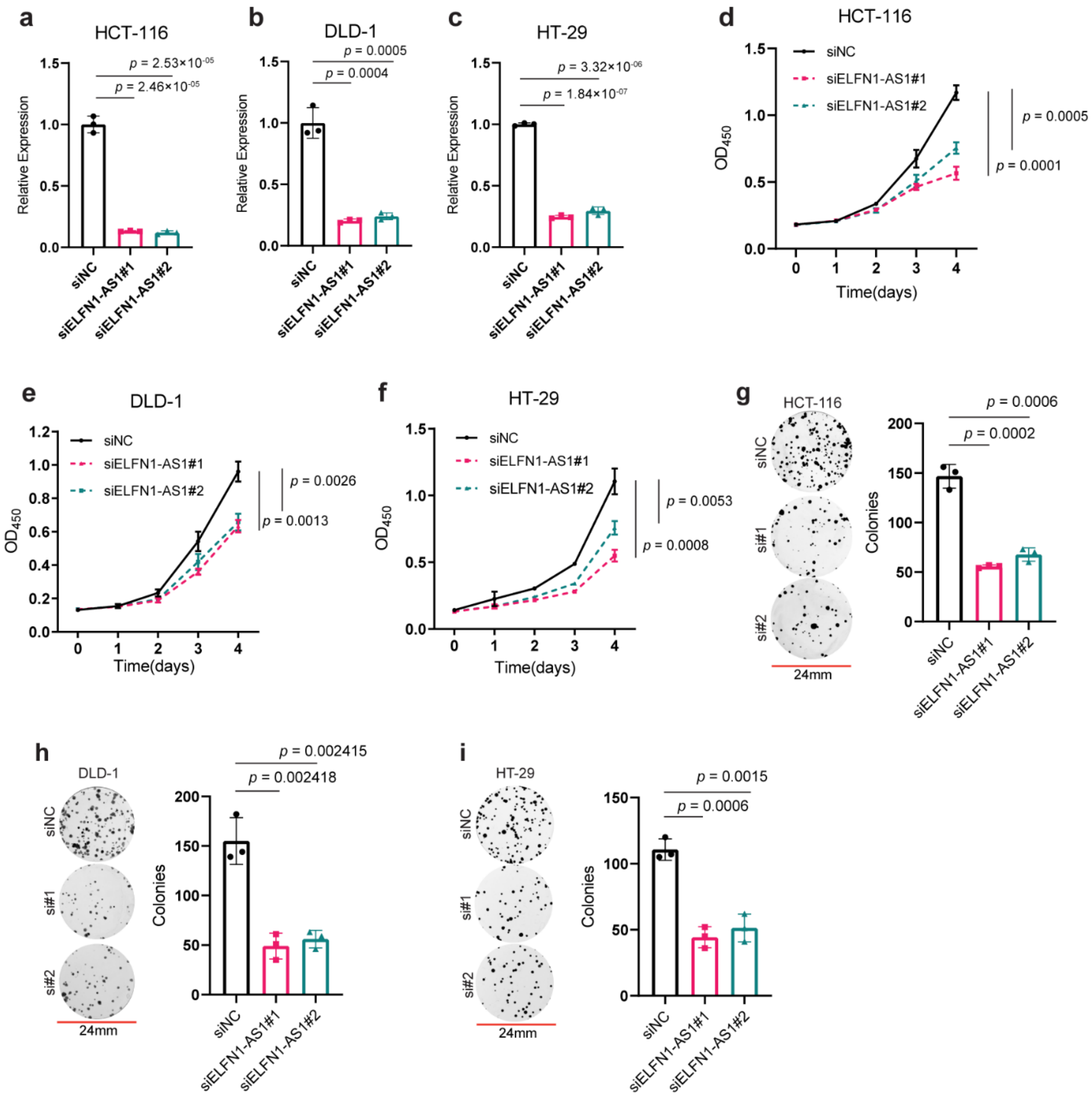
a
HCT-116
p = 2.53×10-05
p = 2.46×10-05
Relative Expression
siNC
siELFN1-AS1#1
siELFN1-AS1#2
b
DLD-1
p = 0.0005
p = 0.0004
c
HT-29
p = 3.32×10-06
p = 1.84×10-07
d
HCT-116
siNC
siELFN1-AS1#1
siELFN1-AS1#2
p = 0.0005
p = 0.0001
OD450
Time(days)
e
DLD-1
p = 0.0026
p = 0.0013
f
HT-29
p = 0.0053
p = 0.0008
g
HCT-116
p = 0.0006
p = 0.0002
Colonies
siNC
si#1
si#2
24mm
h
DLD-1
p = 0.002415
p = 0.002418
i
HT-29
p = 0.0015
p = 0.0006

Supplementary Figure 2. a-c, QRT-PCR was performed to determine the siRNA-mediated knockdown efficiency for ELFN1-AS1 in (**a**) HCT-116, (**b**) DLD-1, and (**c**) HT-29 cells. **d-f**, The growth of the (**d**) HCT-116, (**e**) DLD-1, and (**f**) HT-29 cells transfected with the negative control siRNA (siNC) or individual siRNAs targeting ELFN1-AS1 was monitored with CCK-8 assay each day for 4 days. **g-i**, The representative pictures of clonogenic growth and the bar graph quantifying the colonies formed by (**g**) HCT-116, (**h**) DLD-1 and (**i**) HT-29 cells that were transfected with the siNC or siRNAs targeting ELFN1-AS1. Pictures of clonogenic growth are representative of at least three independent experiments. When applicable, data are shown as mean+/-standard deviation (SD), n=3. *P*-values were determined by an unpaired two-tailed Student's *t*-test.

Source data Supplementary Figure 1

Full unedited gel for Supplementary Figure 1c and 1d

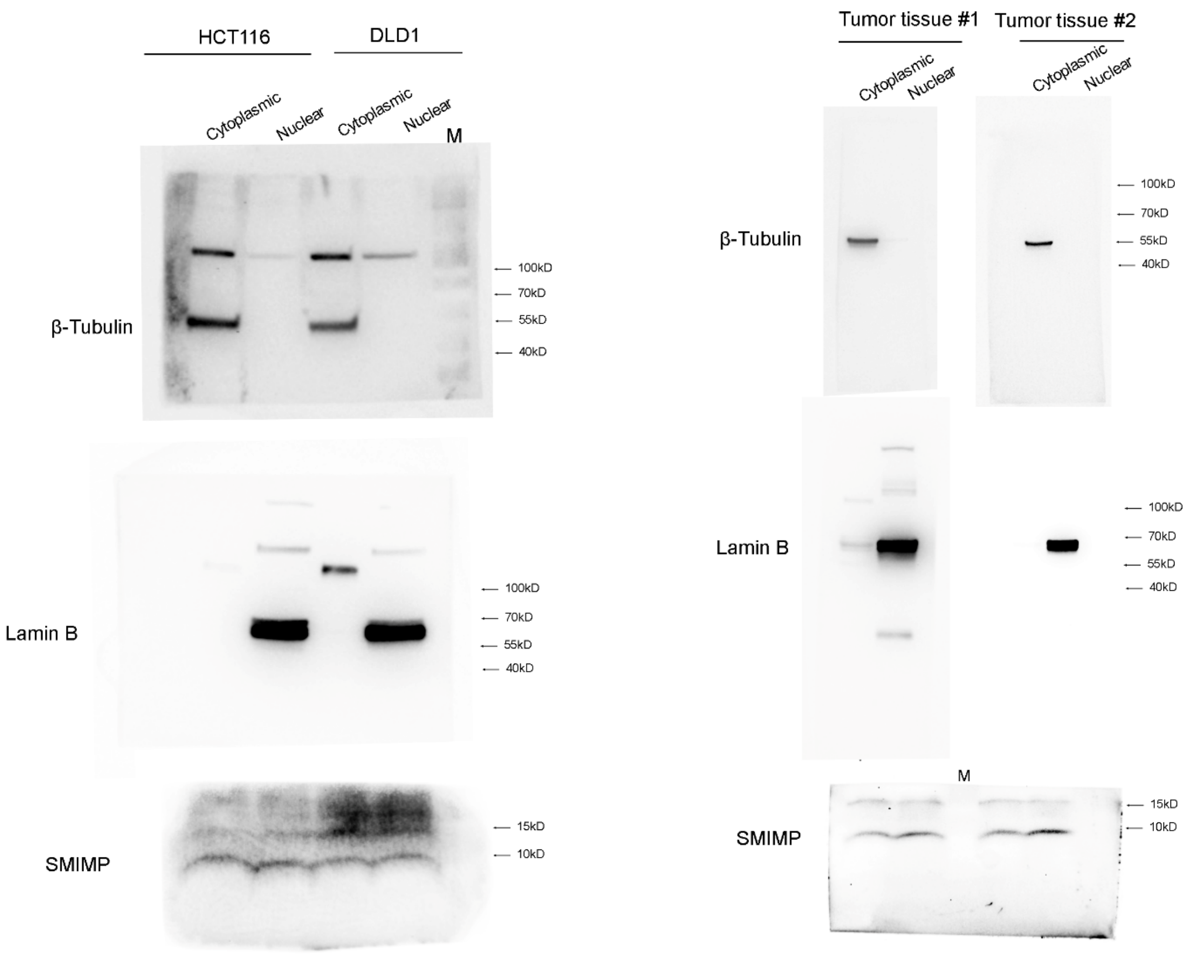

Source data Supplementary Figure 2

Supplementary Figure 2a

siNC	1.07	0.93	1.00
siELFN1-AS1#1	0.13	0.13	0.14
siELFN1-AS1#2	0.11	0.12	0.13

Supplementary Figure 2b

siNC	1.14	0.94	0.92
siELFN1-AS1#1	0.21	0.21	0.19
siELFN1-AS1#2	0.27	0.24	0.21

Supplementary Figure 2c

siNC	0.99	1.00	1.01
siELFN1-AS1#1	0.24	0.26	0.24
siELFN1-AS1#2	0.32	0.31	0.26

Supplementary Figure 2d

days	siNC			siELFN1-AS1#1			siELFN1-AS1#2		
0	0.181	0.181	0.183	0.18	0.177	0.183	0.178	0.178	0.179
1	0.208	0.208	0.208	0.21	0.213	0.207	0.204	0.211	0.207
2	0.323	0.347	0.339	0.285	0.297	0.294	0.272	0.305	0.283
3	0.65	0.623	0.749	0.441	0.463	0.485	0.452	0.537	0.531
4	1.105	1.197	1.205	0.518	0.562	0.616	0.776	0.705	0.781

Supplementary Figure 2e

days	siNC			siELFN1-AS1#2			siELFN1-AS1#1		
0	0.14	0.13	0.125	0.142	0.13	0.13	0.14	0.139	0.128
1	0.149	0.169	0.146	0.16	0.15	0.15	0.157	0.155	0.139
2	0.241	0.25	0.21	0.199	0.192	0.196	0.178	0.202	0.185
3	0.607	0.526	0.494	0.46	0.432	0.365	0.381	0.352	0.348
4	1.026	0.947	0.908	0.619	0.714	0.64	0.674	0.601	0.626

Supplementary Figure 2f

days	siNC			siELFN1-AS1#1			siELFN1-AS1#2		
0	0.143	0.14	0.141	0.131	0.131	0.13	0.134	0.136	0.134
1	0.287	0.203	0.187	0.187	0.161	0.16	0.185	0.158	0.164

2	0.297	0.306	0.309	0.219	0.212	0.22	0.233	0.25	0.237
3	0.501	0.476	0.488	0.282	0.283	0.277	0.344	0.341	0.335
4	0.994	1.172	1.15	0.501	0.586	0.563	0.795	0.779	0.701

Supplementary Figure 2g

siNC			siELFN1-AS1#1			siELFN1-AS1#2		
156	151	133	54	57	56	60	70	73

Supplementary Figure 2h

siNC			siELFN1-AS1#1			siELFN1-AS1#2		
144	139	182	61	35	51	59	63	46

Supplementary Figure 2i

siNC			siELFN1-AS1#1			siELFN1-AS1#2		
120	105	107	45	52	36	51	41	62
