# Supplementary material for: CRISPR–Cas9-based functional interrogation of unconventional translatome reveals human cancer dependency on cryptic non-canonical open reading frames
Source: Nat Struct Mol Biol. 2023 Nov 6;30(12):1878–92. doi: 10.1038/s41594-023-01117-1 (PMC10716047; doi:10.1038/s41594-023-01117-1)
Supplement: Supplementary file 2 — Reporting Summary [file 41594_2023_1117_MOESM2_ESM.pdf]

nature portfolio

Corresponding author(s): Yiwen Chen

Last updated by author(s): 08/11/2023

Reporting Summary

Nature Portfolio wishes to improve the reproducibility of the work that we publish. This form provides structure for consistency and transparency in reporting. For further information on Nature Portfolio policies, see our Editorial Policies and the Editorial Policy Checklist.

Statistics

For all statistical analyses, confirm that the following items are present in the figure legend, table legend, main text, or Methods section.

n/a	Confirmed	
☐	☒	The exact sample size (*n*) for each experimental group/condition, given as a discrete number and unit of measurement
☐	☒	A statement on whether measurements were taken from distinct samples or whether the same sample was measured repeatedly
☐	☒	The statistical test(s) used AND whether they are one- or two-sided<br>*Only common tests should be described solely by name; describe more complex techniques in the Methods section.*
☒	☐	A description of all covariates tested
☐	☒	A description of any assumptions or corrections, such as tests of normality and adjustment for multiple comparisons
☐	☒	A full description of the statistical parameters including central tendency (e.g. means) or other basic estimates (e.g. regression coefficient) AND variation (e.g. standard deviation) or associated estimates of uncertainty (e.g. confidence intervals)
☐	☒	For null hypothesis testing, the test statistic (e.g. *F*, *t*, *r*) with confidence intervals, effect sizes, degrees of freedom and *P* value noted<br>*Give P values as exact values whenever suitable.*
☒	☐	For Bayesian analysis, information on the choice of priors and Markov chain Monte Carlo settings
☒	☐	For hierarchical and complex designs, identification of the appropriate level for tests and full reporting of outcomes
☐	☒	Estimates of effect sizes (e.g. Cohen's *d*, Pearson's *r*), indicating how they were calculated

Our web collection on statistics for biologists contains articles on many of the points above.

Software and code

Policy information about availability of computer code

Data collection	RNA-seq, Ribo-seq, sgRNA-seq and ChIP-seq raw data were collected from illumina NextSeq 500 and illumina HiSeq2500 sequining platform. RNA-seq and sgRNA-seq libraries were sequenced on an Illumina NextSeq 500 (single end 76 bp), at the Advanced Technology Genomics Core of MDACC. ChIP-seq and Ribo-seq libraries were sequenced on an Illumina HiSeq2500 (single end 50 bp), at the Avera Institute for Human Genetics. TCGA and GTEx RNA-seq data joint analysis was performed based on the combined cohort: TCGA TARGET GTEx, from UCSC Toil RNA-seq Recompute Normalized gene expression in TPM and clinical information were extracted with R software(version 4.2.1). Mass spectrometry raw data were collected from a nano-LC 1200 system (Thermo Fisher Scientific, San Jose, CA) coupled to an Orbitrap Fusion™ Lumos ETD (Thermo Fisher Scientific, San Jose, CA) mass spectrometry.
Data analysis	A description of the computational data analysis and code availability has been included in the manuscript. The computational tools that were used include Trim Galore (v0.6.5), cutadapt v2.8, FastQC v0.11.5, Bowtie2 (v2.4.1), BEDTools (v2.24.0), UCSC bedGraphToBigWig (v4), MACS2 (v2.1.2), BETA (v1.0.7) , STAR(v.2.6.1b), MAGeCK (v0.5.9.4), HTSeq (v0.11.0), DESeq2(1.22.2), FastQC(v0.11.5), R software(v4.2.1), Skyline(v21.2), GraphPad Prism(9.0), and ImageJ(v1.53).

For manuscripts utilizing custom algorithms or software that are central to the research but not yet described in published literature, software must be made available to editors and reviewers. We strongly encourage code deposition in a community repository (e.g. GitHub). See the Nature Portfolio guidelines for submitting code & software for further information.

Data

Policy information about availability of data

All manuscripts must include a data availability statement. This statement should provide the following information, where applicable:

- Accession codes, unique identifiers, or web links for publicly available datasets
- A description of any restrictions on data availability
- For clinical datasets or third party data, please ensure that the statement adheres to our policy

The raw sequencing data generated and/or analyzed during the current study were deposited to GEO (GSE184322).
https://www.ncbi.nlm.nih.gov/geo/query/acc.cgi?acc=GSE184322

human genome (GRCh38) used to map the raw sequencing reads.
https://genome.ucsc.edu/cgi-bin/hgTracks?db=hg38&lastVirtModeType=default&lastVirtModeExtraState=&virtModeType=default&virtMode=0&nonVirtPosition=&position=chr6%3A36675800%2D36687825&hgsid=1242821679_0Ym9BfFKiBB9SocwbugDHfV1ZtnG

Field-specific reporting

Please select the one below that is the best fit for your research. If you are not sure, read the appropriate sections before making your selection.

☒ Life sciences ☐ Behavioural & social sciences ☐ Ecological, evolutionary & environmental sciences

For a reference copy of the document with all sections, see nature.com/documents/nr-reporting-summary-flat.pdf

Life sciences study design

All studies must disclose on these points even when the disclosure is negative.

Sample size	The sample sizes are stated in figure legends. No statistical methods were used to predetermine the sample size. For ChIP-seq, two biological replicates were used. For RNA-seq, Ribo-seq and CRISPRI/Cas9 screen, at least three biological replicates were used. For cell growth analysis(CCK8), clonogenic assay, RT-qPCR and ChIP-qPCR, at least three biological replicates were used. For western blotting and image-based analysis, representative data from at least three independent experiments has been shown.
Data exclusions	No data were excluded from analysis.
Replication	Reported results were consistently replicated across multiple experiments with all replicates generating similar results. The numbers of replicates are presented in the figure legends. ChIP-seq expriments were replicated 2 times. RNA-seq and CRIPSR/Cas9 screen were replicated at least 3 times. Cell growth analysis, clonogenic asssy, image-based analysis, western blotting, RT-qPCR/ChIP-qPCR were examined in at least three independent experiments.
Randomization	For xenograft experiments, mice were randomized into three groups (with 7/8 for each group). For the other experiments, there was no randomization as these experiments were performed in cell lines and are not affected by the order of the recording for the results data and cell lines used for both control and treatments are cultured under same condition.
Blinding	Data collection and analysis was not blinded to the operator, because conditions were well controlled and the results are quantitative and did not require subjective judgment or interpretation.

Reporting for specific materials, systems and methods

We require information from authors about some types of materials, experimental systems and methods used in many studies. Here, indicate whether each material, system or method listed is relevant to your study. If you are not sure if a list item applies to your research, read the appropriate section before selecting a response.

Materials & experimental systems

n/a	Involved in the study	
☐	☒	Antibodies
☐	☒	Eukaryotic cell lines
☒	☐	Palaeontology and archaeology
☐	☒	Animals and other organisms
☐	☒	Human research participants
☒	☐	Clinical data
☒	☐	Dual use research of concern

Methods

n/a	Involved in the study	
☐	☒	ChIP-seq
☒	☐	Flow cytometry
☒	☐	MRI-based neuroimaging

Antibodies

Antibodies used

Primary antibodies:
mouse monoclonal anti-Flag M2 antibody (Sigma-Aldrich #F1804, M2, monoclonal), WB:1:5000, IP:5ug IF: 1:500, ChIP:3ug
rabbit polyclonal anti-SMC1 antibody (Betthyl # A300-055A), WB:1;2000, IP:2-5ug, ChIP: 5ug
rabbit monoclonal anti-β-Tubulin antibody (9F3) (CST #2128), WB: 1:1000, IHC: 1:50, IF:1:50
rabbit monoclonal anti-HA-Tag antibody (C29F4) (CST #3724), WB:1:1000, IP:1:50,IHC:1:800, IF:1:800, ChIP: 1:50
rabbit polyclonal anti-EPB41L5 antibody (Invitrogen #PA5-58008), WB:0.04-0.4ug/mL, IHC:1:200, IF:0.25-2ug/mL
rabbit polyclonal anti-ELFN1 antibody (US Biological #035032), WB:1:1000
rabbit polyclonal anti-beta-tubulin antibody (Proteintech #10068-1-AP), WB:1:2000, IP: 4ug, IHC: 1:200, IF: 1:800
mouse monoclonal MBP tag antibody (Proteintech #66003-1-Ig), WB: 1:1000-1:8000, IP: 0.5-4.0ug, IF: 1:500-1:2000
rabbit polyclonal anti-GST tag antibody (Proteintech #10000-0-AP), WB:1:1000-1:4000
mouse monoclonal anti-His tag antibody (Proteintech #66005-1-Ig), WB:1:5000, IP: 0.5-4.0 ug, IF:1:200-1:800
rabbit polyclonal anti-SMIMP antibody (ABclonal), WB: 1:500

Secondary antibodies:
goat Anti-rabbit IgG, HRP-linked Antibody(CST #7074), WB:1:3000
horse Anti-mouse IgG, HRP-linked Antibody(CST #7076), WB: 1:3000
Goat anti-Mouse IgG (H+L) Highly Cross-Adsorbed Secondary Antibody, Alexa Fluor™ Plus 488 (Invitrogen #A32723) at 1:1000

Validation

mouse monoclonal anti-Flag M2 antibody (Sigma-Aldrich #F1804, M2, monoclonal)
supplier website: mouse mAb recognizes the FLAG peptide sequence at the N-terminus, Met-N-terminus,C-terminus, and internal sites of the fusion protein. Validated for WB, IP, IF, ChIP. (Nature communications, 10(1), 3684-3684 (2019-08-17);Molecular biology of the cell, 24(11), 1619-1637 (2013-04-12);Nature communications, 10(1), 3733-3733 (2019-08-21);PLoS genetics, 15(7), e1008240-e1008240 (2019-08-01))

rabbit polyclonal anti-SMC1 antibody (Betthyl # A300-055A)
supplier website: The epitope recognized by A300-055A maps to a region between residue 1175 and the C-terminus of human Structural Maintenance of Chromosomes 1 using the numbering given in entry NP_006297. 2 (GeneID 8243). Validated for IHC, IP, WB (Nature (2022) 606 (7912), 188-196 DOI: 10.1038/s41586-022-04727-9; Nature (2021) 601 (7892), 268-273 DOI: 10.1038/s41586-021-04261-0; Nature (2020) 578 (7795), 472-476 DOI: 10.1038/s41586-019-1910-z); Nature (2020) 584 (7819), 142-147 DOI: 10.1038/s41586-020-2454-y).Tested for ChIP in several publications (Nucleic Acids Res 2022 Jan 11;50(1):207-226; Nat Genet 2022 Mar;54(3):283-294.).

rabbit monoclonal anti-β-Tubulin antibody(9F3) (CST #2128)
supplier website: β-Tubulin (9F3) Rabbit mAb detects endogenous levels of total β-tubulin protein, and does not cross-react with recombinant α-tubulin. Validated for WB, IHC and IFC (Nature 2023 Jul;619(7971):819-827; EMBO Rep, 2023 Jul 5;24(7):e56458; JCI Insight, 2023 Jul 11;e165568.).

rabbit monoclonal anti-HA-Tag antibody (C29F4) (CST #3724)
supplier website:HA-Tag (C29F4) Rabbit mAb detects exogenously expressed proteins containing the HA epitope tag. The antibody may cross-react with a protein of unknown origin ~100kDa. Validated for WB, IHC, IF and ChIP (Science, 2023 Jun 7;26(7):107059; Cell Death Differ, 2023 Aug 30(8):1973-1987; Nucleic Acids Res, 2011 Mar;39(5):1811-22).

rabbit polyclonal anti-EPB41L5 antibody (Invitrogen #PA5-58008)
supplier website:Immunogen sequence: LLASLTENLI DHTVAPQVSS TSMITPRWIV PQSGAMSNGL AGCEMLLTGK EGHGNKDGIS LISPPAPFLVDAVTS. Validated for WB, IHC and IF.

rabbit polyclonal anti-ELFN1 antibody (US Biological #035032)
supplier website: Recognizes human ELFN1. Suitable for use in Western Blot and ELISA. Other applications not tested. Validated for WB.

rabbit polyclonal anti-beta-tubulin antibody (Proteintech #10068-1-AP)
supplier website:10068-1-AP targets Beta Tubulin in WB, IP, IHC, IF, FC, ELISA applications and shows reactivity with human, mouse, rat samples. Validated for WB, IHC, IP and IF.(Cell, 2020 Mar 19;180(6):1081-1097.e24; Acta Pharm Sin B, 2021,Nov;11(11):3553-3566; Nat Commun, 2019 Oct 11;10(1):4664).

mouse monoclonal MBP tag antibody(Proteintech #66003-1-Ig)
supplier website: 66003-1-Ig targets MBP tag in WB, IP, IHC, IF, CoIP, ELISA applications and shows reactivity with recombinant protein samples. Validated for WB, IHC, IP and IF.(Nat Commun, 2023 Feb 11;14(1):767; Mol Cell, 2022 Apr 21;82(8):1528-1542.e10; Nat Commun, 2021 Aug 13;12(1):4926).

rabbit polyclonal anti-GST tag antibody(Proteintech #10000-0-AP)
supplier website:10000-0-AP targets GST Tag in WB, IP, IF, FC, CoIP, ChIP, ELISA applications and shows reactivity with recombinant protein samples. Validated for WB.(Signal Transduct Target Ther, 2022 Dec 27;7(1):400; Gastroenterology, 2023 Mar;164(3):424-438.).

mouse monoclonal anti-His tag antibody (Proteintech #66005-1-Ig)
supplier website: 66005-1-Ig targets 6*His, His-Tag in WB, IP, IHC, IF, FC, CoIP, ChIP, Cell treatment, ELISA applications and shows reactivity with recombinant protein samples. Validated for WB, IF and IP.(Nature, 2019 Jul;571(7763):127-131; Nature, 2021 Dec;600(7888):308-313.)

rabbit polyclonal anti-SMIMP antibody (ABclonal)
Rabbit anti-SMIMP (ABclonal), This antibody was produced and verified by ABclonal company and also been validated for WB by our

group(a validation description has been included in the results).

goat Anti-rabbit IgG, HRP-linked Antibody(CST #7074)
supplier website:Designed for use with rabbit polyclonal and monoclonal antibodies, this affinity purified goat anti-rabbit IgG (heavy and light chain) antibody is conjugated to horseradish peroxidase(HRP) for chemiluminescent detection. This product is thoroughly validated with CST primary antibodies and will work optimally with the CST western immunoblotting protocol, ensuring accurate and reproducible results.(Nucleus, 2023 Dec;14(1):2165602; J Exp Med, 2023 Sep 4;220(9):e20221751; Mol Med Rep, 2023 Sep;28(3):166.)

horse Anti-mouse IgG, HRP-linked Antibody(CST #7076)
supplier website:Affinity purified horse anti-mouse IgG (heavy and light chain) antibody is conjugated to horseradish peroxidase(HRP) for chemiluminescent detection. This product is thoroughly validated with CST primary antibodies and will work optimally with the CST western immunoblotting protocol, ensuring accurate and reproducible results.(Nucleus, 2023 Dec;14(1):2165602;Bioact Mater, 2023 Apr 19;27:337-347;Mol Med Rep, 2023 Sep;28(3):161.).

Goat anti-Mouse IgG (H+L) Highly Cross-Adsorbed Secondary Antibody
supplier website:Anti-Mouse secondary antibodies are affinity-purified antibodies with well-characterized specificity for mouse immunoglobulins and are useful in the detection, sorting or purification of its specified target. Secondary antibodies offer increased versatility enabling users to use many detection systems (e.g. HRP, AP, fluorescence). (Cell Death Dis ,2023 Jul 17;14(7):444; EMBO J, 2023 Jul 17;42(14):e112614; Front Endocrinol (Lausanne), 2023 Jun 23;14:1155639.)

Eukaryotic cell lines

Policy information about cell lines

Cell line source(s)	Human colorectal cancer cell lines HCT-116, DLD-1, HT-29, SW480, RKO, LoVo and CaO2, and immortalized colon epithelial cell line CRL-1831 were obtained from American Type Culture Collection (ATCC) and cultured according to the instructions described from ATCC. Human embryonic kidney cell line HEK293T and HEK293FT cells were from Characterized Cell Line Core Facility at MD Anderson Cancer Center and were cultured in Dulbecco's modified Eagle's medium (DMEM, Hyclone #SH30022.01). HCT-116 were cultured in McCoy's 5a medium (Coring #10-050-CV). DLD-1 and HT-29 cells were cultured in RPMI- 1640 (Hyclone #SH30027.1).
Authentication	No further authentication was performed for cell lines.
Mycoplasma contamination	All cell lines were tested negative for Mycoplasma contamination.
Commonly misidentified lines (See ICLAC register)	No commonly misidentified cell lines were used.

Animals and other organisms

Policy information about studies involving animals; ARRIVE guidelines recommended for reporting animal research

Laboratory animals	The Foxn1 nu/nu athymic nude mice (5-week-old female) were used for xenograft experiments. Mice were housed under pathogen-free conditions with a 12-h dark–light cycle at 25°C ambient temperature and 48-60% humidity.
Wild animals	No wild animals were used in this study.
Field-collected samples	No field-collected samples were used in this study.
Ethics oversight	All mouse experiments were approved by MD Anderson's Institutional Animal Care and Use Committee (IACUC) under the protocol 00001077-RN02.

Note that full information on the approval of the study protocol must also be provided in the manuscript.

Human research participants

Policy information about studies involving human research participants

Population characteristics	No human research participants were involved in the current study. All the fresh frozen tissue samples used for western blotting were purchased as de-identified tissues from US Biolab Corporation Inc. The human paraffin embedded tissue array used for RNAscope in situ hybridization (ISH) assays were purchased as de-ientified tissue array from US Biomax, Inc.
Recruitment	*Describe how participants were recruited. Outline any potential self-selection bias or other biases that may be present and how these are likely to impact results.*
Ethics oversight	*Identify the organization(s) that approved the study protocol.*

Note that full information on the approval of the study protocol must also be provided in the manuscript.

ChIP-seq

Data deposition

☒ Confirm that both raw and final processed data have been deposited in a public database such as GEO.

☒ Confirm that you have deposited or provided access to graph files (e.g. BED files) for the called peaks.

Data access links<br>*May remain private before publication.*	https://www.ncbi.nlm.nih.gov/geo/query/acc.cgi?acc=GSE184322
Files in database submission	SMC1A1 ChIP seq: input and IP bigwig files, narrowPeak files
Genome browser session<br>(e.g. UCSC)	https://genome.ucsc.edu/cgi-bin/hgTracks?db=hg38&lastVirtModeType=default&lastVirtModeExtraState=&virtModeType=default&virtMode=0&nonVirtPosition=&position=chr6%3A36675800%2D36687825&hgsid=1242821679_0Ym9BfFKiBB9SocwbugDHfV1ZtnG

Methodology

Replicates	2 biological replicates
Sequencing depth	Samples TotalReads UniqueMappedReads ReadLength library<br>Input#1 33263287 24468571 50 single-end<br>Input#2 32094399 23822533 50 single-end<br>SMC1A ChIP#1 37026129 27964072 50 single-end<br>SMC1A ChIP#2 32211443 24344344 50 single-end
Antibodies	Bethyl Laboratories (A300-055A)
Peak calling parameters	macs2 callpeak -t ChIP.bam -c INPUT.bam -g hs --outdir output -n NAME 2> NAME.callpeak.log
Data quality	Totally output 81072 peaks, and 62231 peaks pass the filter: FDR < 0.05 & fold enrichment> 5.
Software	The ChIP-seq reads were first trimmed by Trim Galore (v0.6.5), a wrapper around two tools: cutadapt v2.8 and FastQC v0.11.5, and were then mapped to the human genome (GRCh38), using Bowtie2 (v2.4.1). The resulting sorted BAM files were converted into bedGraph and bigWig formats using BEDTools (v2.24.0) and UCSC bedGraphToBigWig (v4). The ChIP-seq peaks were identified by MACS2 (v2.1.2) with the parameters "macs2 callpeak -t ChIP.bam -c INPUT.bam -g hs --outdir output -n NAME 2> NAME.callpeak.log". BETA (v1.0.7) was used to annotate the peaks that are associated with genes of interest.
