# Supplementary figures and images for: CRISPR–Cas9-based functional interrogation of unconventional translatome reveals human cancer dependency on cryptic non-canonical open reading frames
Source: Nat Struct Mol Biol. 2023 Nov 6;30(12):1878–92. doi: 10.1038/s41594-023-01117-1 (PMC10716047; doi:10.1038/s41594-023-01117-1)

Full unedited gel for Figure 2b

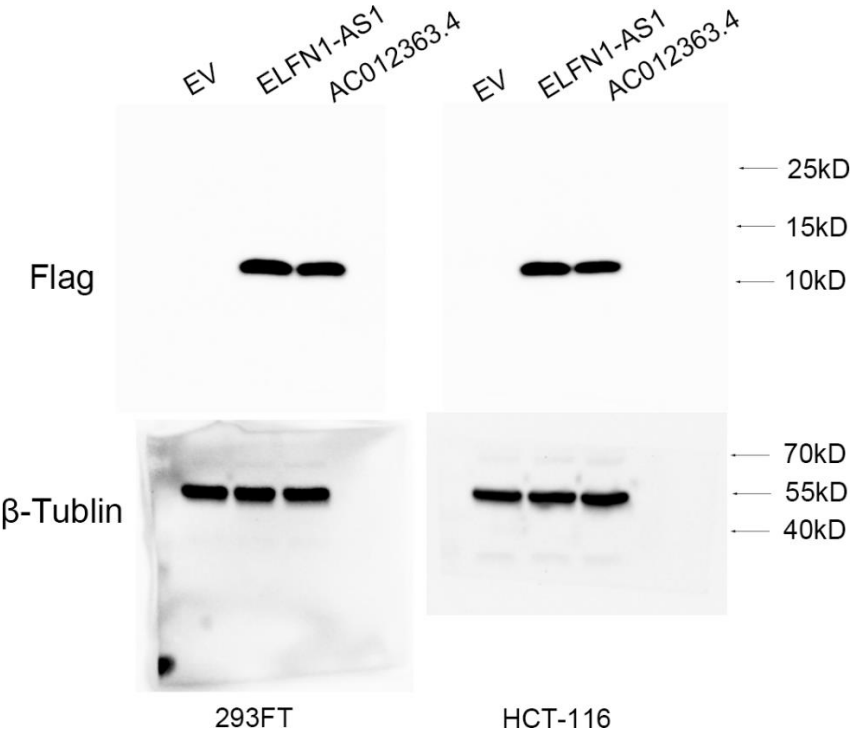

Full unedited gel for Figure 2c

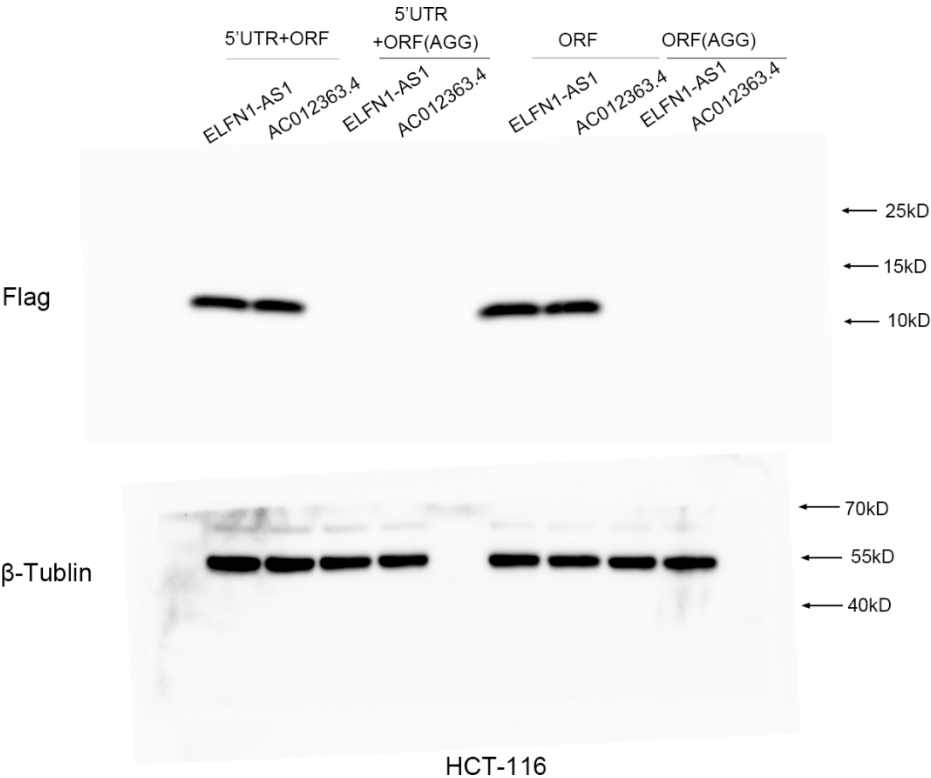

Supplement: Supplementary file 5 — Unprocessed western blots and/or gels. [file 41594_2023_1117_MOESM5_ESM.pdf]

Full unedited gel for Figure 4e

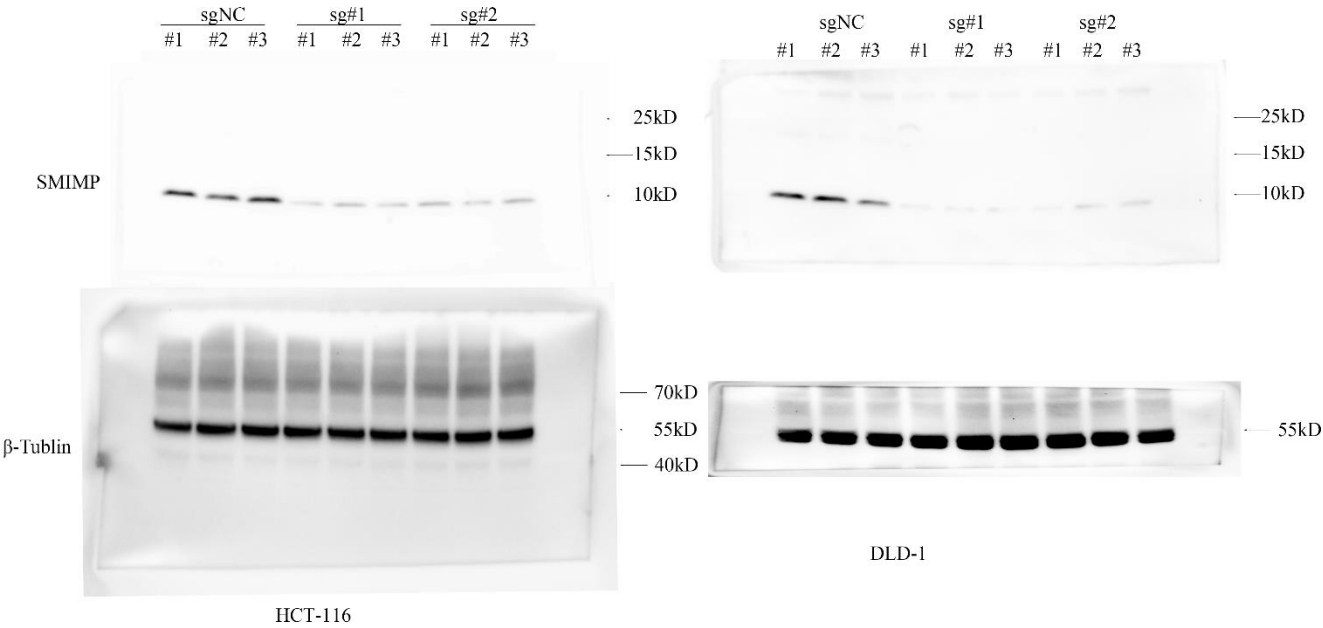

Supplement: Supplementary file 8 — Unprocessed western blots and/or gels. [file 41594_2023_1117_MOESM8_ESM.pdf]

Full unedited gel for Figure 6b

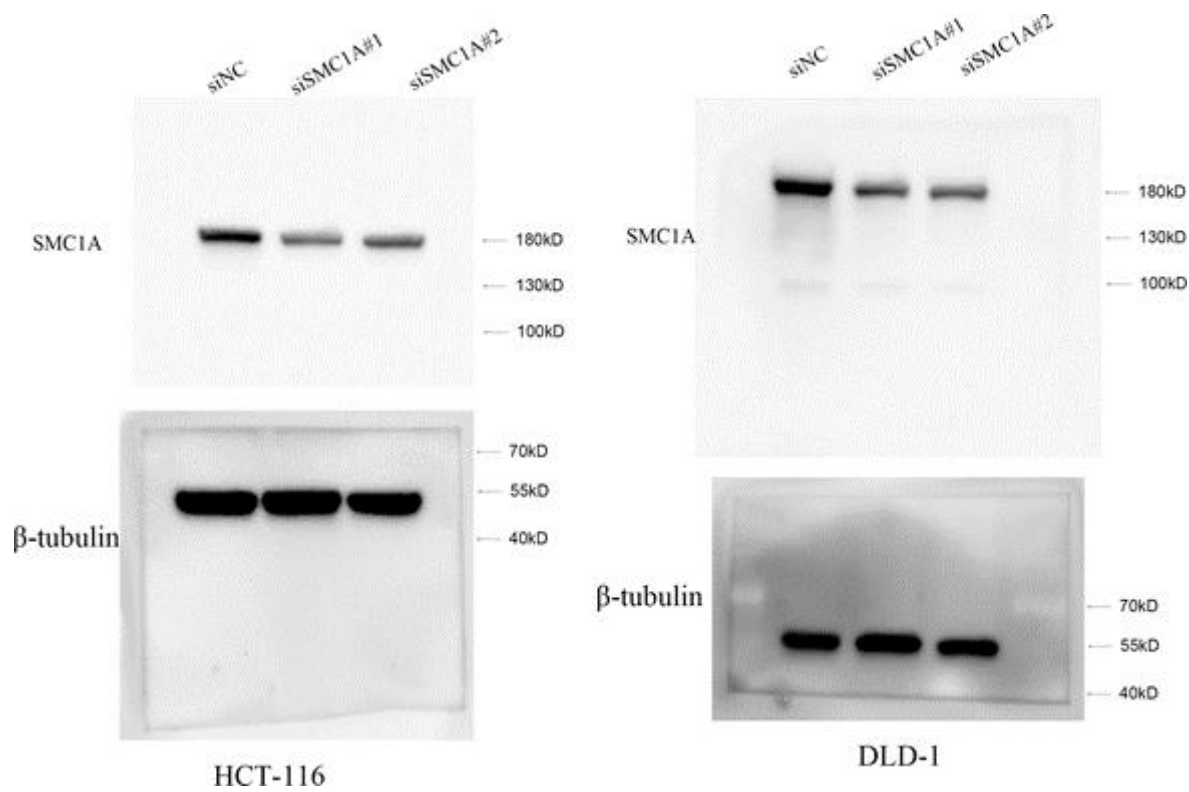

Supplement: Supplementary file 11 — Unprocessed western blots and/or gels. [file 41594_2023_1117_MOESM11_ESM.pdf]

Full unedited gel for Figure 8 e, h, k

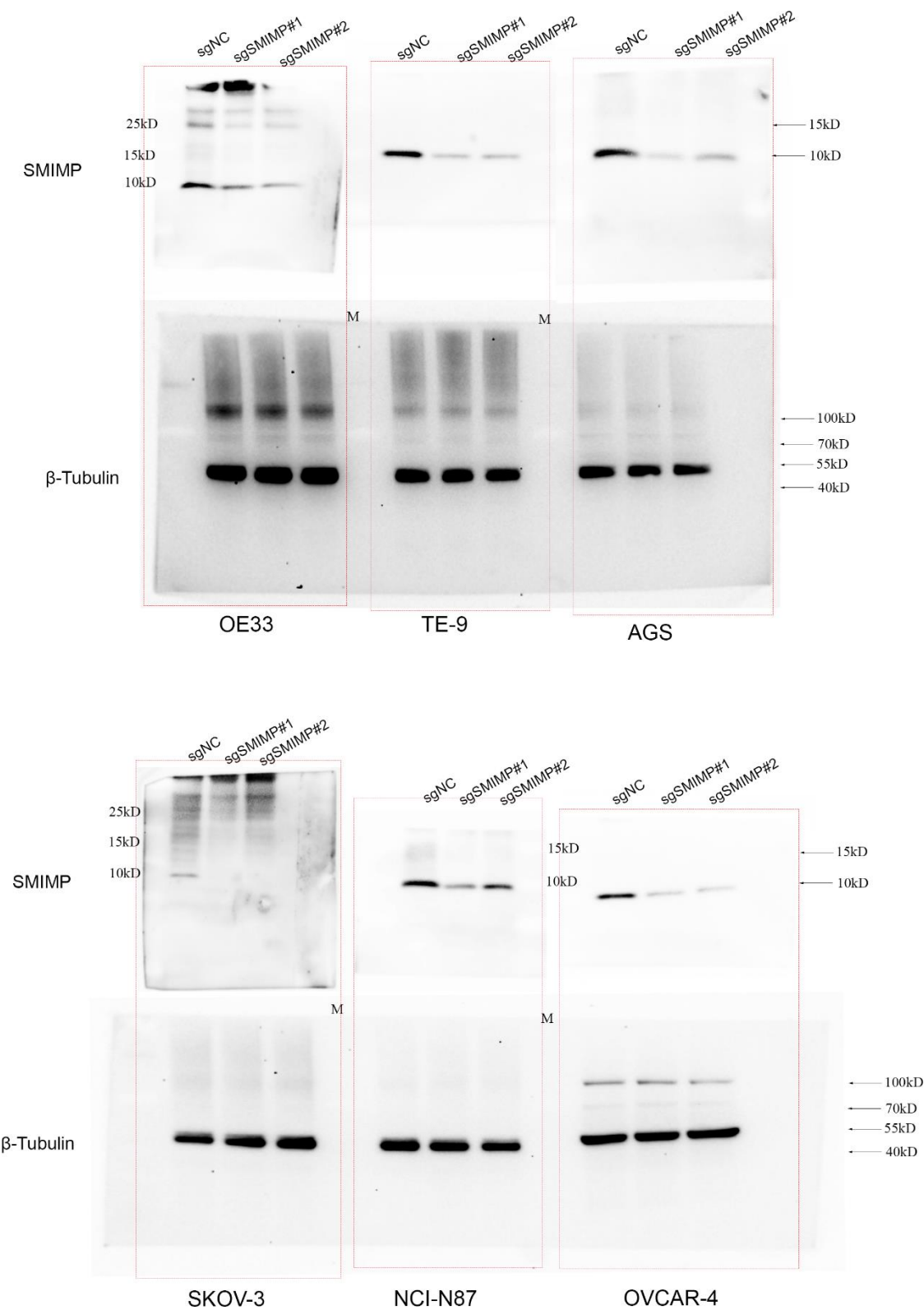

Supplement: Supplementary file 14 — Unprocessed western blots and/or gels. [file 41594_2023_1117_MOESM14_ESM.pdf]

Full unedited gel for extended data figure 3c

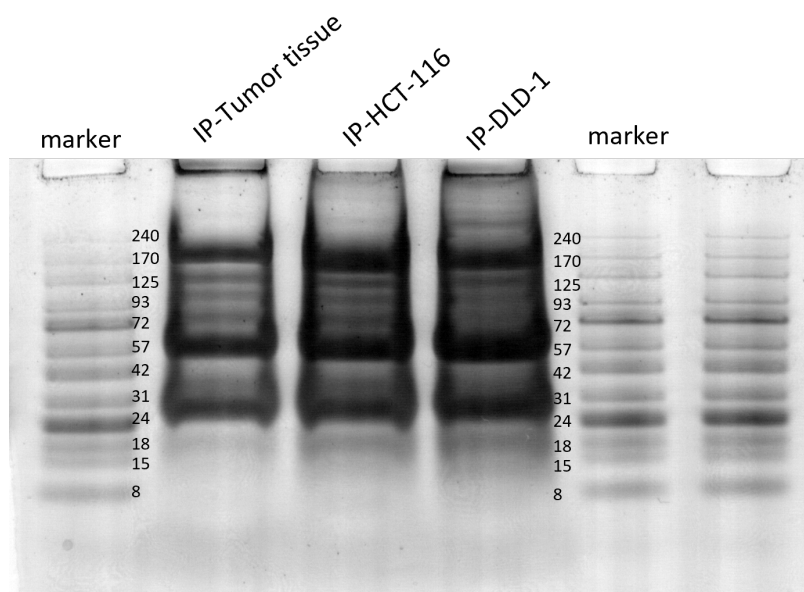

Supplement: Supplementary file 17 — Unprocessed western blots and/or gels. [file 41594_2023_1117_MOESM17_ESM.pdf]

Full unedited gel for extended figure 4d

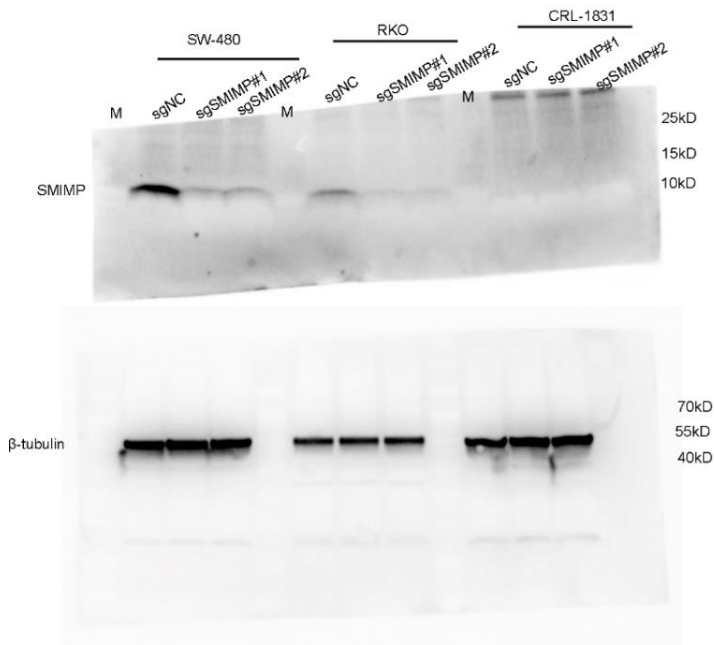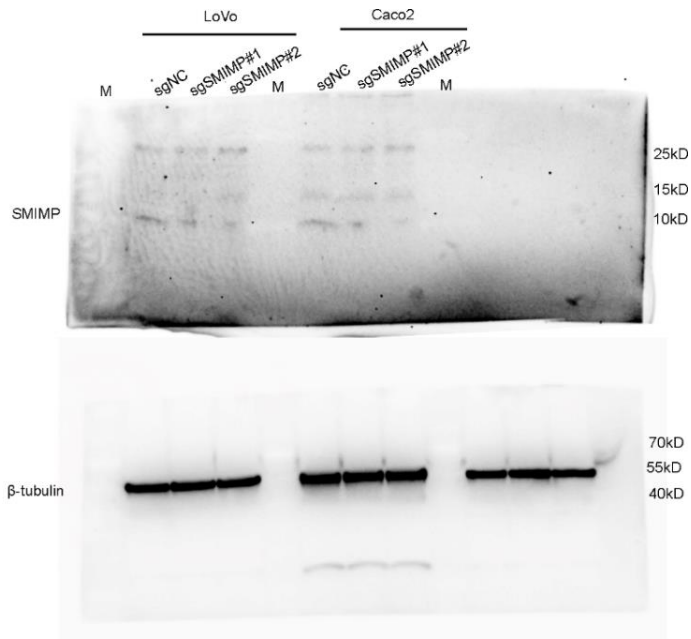

Supplement: Supplementary file 19 — Unprocessed western blots and/or gels. [file 41594_2023_1117_MOESM19_ESM.pdf]

Full unedited gel for extended data figure 10.

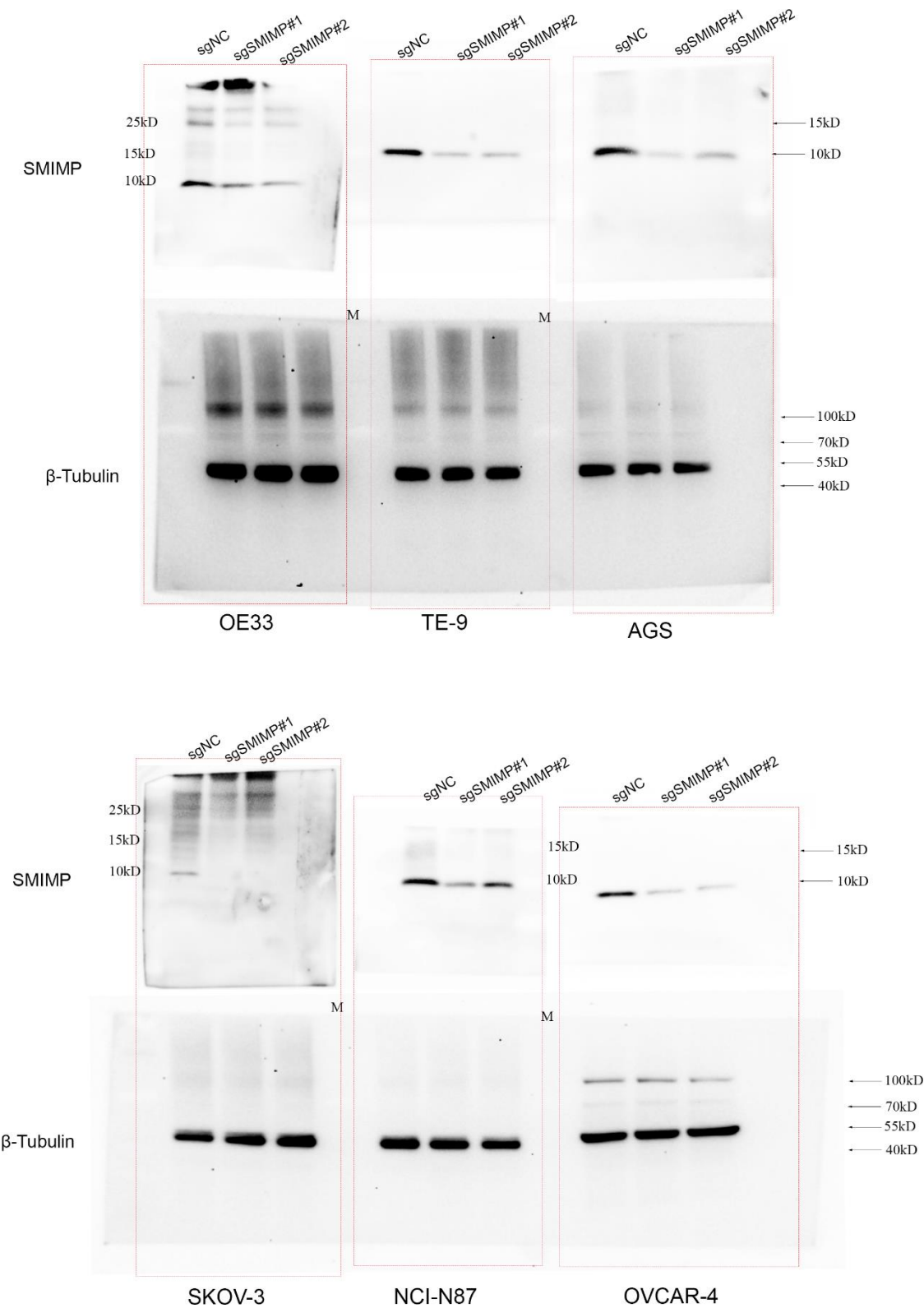

Supplement: Supplementary file 27 — Unprocessed western blots and/or gels. [file 41594_2023_1117_MOESM27_ESM.pdf]
