# Supplementary material for: CRISPR–Cas9-based functional interrogation of unconventional translatome reveals human cancer dependency on cryptic non-canonical open reading frames
Source: Nat Struct Mol Biol. 2023 Nov 6;30(12):1878–92. doi: 10.1038/s41594-023-01117-1 (PMC10716047; doi:10.1038/s41594-023-01117-1)

Full unedited gel for Figure 3a

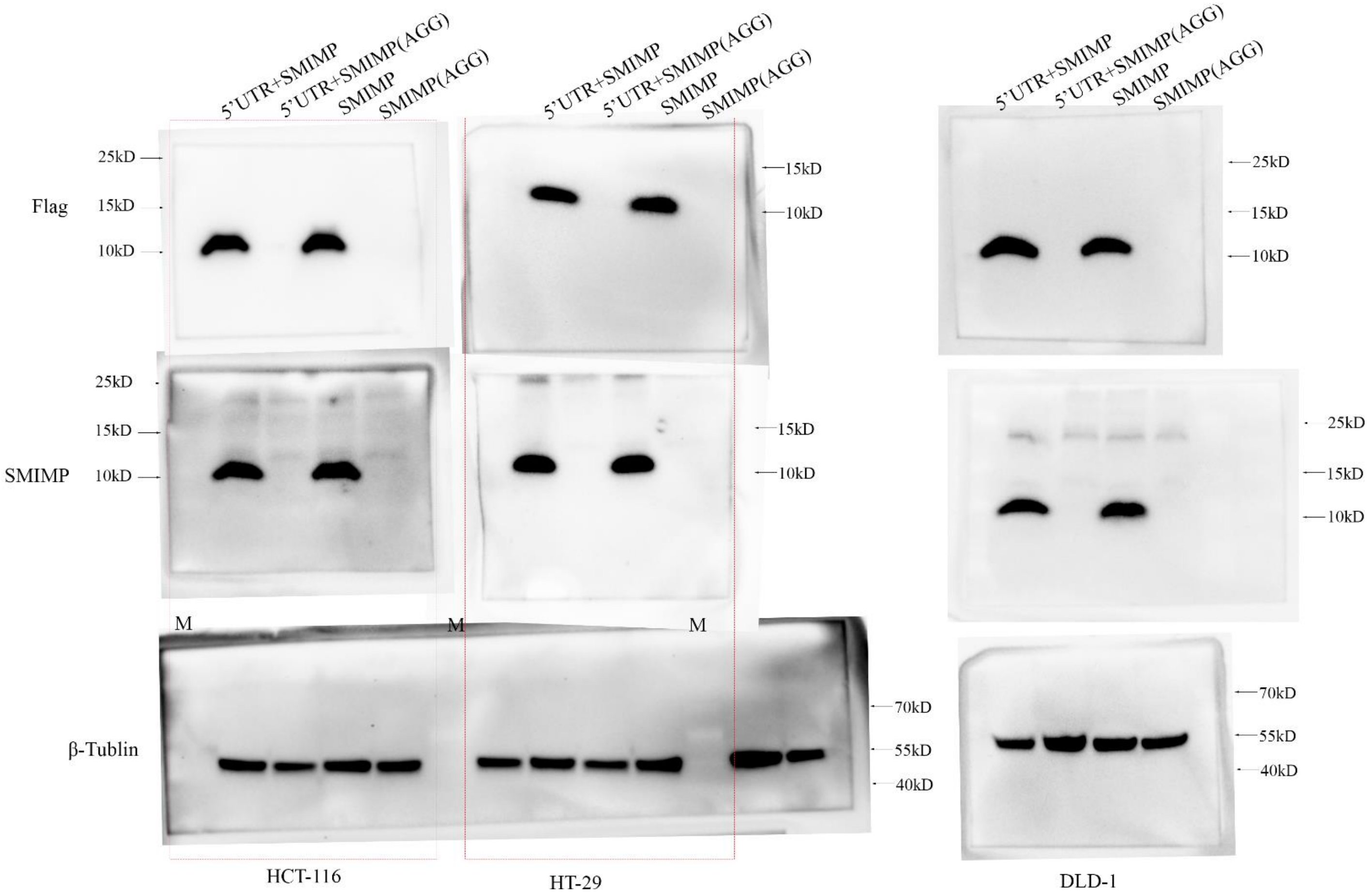

Full unedited gel for Figure 3b

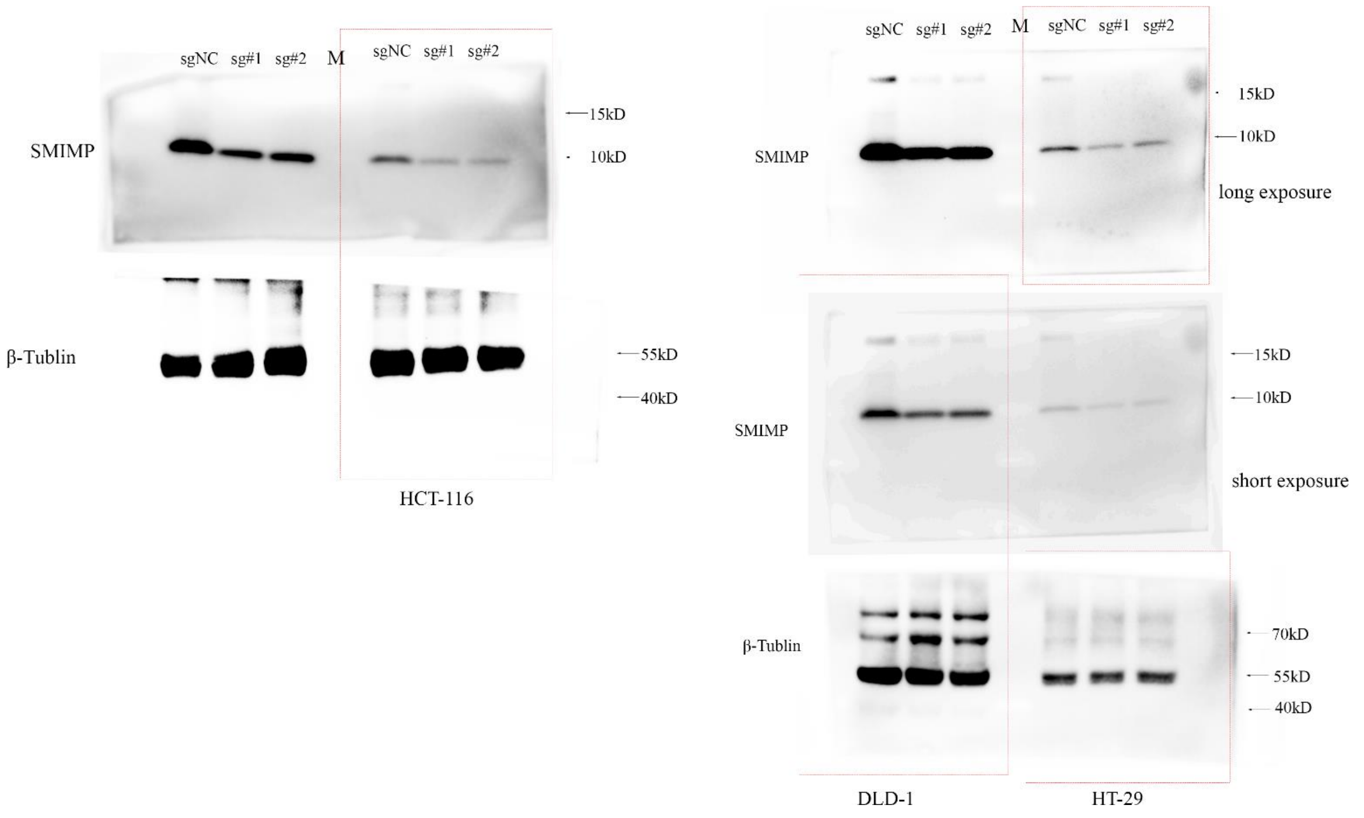

Full unedited gel for Figure 3g

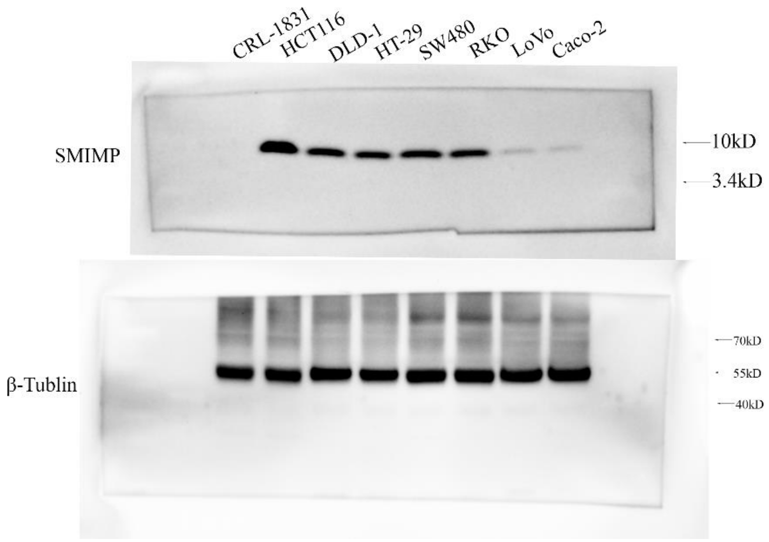

Full unedited gel for Figure 3h

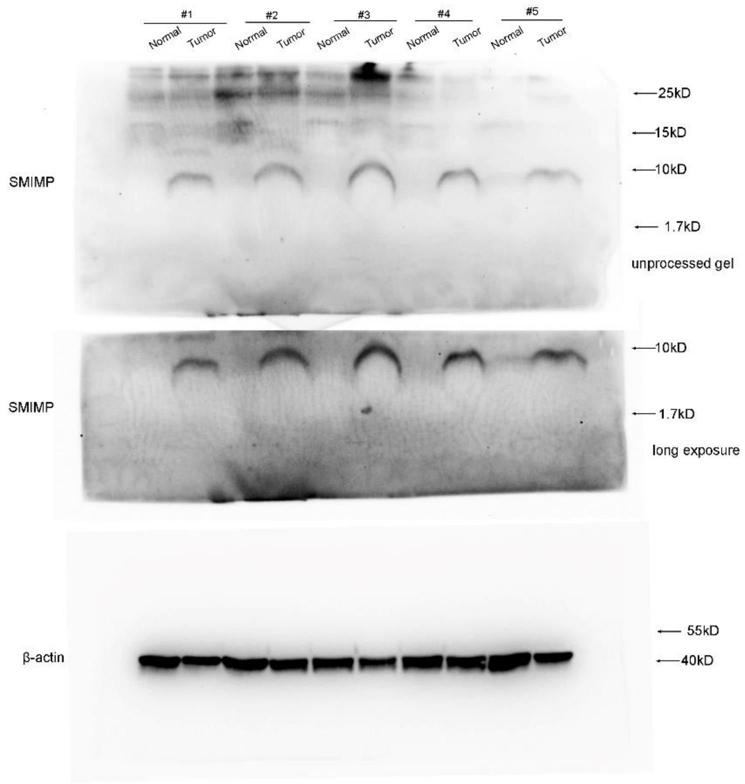

Supplement: Supplementary file 6 — Unprocessed western blots and/or gels. [file 41594_2023_1117_MOESM6_ESM.pdf]
