# Supplementary material for: CRISPR–Cas9-based functional interrogation of unconventional translatome reveals human cancer dependency on cryptic non-canonical open reading frames
Source: Nat Struct Mol Biol. 2023 Nov 6;30(12):1878–92. doi: 10.1038/s41594-023-01117-1 (PMC10716047; doi:10.1038/s41594-023-01117-1)

Full unedited gel for Figure 5a

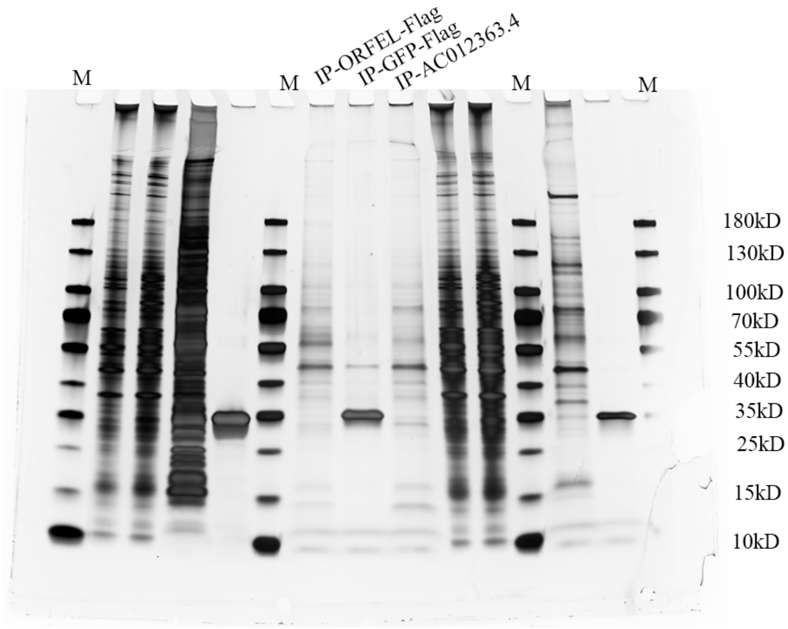

Full unedited gel for Figure 5b

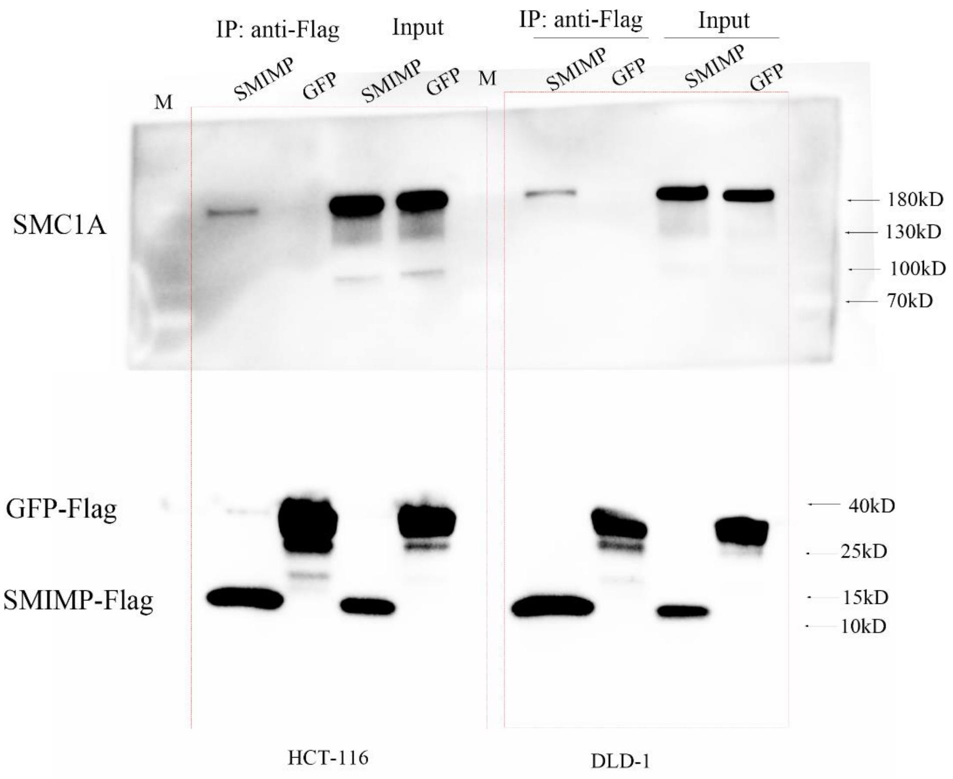

Full unedited gel for Figure 5c

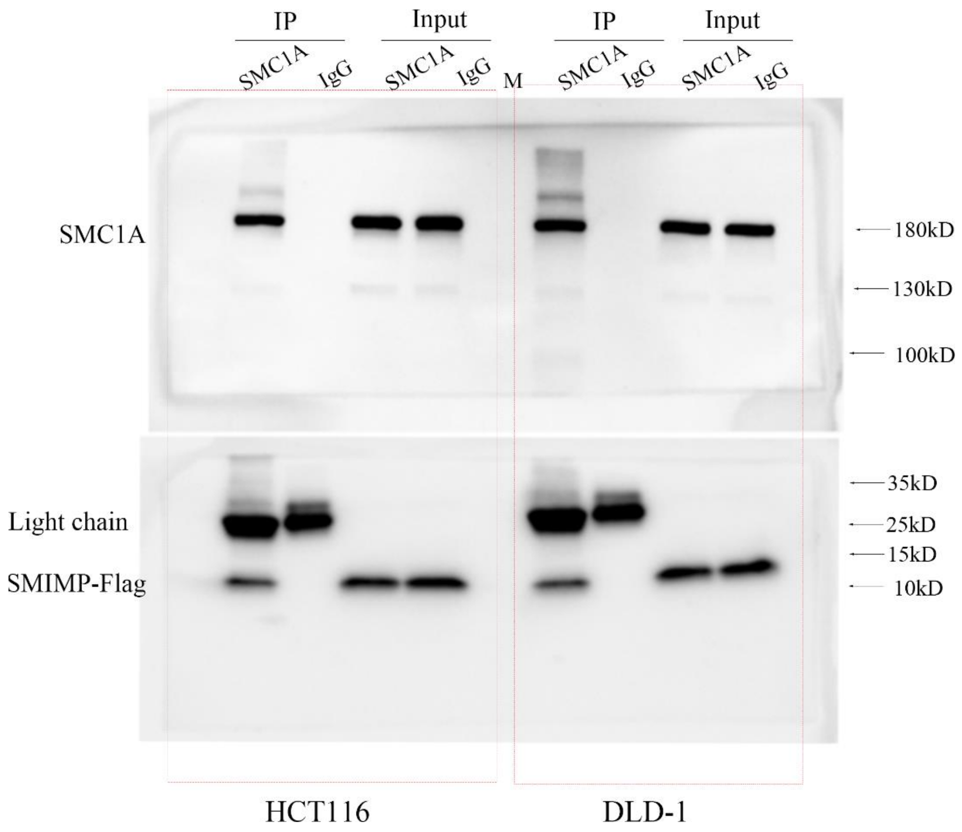

Full unedited gel for Figure 5d

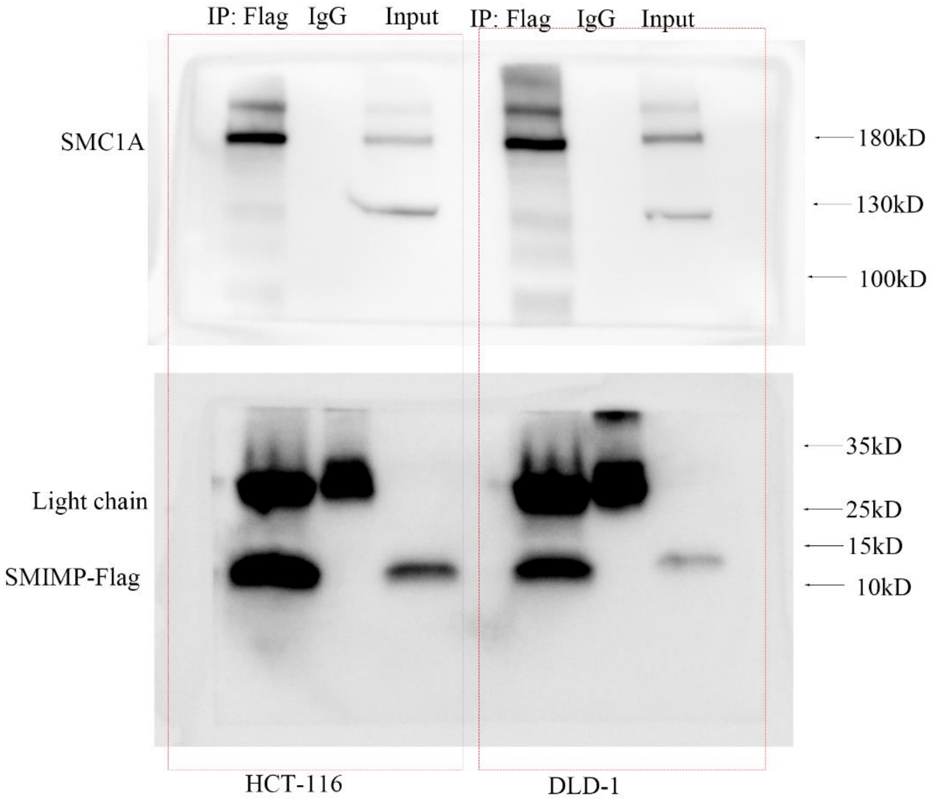

Full unedited gel for Figure 5f

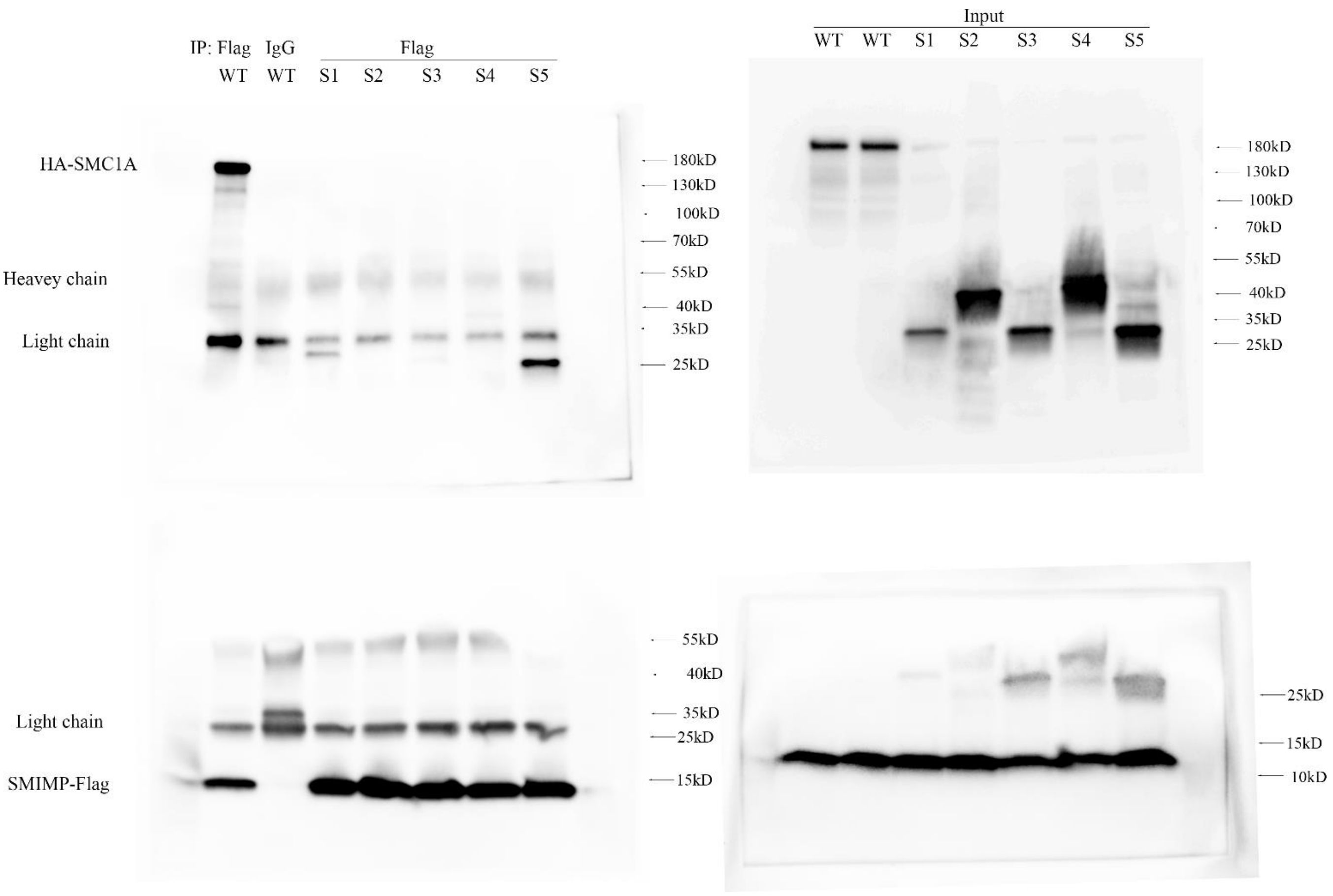

Full unedited gel for Figure 5h

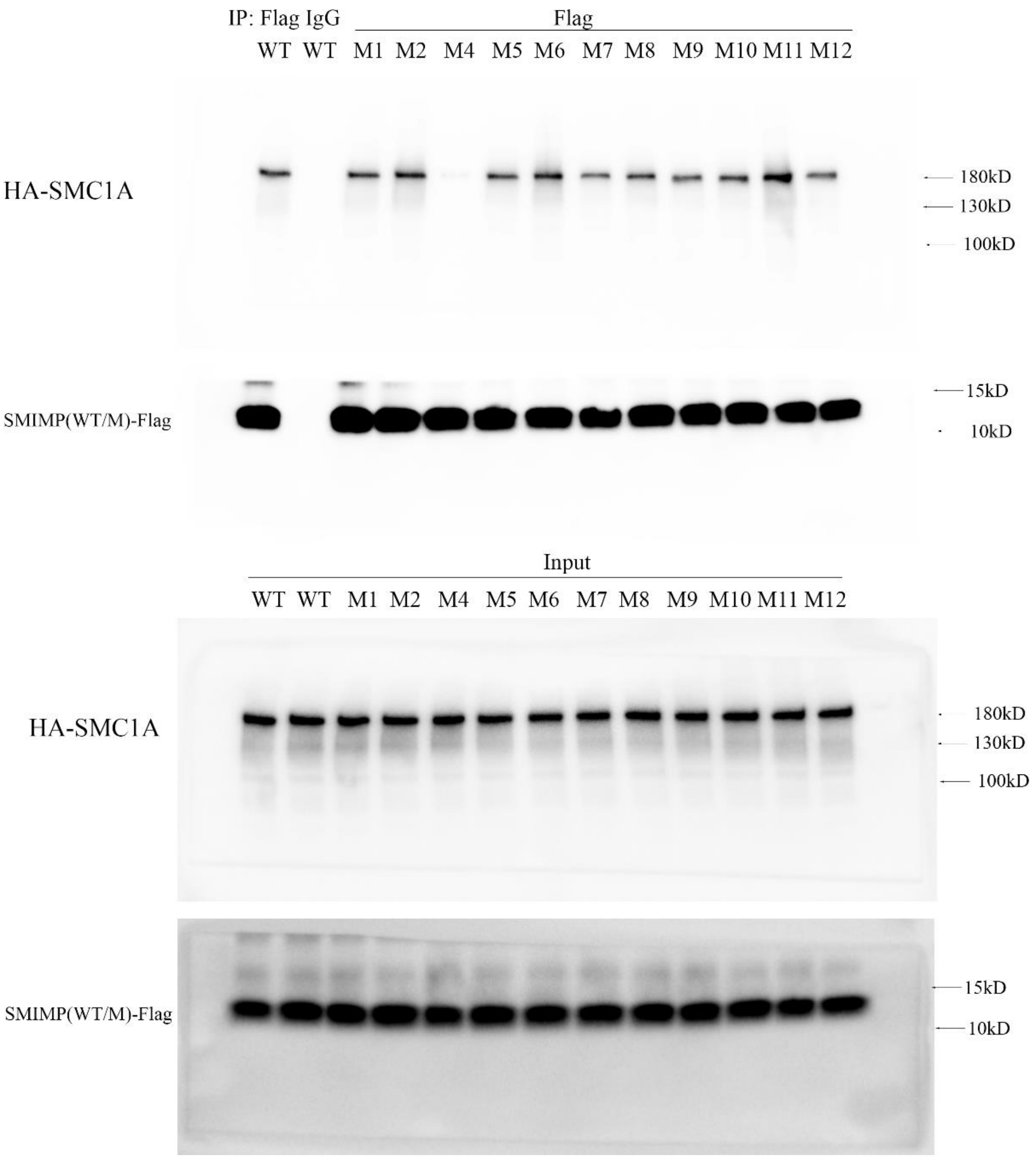

Supplement: Supplementary file 9 — Unprocessed western blots and/or gels. [file 41594_2023_1117_MOESM9_ESM.pdf]
