# Supplementary material for: CRISPR–Cas9-based functional interrogation of unconventional translatome reveals human cancer dependency on cryptic non-canonical open reading frames
Source: Nat Struct Mol Biol. 2023 Nov 6;30(12):1878–92. doi: 10.1038/s41594-023-01117-1 (PMC10716047; doi:10.1038/s41594-023-01117-1)

Full unedited gel for extended data figure 2a

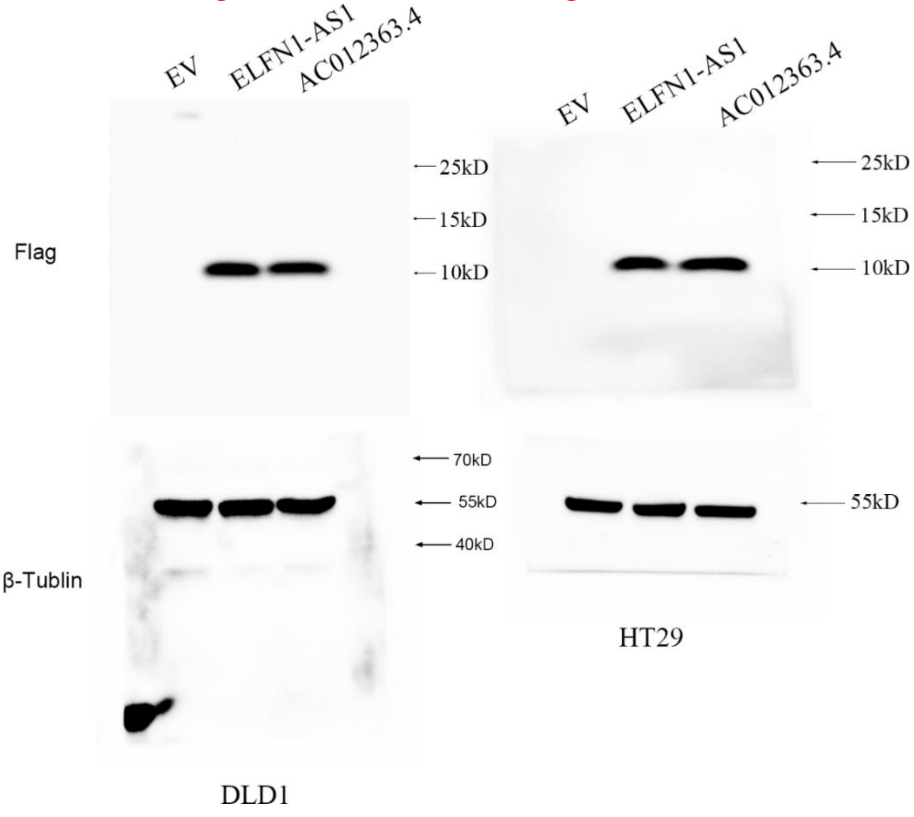

Full unedited gel for extended data figure 2b

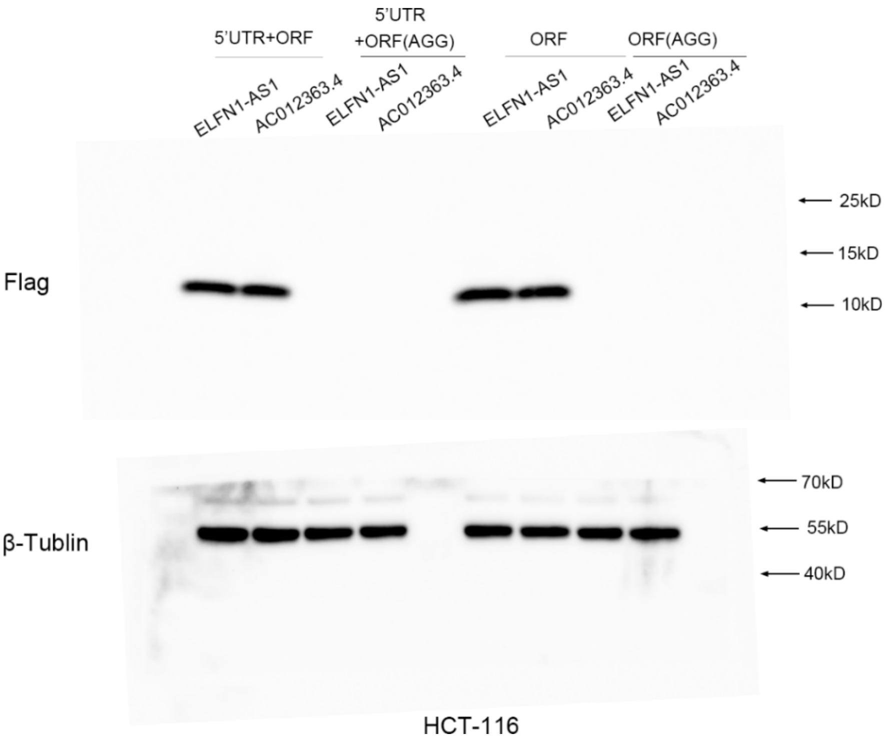

Full unedited gel for extended data figure 2c

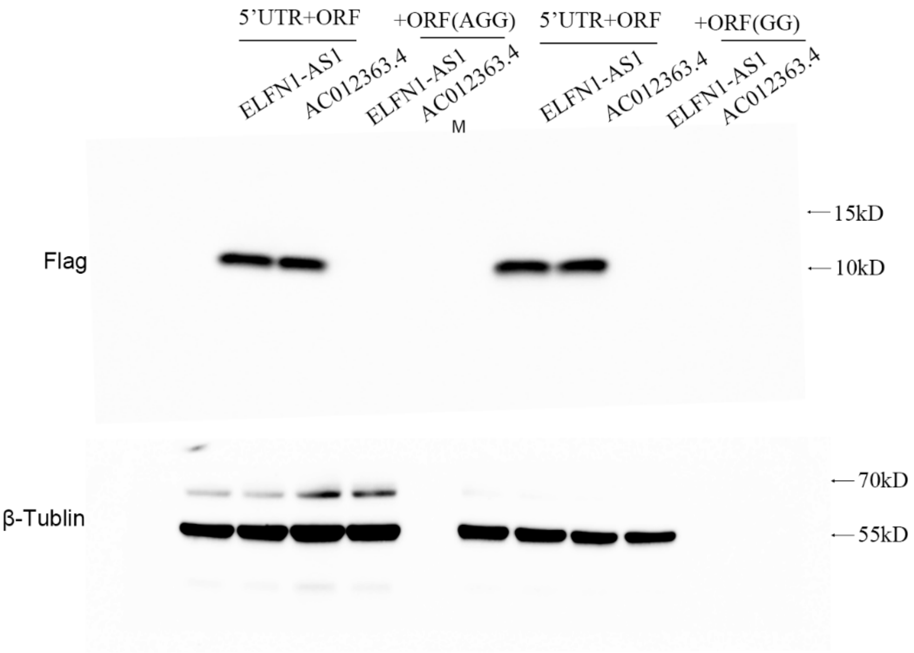

Full unedited gel for extended data figure 2d

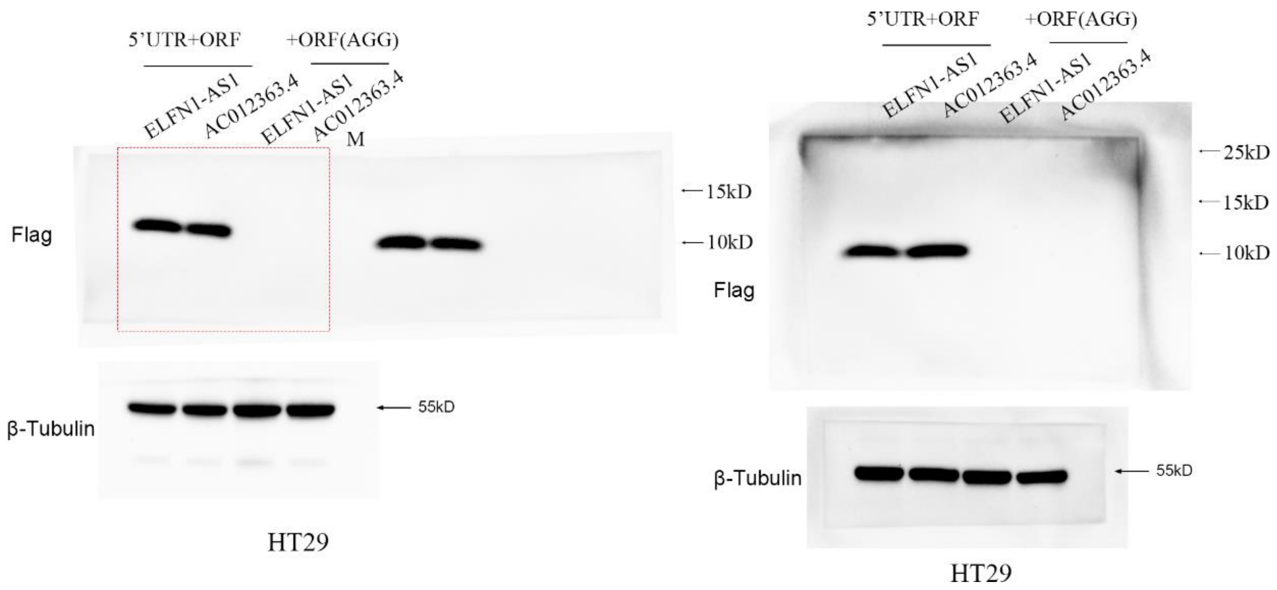

Full unedited gel for extended data figure 2k and 2l

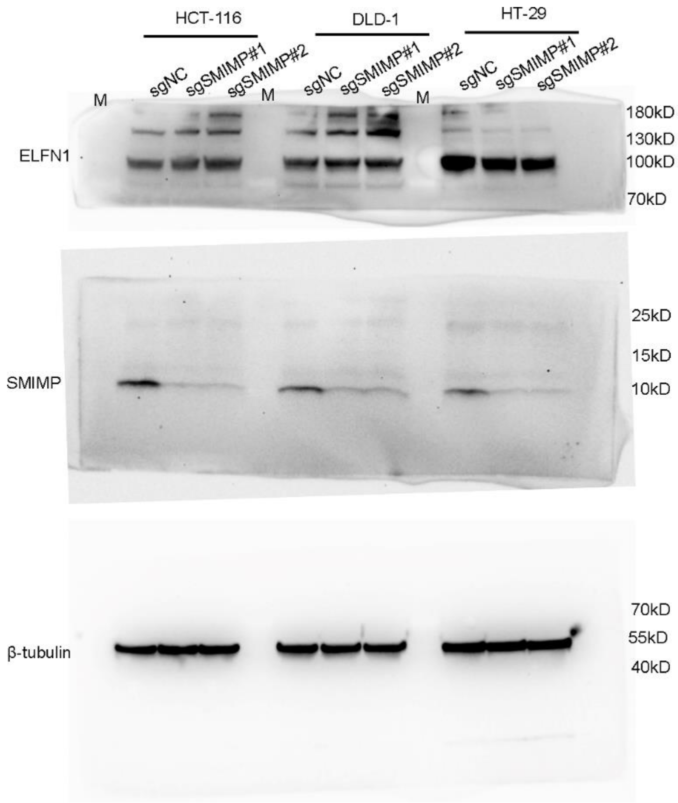

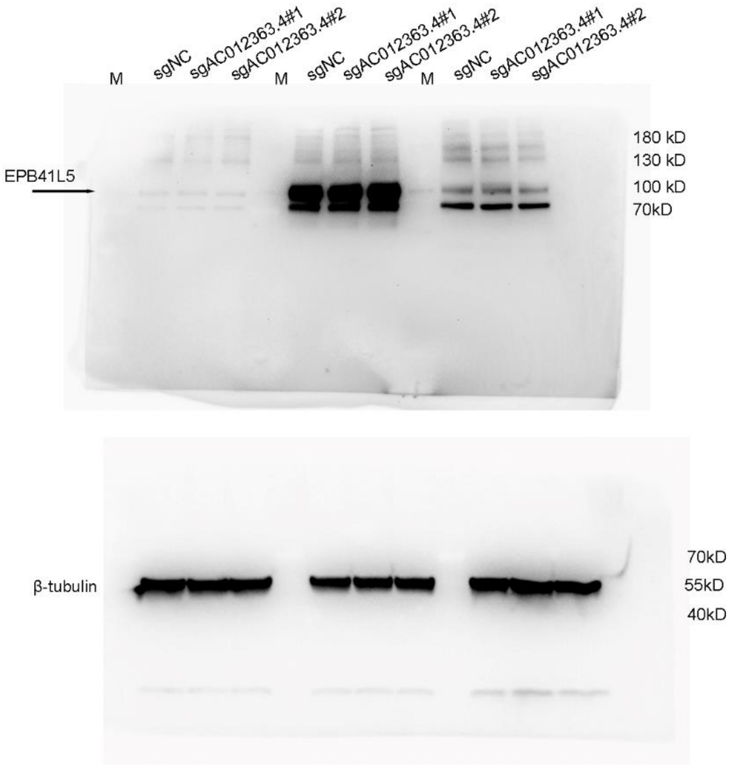

Supplement: Supplementary file 16 — Unprocessed western blots and/or gels. [file 41594_2023_1117_MOESM16_ESM.pdf]
