# Supplementary material for: CRISPR–Cas9-based functional interrogation of unconventional translatome reveals human cancer dependency on cryptic non-canonical open reading frames
Source: Nat Struct Mol Biol. 2023 Nov 6;30(12):1878–92. doi: 10.1038/s41594-023-01117-1 (PMC10716047; doi:10.1038/s41594-023-01117-1)

Full unedited gel for extended data figure 6a

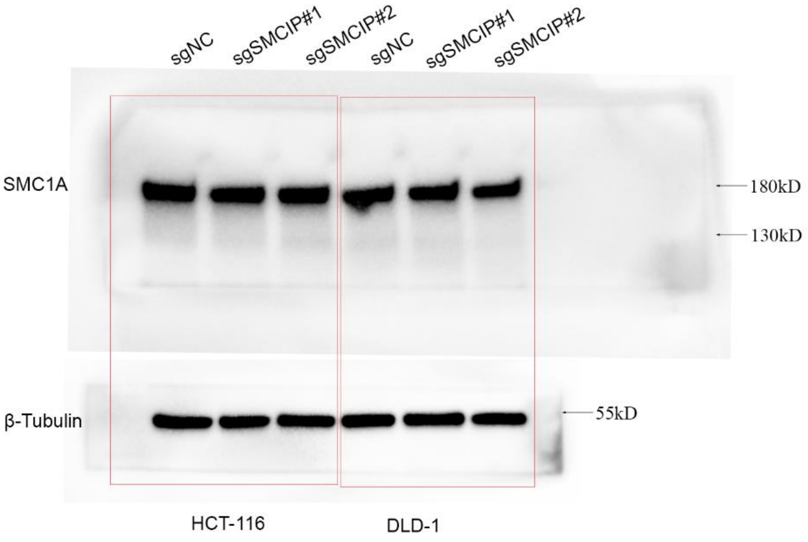

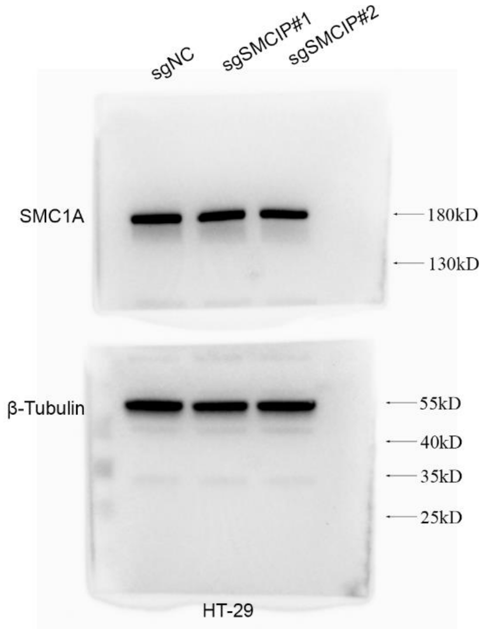

Full unedited gel for extended data figure 6b

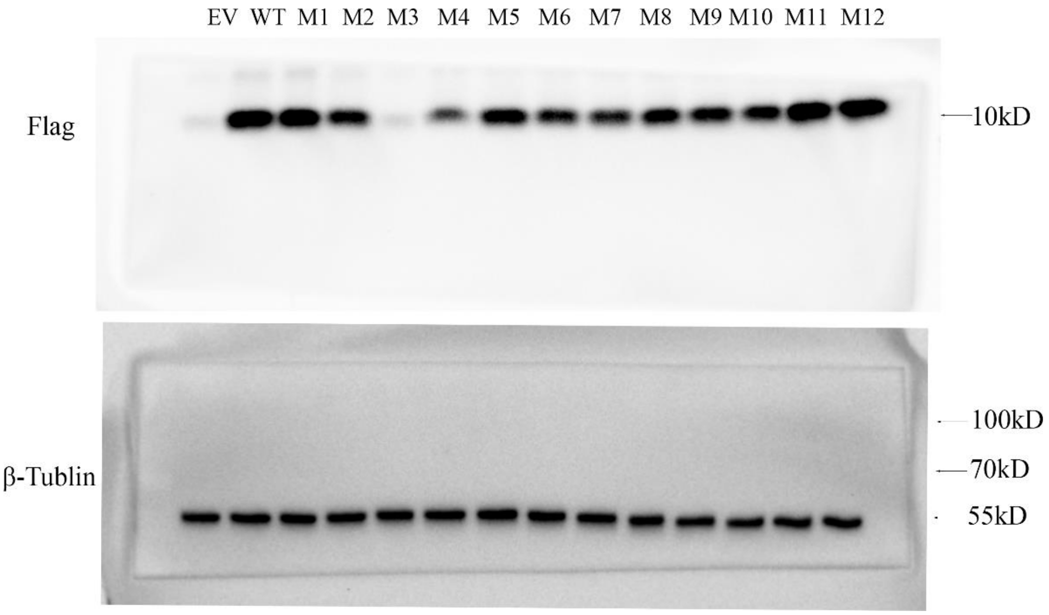

Full unedited gel for extended data figure 6c

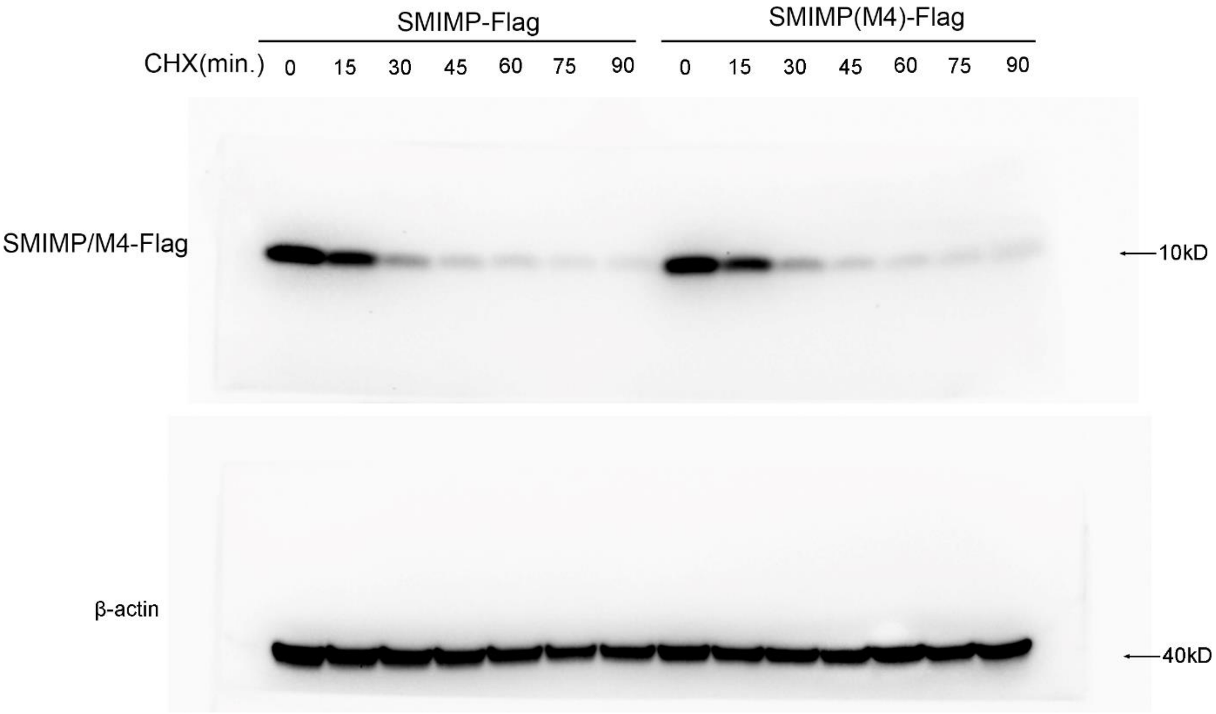

Full unedited gel for extended data figure 6e and 6g

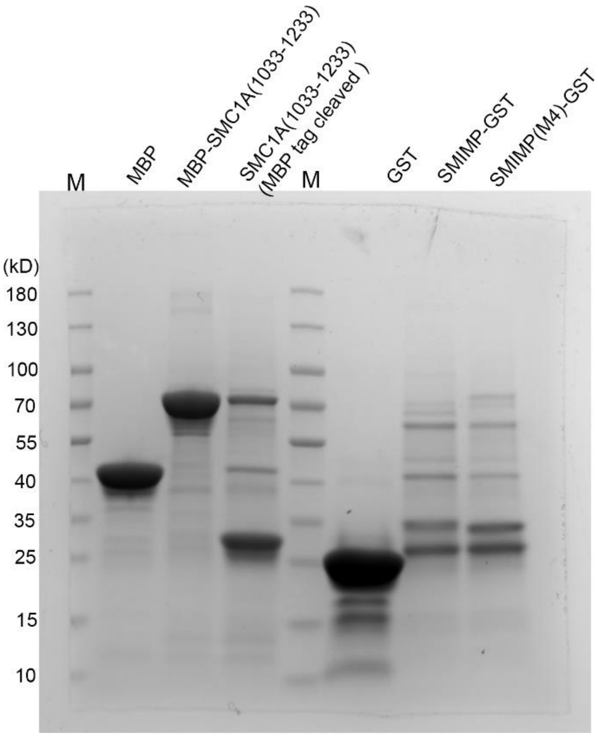

Full unedited gel for extended data figure 6f

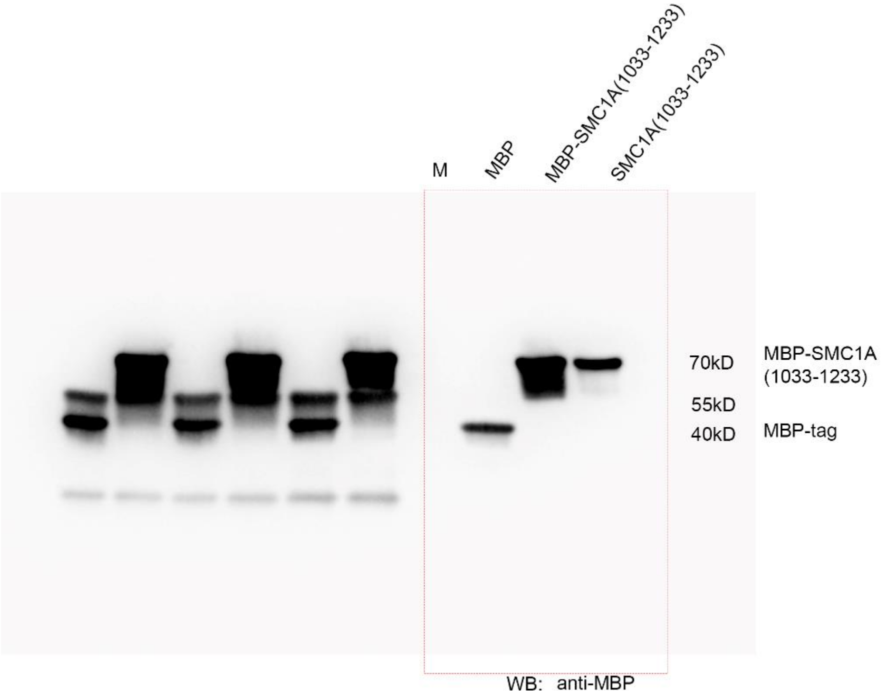

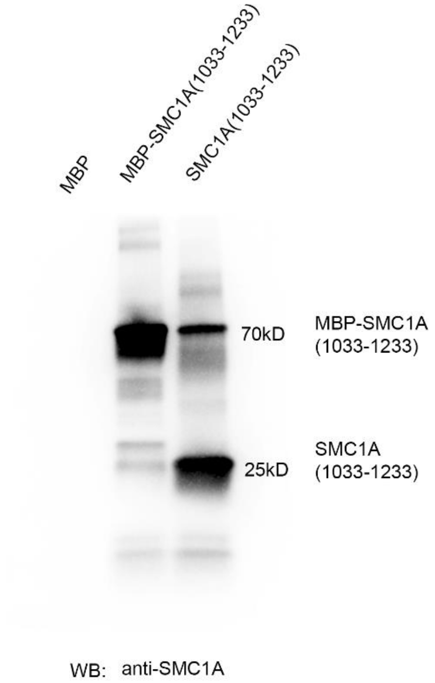

Full unedited gel for extended data figure 6h and 6i

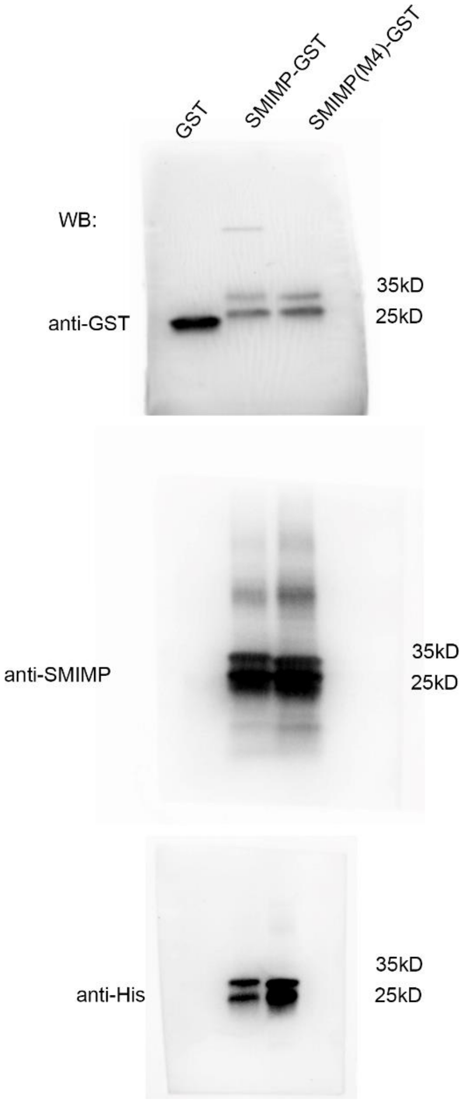

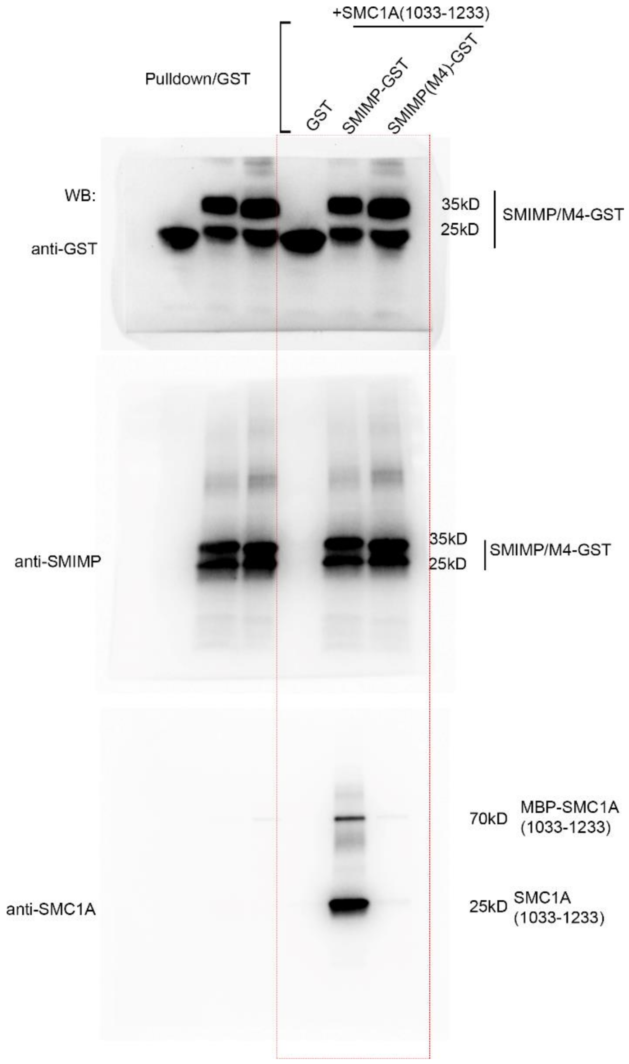

Full unedited gel for extended data figure 6j

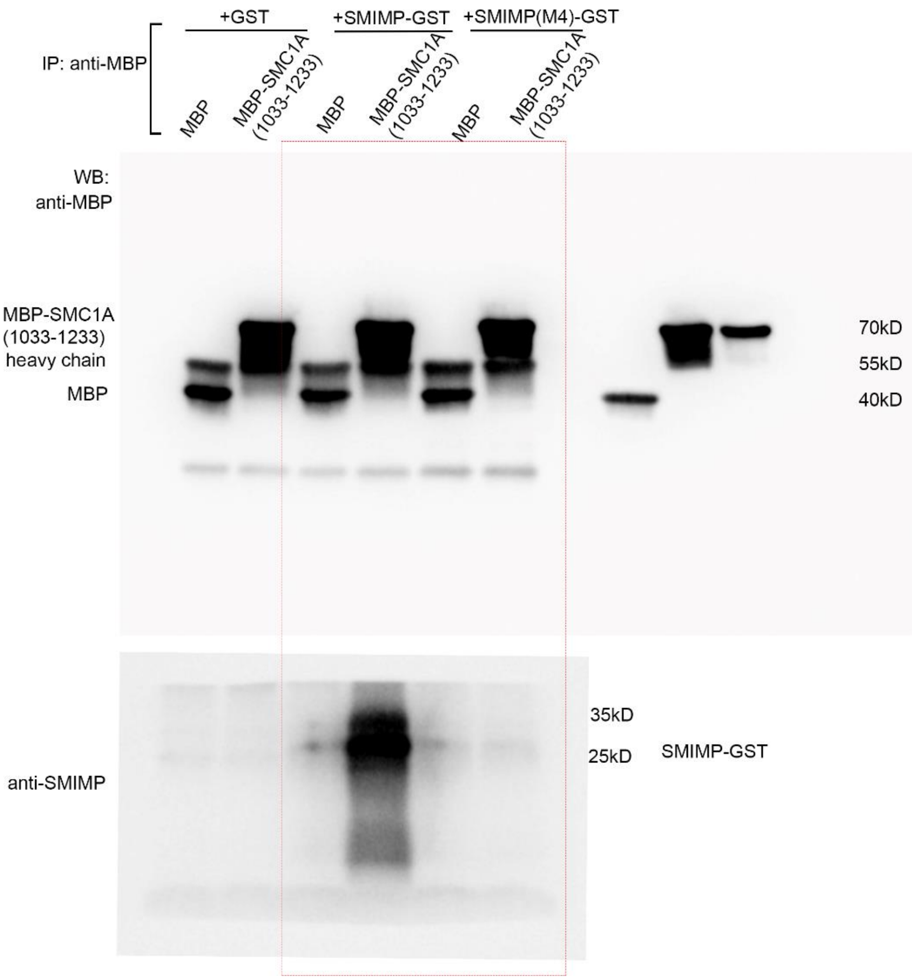

Supplement: Supplementary file 22 — Unprocessed western blots and/or gels. [file 41594_2023_1117_MOESM22_ESM.pdf]
